# International depiction of the cost of functional independence limitations among older adults living in the community: a systematic review and cost-of-impairment study

**DOI:** 10.1186/s12877-022-03466-w

**Published:** 2022-10-22

**Authors:** Ryan S. Falck, Alexis G. Percival, Daria Tai, Jennifer C. Davis

**Affiliations:** 1 University of British Columbia, Centre for Hip Health and Mobility, Vancouver, British Columbia Canada; 2 University of British Columbia, Djavad Mowafaghian Centre for Brain Health, Vancouver, British Columbia Canada; 3 Aging, Mobility and Cognitive Neuroscience Laboratory, Department of Physical Therapy, Faculty of Medicine, University of British Columbia, Vancouver, British Columbia Canada; 4 Applied Health Economics Laboratory, Faculty of Management, University of British Columbia – Okanagan, 1137 Alumni Avenue, Kelowna, BC V1V 1V7 Canada; 5 Social & Economic Change Laboratory, Faculty of Management, University of British Columbia – Okanagan, Kelowna, British Columbia Canada

**Keywords:** Functional independence limitations, Cost-of-impairment, Systematic review, Older adults

## Abstract

**Background:**

Functional independence limitations restrict older adult self-sufficiency and can reduce quality of life. This systematic review and cost of impairment study examined the costs of functional independence limitations among community dwelling older adults to society, the health care system, and the person.

**Methods:**

Following the Preferred Reporting Items for Systematic Reviews and Meta-Analyses (PRISMA) guidelines this systematic review included community dwelling older adults aged 60 years and older with functional independence limitations. Databases (Cochrane Database of Systematic Reviews, EconLit, NHS EED, Embase, CINAHL, AgeLine, and MEDLINE) were searched between 1990 and June 2020. Two reviewers extracted information on study characteristics and cost outcomes including mean annual costs of functional independence limitations per person for each cost perspective (2020 US prices). Quality was assessed using the *Consolidated Health Economic Evaluation Reporting Standards* (CHEERS) checklist.

**Results:**

85 studies were included. The mean annual total costs per person (2020 US prices) were: $27,380.74 (95% CI: [$4075.53, $50,685.96]) for societal, $24,195.52 (95% CI: [$9679.77, $38,711.27]) for health care system, and $7455.49 (95% CI: [$2271.45, $12,639.53]) for personal. Individuals with cognitive markers of functional independence limitations accounts for the largest mean costs per person across all perspectives. Variations across studies included: cost perspective, measures quantifying functional independence limitations, cost items reported, and time horizon.

**Conclusions:**

This study sheds light on the importance of targeting cognitive markers of functional independence limitations as they accounted for the greatest costs across all economic perspectives.

**Supplementary Information:**

The online version contains supplementary material available at 10.1186/s12877-022-03466-w.

## Background

More than a quarter of adults over 65 years report at least one functional independence limitation [1]. Morbidities associated with functional independence limitations commonly restrict older adult self-sufficiency and can reduce quality of life. They impose an increasing burden to individuals, the health care system, and society that includes the families and caregivers of older adults. Globally, the proportion of older adults make up a growing share of the population in most countries [2] and this will rise to approximately 2 billion persons worldwide by 2050 [3]. Allocating resources towards the prevention of functional independence limitations is critical to promoting healthy aging of populations; however, a first step towards this aim is to establish the economic burden of functional independence limitations.

Functional independence limitations threaten the ability for individuals to live self-sufficiently due to: 1) performance difficulties in domains such as instrumental activities of daily living (e.g. managing money, telephone use), 2) impairments in functional activities of daily living (e.g. bathing, dressing), 3) limitations in mobility and other general physical activities, or 4) cognitive difficulties [4–6]. The number of older adults with functional independence limitations is expected to increase over 300% by 2049 [7]. The long-term consequences of functional independence limitations include adverse health events such as falls, declines in physical and cognitive function, transition to resource-intensive acute and long-term care facilities, and increased mortality risk [8].

Functional independence limitations impose a high toll on society, the health care system, and the person because, regardless of the measure of health resource utilization, it signifies heightened health care demands [9]. For example, Spanish older adults with frailty and pre-frailty cost the health care system $3189 USD per year and $2648 USD per year, respectively; non-frail older adults cost the system $1568 USD per year (2020 prices) [10]. European health care and costs associated with dementia were greater than $206 billion USD (2020 prices) [11]. Despite this substantial burden associated with conditions linked to functional dependence, the global economic impact of functional independence limitations remains unknown. Understanding the costs of functional independence limitations is essential for: 1) providing benchmark data for the global impact of functional independence limitations; and 2) establishing this vulnerable population as a health priority for future primary and secondary prevention strategies.

Costs of impairment due to functional independence limitations can be evaluated from three economic perspectives: societal, health care system, and personal [12]. *Societal costs* encompass health care system costs as well as informal care costs, productivity losses, and other financial costs. *Health care system costs* include health care resource utilization costs such as visits to health care professionals (i.e., clinicians, nurses), hospital visits or admissions and laboratory tests/investigations. *Personal costs* are costs incurred the person living with the condition. These three economic perspectives serve a distinct purpose [13]; yet, no study has determined the costs of functional independence limitations from these perspectives.

Considering the burden that a loss of functional independence presents, a robust assessment of the health and economic burden of functional independence limitations for older adults will provide critical economic data for planning for the future needs of a globally aging population. The aim of this systematic review with cost-of-impairment analysis is to examine the cost of functional independence limitations among community dwelling older adults from three unique economic perspectives: societal, the health care system, and the person.

## Methods

### Literature search strategy

In accordance with PRISMA- guidelines [14], we conducted a comprehensive search of the Cochrane Database of Systematic Reviews, EconLit, NHS EED, Embase, CINAHL, AgeLine, and MEDLINE databases to identify peer-reviewed studies that reported cost outcomes published from 1990 through June 2020 in the English language. The systematic review protocol was registered with PROSPERO (CRD42021234003). Search terms and MEdical Subject Headings included *functional independence limitations$, cost$ and older adults.* Reference lists of included articles were manually searched to retrieve any relevant papers.

### Study selection

**Fig. 1:**
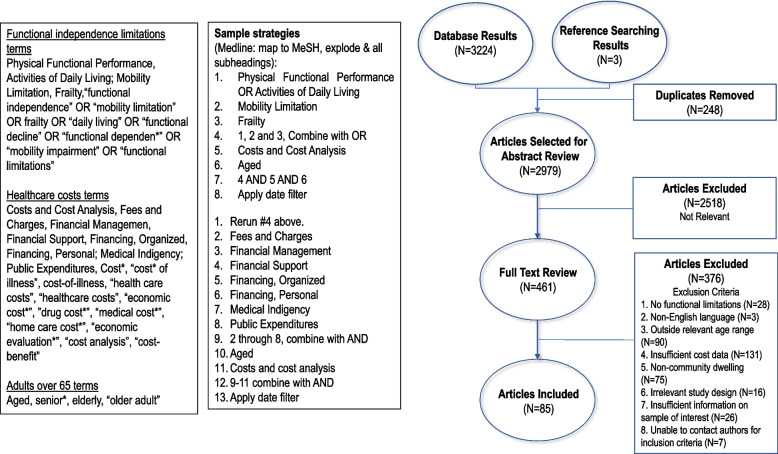
PRISMA diagram

The study selection process is detailed in Fig. 1. Studies that included an estimate of the cost of functional independence limitations in community dwelling adults aged 60 years and older were included for the following study designs: randomised controlled trials, observational studies and what we considered other (quasi-experimental, cost-effectiveness and simulation models). Studies were excluded studies if they were not a cost of illness study, did not focus on functional independence limitations, did not include an economic evaluation, had an irrelevant study design (reviews, abstracts, protocols, etc), took place in a nursing home, residential facility, hospice setting or assisted living, or we were unable to contact authors to confirm inclusion criteria.

#### Defining functional Independence limitations

Functional independence is characterized by the functional activities individuals deem fundamental to maintain their psychological and physical wellbeing [15, 16]. These functional activities are carried out synchronously through the integrated cognitive, behavioral, sensory, and motor activities [15–17]. Functional independence limitations can be comprised of physical functional limitations (e.g., difficulty with walking), chronic conditions, cognitive limitations including mild cognitive impairment and dementia, or depression in conjunction with any of the above conditions [18–25]. Mild cognitive impairment is characterized as cognitive decline greater than expected for age and education level [26]. Dementia is defined as cognitive impairment which affects several domains of cognitive function (e.g., memory, executive functions, processing speed) beyond what might be expected from normal aging [27].

To be classified as having functional independence limitations, every participant of included studies were required to meet at least one of the following criteria:

- < 9/12 on the Short Physical Performance Battery (SPPB) [28, 29]. Older adults who score ≤ 9/12 on the SPPB have a 1.6–4.9 times greater risk of disability or institutionalization [30].
- Any one of the following 9 chronic conditions asked about in NHANES: arthritis, cancer, cardiovascular disease, chronic kidney disease, depression, diabetes, hypertension, pulmonary disease, and stroke [31]. Each of these chronic conditions contributes significantly to the probability of disability [24].
- NHANES questionnaire-score indicating functional limitations [31]
- Instrumental Activities of Daily Living (IADL)-The (Lawton) Instrumental Activities of Daily Living (IADL) ranges from 0 (worst, dependent) to 8 (best, independent). A score of 7, used as a cut-off, indicates someone who is largely independent but cannot manage finances or perform housekeeping tasks [32]
- Timed Up and Go (TUG)- < 15 s [33]
- Mild Cognitive Impairment (MCI)- indicated by a score of < 26 on the Montreal Cognitive Assessment (MoCA) [34]. MCI is associated with substantially poorer performance on the IADL compared to older adults without MCI [35].
- Mini-Mental State Examination (MMSE) < 24 [36]
- Depression, Geriatric Depression Scale (GDS) > 5 [37] and one of the above measures. Higher depressive symptoms are associated with substantially greater disability burden [25].

### Data extraction and analysis

We (JCD, RSF, LP, DT) developed a standardized data extraction form in order to obtain relevant information from each study including: publication, country, study design, study sample characteristics (size, age, percentage female, disease status, and number of chronic conditions according to NHANES), frailty characteristics (IADL/ADL, FAI, Barthel Index, GFI), mobility characteristics (SPPB, TUG, 6 MW), global cognition (MMSE, MoCA, GDS), cost perspective, currency, year of currency, time period costs were measured, discounting, total cost outcomes related to impaired or loss of functional independence, societal costs, health care system costs, personal costs, and quality assessment based on the CHEERS checklist [38]. Mean and standard deviation were extracted where available.

Cost items from each study were then categorized by the three economic perspectives. The societal perspective is the broadest and captured costs including total health care system costs in addition to informal care costs, assistive services, productivity losses, and other financial costs. For example, some informal care costs included assisted services or home health care such as: community nurse home visits, home help (meals-on-wheels), and transportation. The health care system *costs* include health care resource utilization costs such as visits to health care professionals (i.e., clinicians, nurses), hospital visits or admissions, outpatient services, and laboratory tests/investigations. Personal costs included costs that resulted in out-of-pocket expenses by the individual. Some examples of these costs were medications, informal care, paid domestic home help or home care, direct health care costs. Cost item overlap does occur between some of these perspectives. For example, home help may occur under personal and societal because the societal perspective accounts for additional informal care. Missing information was obtained through contacting corresponding authors. Two authors performed data abstraction (LP, DT) and a third author (one of JCD, RSF) independently checked the data abstracted. Any discrepancies were reviewed by all authors.

### Standardized cost outcomes

Studies reported costs in different currencies and different years. To attenuate this variation, we report costs as the value documented by year and currency and we convert all costs to 2020 prices (using Purchasing Power Parity (PPP) values to convert the currency to US dollars and then inflating this value to 2020 prices using the US Department of Labor consumer price index. Any adjusted costs reflect 2020 US dollars and all non-adjusted costs represent values in the base year of the publication. For studies that did not explicitly state year of currency used, the year of currency was assumed to be the same as the study base year.

### Cost analyses

All cost analyses were conducted in R version 4.0.3 base package and can be found on GitHub (https://github.com/ryanfalck/Economic-Costs-of-Functional-Independence-Limitations). We determined the average individual costs of functional independence limitations for societal, health care system, and personal costs. For RCTs, we averaged the costs of functional impairments across all treatment groups. For observational studies which reported stratified costs (e.g., individuals with different severities of dementia), we averaged the stratified costs into a single cost estimate. The total costs from each perspective were extracted from all studies that calculated total costs. All costs were standardized to annual costs per person. Mean annual costs per person, annual costs per person for all sub-domains and their 95% confidence intervals were calculated for studies which estimated total costs for each cost perspective (i.e., societal, health care system, and personal). Mean and standard deviation were selected as the studies included had sufficient sample sizes to assume these estimates would be robust to any departures from normality.

### Quality of studies

We assessed the quality of each study using a design-relevant selected series of questions from the CHEERS checklist [38]. Two authors (LP and DT) independently evaluated each study and any discrepancies were reviewed by a third party (one of RSF or JCD). We allocated a ‘+’ indicating the item was addressed by the authors or a ‘-’ indicating the item was not addressed.

## Results

### Description of identified articles

After critical review of the 2846 titles and abstracts and 419 full text manuscripts, 85 studies met our inclusion criteria (Fig. 1) [11, 39–123]. Thirty-six studies used a societal perspective [39, 40, 43–45, 47, 48, 51, 57, 59, 63, 65, 66, 71, 73, 75, 77, 79–81, 85, 91, 93–95, 99, 101, 106, 110, 111, 113, 114, 119–123], 74 studies used a health care system perspective [11, 39–51, 54–58, 60–75, 77–81, 83–87, 89, 90, 92, 93, 95–114, 116, 117, 119, 120, 122, 123], and 45 used a personal perspective [11, 39, 43, 46–48, 51–53, 57, 60, 61, 64–66, 68–72, 75, 79, 80, 82, 88–90, 93, 94, 100, 101, 103–106, 108, 110–117, 123]. Twenty studies reported all three perspectives [39, 43, 47, 48, 51, 57, 65, 66, 71, 75, 79, 80, 93, 101, 106, 110, 111, 113, 114, 123].

### Characteristics of the studies

Appendix Table 1 describes study characteristics. Mean age ranged from 61.2 to 85 years old. Females comprised 27 to 100% of samples. Measures of functional independence limitation measures were categorized into groups: global cognition (MMSE), frailty (IADL/ADL, FAI, Barthel Index, GFI), and mobility (SPPB, TUG, 6 MW). Thirty-five studies reported global cognition [11, 39, 41, 47, 51, 55, 59, 62, 63, 65, 66, 69, 70, 75, 76, 78, 80, 85, 86, 88, 89, 91, 92, 94, 96, 98, 101, 104, 106–108, 110, 115–117, 121, 123], 55 studies reported frailty characteristics [11, 39–43, 46, 47, 49–52, 54, 58, 61–64, 68, 71–83, 86, 87, 90–94, 98–100, 107–112, 114, 115, 118–123], six reported mobility characteristics [54, 55, 63, 97, 101, 117].

### Costs of functional limitations from three cost perspectives: societal, health care system and personal

Appendix Table 2 describes the costs of functional limitations by cost perspective. Fourteen studies [47, 48, 51, 59, 63, 66, 75, 80, 81, 91, 110, 114, 119, 121, 123] provided a total value for societal costs which encompassed health care system costs as well as informal care, assistive services costs, and productivity losses. Sixteen studies estimated the costs of assisted services [39, 43, 48, 63, 65, 71, 79, 85, 95, 99, 101, 106, 113, 114, 119, 123], while 21 studies estimated the costs of home health care [39, 40, 44, 48, 57, 63, 65, 71, 73, 77, 79, 93, 94, 101, 106, 111, 113, 114, 120, 122, 123].

Forty-five studies [40–45, 47–51, 54–57, 62, 66–70, 72, 73, 75, 78, 80, 84–87, 89, 90, 92, 93, 96, 97, 103–105, 107, 109, 111, 112, 116, 117, 120, 123] provided a total value for health care system costs which may consist of inpatient, hospital, health care professional (physician), outpatient/ambulatory, specialist, GP, nurse, emergency, physiotherapy, intervention costs or any other items that authors deemed health care system costs. Twenty-nine studies reported the costs of clinicians [44, 48, 61, 63, 65, 71, 72, 77, 79, 81, 85, 93, 95, 99–101, 104–106, 108, 110, 111, 113, 114, 117, 119, 120, 122, 123], hospital costs were reported in 45 studies [11, 39, 40, 42–44, 48, 49, 51, 54, 57, 58, 63–65, 71–74, 77–79, 81, 83, 84, 86, 87, 89, 90, 92, 93, 95, 98, 99, 101, 102, 104, 106, 110, 111, 113, 114, 117, 119, 122], and outpatient costs were estimated in 33 studies [11, 39, 40, 42, 44, 48, 51, 57, 60, 62–64, 71, 73, 74, 76–79, 85, 87, 92, 95, 100, 101, 104, 106, 107, 110, 111, 113, 117, 120, 122].

Nine studies [47, 48, 51, 61, 68, 72, 80, 93, 108, 123] estimated a total value for personal costs which may encompass costs for pharmaceuticals/medications, informal care, paid domestic help/home care (non-clinical help), home modifications or any other items that authors deemed personal costs. Twenty-two studies reported costs of medications [11, 51, 53, 57, 64, 72, 75, 79, 89, 90, 93, 100, 104, 106, 108, 110–113, 116, 117, 123], 28 studies reported home help indirect costs [11, 39, 43, 46, 51, 52, 66, 69, 70, 72, 75, 82, 88, 93, 94, 100, 101, 103, 105, 106, 110, 112–116, 123], and eight studies reported direct health care related costs [52, 61, 65, 72, 73, 90, 103, 123].

### Cost analyses

Our cost analyses for each perspective and sub-domain are described in Appendix Table 3 and Table 4. From the 14 studies reporting total societal costs of functional independence limitations, the mean annual cost per person was $27,380.74 (95% CI:[$4075.53, $50,685.96]) with an estimated standard deviation of costs of $33,249.11 (95% CI:[$7228.24, $59,269.99). Mean societal costs based on frailty markers (*n* = 11) were $30,045.48 (95% CI:[$482.68, $59,608.28]), $3135.51 based on mobility indices (*n* = 1), and $32,623.61 (95% CI:[$346.64, $64,900.58]) based on cognitive markers of functional independence limitations (*n* = 10). The mean annual home health care costs per person were $3370.50 (95% CI:[$2266.76, $4474.23]) and the mean assisted services costs per person were $3463.14 (95% CI:[$335.50, $6590.79]. Standard deviations were $4453.91 (95% CI:[$2941.22, $5966.60]) for home health care and $4566.43 (95% CI:[$956.16, $8176.69]) for assisted services.

The mean annual cost per person for the health care system was $24,195.52 (95% CI: [$9679.77, $38,711.27]); the standard deviation of costs was $21,606.31 (95% CI:[$13,824.75, $29,387.88]). The mean annual cost to the health care system based on frailty markers (*n* = 25) was $21,266.01 (95% CI:[$11,763.67, $30,768.35]), $12,093.88 (95% CI:[−$7843.04, $32,030.80]) for mobility indices (*n* = 3), and $31,597.36 (95% CI:[$560.37, $62,634.35]) for cognitive markers of functional independence limitations (*n* = 20). Mean annual hospital costs per person were $16,783.25 (95% CI:[$5332.21, $28,234.29]). Mean annual clinical costs were $7357.55 (95% CI:[−$2568.05, $17,283.14]). Mean annual outpatient costs were $9401.63 (95% CI:[$835.10, $17,968.15]). Standard deviations were $18,387.83 (95% CI:[$6868.35, $29,907.31]), $7400.77 (95% CI:[−$2442.89, $17,244.43]), and $10,528.58 (95% CI:[$1589.07, $19,468.09]) for hospital, clinical, and outpatient costs, respectively.

Total personal mean annual cost per person were $7455.49 (95% CI:[$2271.45, $12,639.53]); the standard deviation of costs was estimated to be $11,208.48 (95% CI:[$5262.09, $17,154.87]). Average costs based on frailty markers (*n* = 7) were $8294.09 (95% CI:[$1670.40, $14,917.79]), and $12,628.77 (95% CI:[$2816.31, $22,441.24]) based on cognitive markers (*n* = 4); mobility measures were not reported for any study which calculated total personal costs. For home help, mean annual costs per person were $14,075.00 (95% CI:[$7294.50, $20,855.51]). For medications, mean annual costs per person were $1170.08 (95% CI:[$726.21, $1613.95]). For direct health care, mean annual costs per person were $3929.46 (95% CI:[$781.19, $7077.73]). Standard deviations were $15,851.18 (95% CI:[$9090.34, $22,612.02]), $1325.71 (95% CI:[$758.65, $1892.77]), and $4602.58 (95% CI:[$1692.77 $7512.39]) for home-help, medications, and direct health care related costs, respectively.

### Quality analysis

The quality assessment highlighted that 17 of the 85 studies met 100% of the CHEERS criteria and 60 of the 85 studies met 80% or more of the criteria (Appendix Table 5). Only 33 of the 85 studies explicitly stated the cost perspective. Another item most commonly lacking was characterization of uncertainty.

## Discussion

The mean annual cost per person was the greatest from a societal perspective estimated at $27,380.74 (95% CI:[$44075.53, $50,685.96]) and was the least from a personal perspective estimated at $7455.49 (95% CI:[$2271.45, $12,639.53]). The mean annual cost per person from a total health care system perspective was $24,195.52 (95% CI: [$9679.77, $38,711.27]); this was comparable with the societal perspective. Interestingly, the mean annual cost per person from all three economic perspectives, demonstrated that individuals with cognitive markers of functional independence limitations’ incurred the highest costs. Targeting prevention strategies for individuals with cognitive markers of functional independence limitations is essential.

Importantly, our review identified novel and distinct cost item categories within each perspective that drove the total cost estimates. For societal costs, these included home help/health services and day care. For health care system costs these included inpatient care. For personal costs, these included informal care and medications. This information is useful for elucidating which cost categories impact the individual the most (i.e., medications).

### Societal perspective

Our results demonstrate that the economic burden of functional independence limitations vary by cost perspective. Of the 45 studies that provided an estimate for the total costs of health care, only 14 estimated total societal costs. This highlights an important methodological consideration – what costs are currently not captured by many studies to adequately represent a societal perspective? At face value, it appears that societal costs were not given the attention of health care costs, and likely our estimates of cost for this perspective are low and likely represent an extremely conservative estimate of the societal perspective.

Home help/health services and assisted services accounted for approximately 25% of the total societal costs per person. From a government budget perspective, these findings emphasize the importance of informal caregiving, commonly seen as a low-cost source of care [124]. Data also indicate that we may experience limitations in the number of family caregivers available to provide care for older adults with functional limitations in the coming years [125, 126]. As such, there is a critical need to define a model of care to train and fund informal care-givers such that older adults with functional independence limitations remain supported as they age. This observation requires cautious interpretation due to the heterogeneity of the studies included. Specifically, 21 studies examined home health care costs and 16 studies estimated assisted services costs. This observed inconsistency in the cost-items reported for societal costs emphasizes the importance of developing condition specific frameworks for condition-specific cost-of-impairment studies [127].

### Health care system perspective

A lack of functional independence increases an individual’s susceptibility to complications which lead to an increase in health care system costs [98]. Older adults living with functional independence limitations consume a substantial portion of health care resources, between an estimated $9679 and $38,711 per person each year. Hospital costs are the largest health care cost which older adults with functional limitations consume, with an average $16,783 per person across the 45 studies which estimated these costs. However, outpatient costs are equally taxing on the health care system; people with functional independence limitations consume $9401.63 per person annually. The costs of functional independence limitations on the health care system are substantial.

### Personal cost perspective –an understudied perspective

Often overlooked, is the personal cost perspective. Informal care is a large home-care cost contributor sometimes making home care much more expensive than long-term institutional care. Individual’s absorb the substantial costs of unpaid caregiving. The United States had a significantly higher average annual cost per person for informal care than other locations. Informal caregivers support over 14.7 million Americans; this number is expected to grow with the growth of the aging of the population. Often informal care is a less visible part of total care; it has historically been ignored in economic evaluations and therefore policymaking [128]. As a valuable complement of formal social and health care services and a significant impact on caregiver quality of life, there needs to be more standardization as well as improved methods for measuring the value of informal care in economic evaluations.

Several important factors are essential in the comparison of costs internationally [129]. The distribution of functional independence limitations across countries are unequal and sometimes disproportionate when considering condition-specific burden. There is also an unequal distribution of data with poorer countries often under-represented. Substantial differences exist across countries with social insurance programs that cover services like impatient, homecare, certain long-term care and those that only include acute care and short term post hospitalization rehabilitation. The impact of different reimbursement systems on country specific studies included in this systematic review contribute to the important factor of “who pays.” Some countries (i.e., Southeast Asia) experience a high proportion of out-of-pocket health related expenses. Individuals living in poorer countries may pay the ultimate price of mortality or morbidity for their health due to inability to absorb these out-of-pocket expenses. The value of future country specific studies detailing these personal costs is essential both for quantifying condition-specific burden and for generating evidence to support global support efforts for those with functional independence limitations.

#### Limitations

There is no agreed upon definition for functional independence limitations, nor a consensus for what criteria meet a functional independence limitation. Thus, we used a heuristic approach for developing a working definition of functional independence limitations based on available evidence. Our results may be dependent on the criteria we developed. Countries and regions within them charge different amounts for similar services, which impeded the ability to accurately measure and compare the cost. Further, the amount of costs incurred for home and adult day care depends on a country’s insurance system. Countries and regions also have unique investment patterns for various target populations. For instance, some country health schemes do not cover non-acute conditions and local charitable organizations often have to pick up the costs for home and community-based services, food pantries and home delivered meals. These costs would substantially increase the true societal costs. As such, we report costs by country and regions within countries. Some costs of functional limitations within studies may under-represent total costs. This is partially due to the diverse physical and cognitive deficits that lead to functional limitations. This is also due to the under-reporting of secondary costs within most studies. Studies which reported stratified estimates of costs by severity of functional independence limitations were averaged across all severity levels, given that different criteria were used for classifying severity of the limitation. We postulate that all cost estimates may provide a conservative estimate of the economic burden of functional independence limitations. There was heterogeneity in the study design, assessment of functional independence limitations, measurement of cost, cost perspective and cost items measured. We addressed this by clearly stating a specific definition and criteria for functional independence limitations and reporting specific study design, cost perspective and cost items when available. However, we acknowledge that our definition of functional independence limitations may potentially group those at risk of having functional independence limitations with those who actually have functional independence limitations; consequently, our estimates may not accurately reflect the true costs of functional independence limitations. Comparing results of economic analyses across countries should be interpreted with caution due to variation among: year and currency costs were collected, populations studied, cost items reported, time horizon and cost perspective reported. To reduce this type of heterogeneity between studies we converted all estimates to a common currency and year (USD 2020 prices). Another issue worth considering is the overlap between societal and personal costs, which are complex to separate. Lastly, several of the studies which we included did not report their sample size [45, 72, 78, 79, 87, 90, 93].

## Conclusions

This study provides novel and indispensible economic data detailing the mean annual total costs per person (USD at 2020 prices): $27,380.74 (95% CI: [$4075.53, $50,685.96]) from a societal perspective, $24,195.52 (95% CI: [$9679.77, $38,711.27]) from a health care system perspective, and $7455.49 (95% CI: [$2271.45, $12,639.53]) from a personal perspective. Importantly, this study highlights, across all three economic perspectives, cognitive markers of functional independence limitations accounted for the highest mean costs per person. Three key cost drivers for individuals with functional independence limitations were: 1) home help/health services and day care drive societal costs, 2) inpatient care drive health care system costs, and 3) informal care and medications drive personal costs. A reporting consensus on the cost items that comprise the key factors driving costs would empower future cost of impairment studies with a more robust ability to estimate the global and country specific estimates for the economic burden of functional independence limitations to our economy.

## Supplementary Information

**Additional file 1: Appendix Table 1.** Characteristics of Studies. **Appendix Table 2.** Cost Items Related to Impaired or Loss of Functional Independence. **Appendix Table 3.** Mean and standard deviations of annual costs per person of functional independence limitations based on each cost perspective. **Appendix Table 4.** Mean and standard deviations of annual costs per person of functional independence limitations based on markers of frailty, mobility, or cognition and each cost perspective. **Appendix Table 5.** Modified version of CHEERS checklist to assess quality of economic and cost-of-illness studies.

**Additional file 2 Supplementary Material S1.** R code and output.

**Additional file 3: Supplementary Material S2.** Cost estimates for each study.

## Data Availability

Raw data are included as supplementary files. Further information can be obtained from authors upon request.
