# Supplementary material for: International depiction of the cost of functional independence limitations among older adults living in the community: a systematic review and cost-of-impairment study
Source: BMC Geriatr. 2022 Oct 22;22:815. doi: 10.1186/s12877-022-03466-w (PMC9587635; doi:10.1186/s12877-022-03466-w)
Supplement: Supplementary file 1 — Additional file 1: Appendix Table 1. Characteristics of Studies. Appendix Table 2. Cost Items Related to Impaired or Loss of Functional Independence. Appendix Table 3. Mean and standard deviations of annual costs per person of functional independence limitations based on each cost perspective. Appendix Table 4. Mean and standard deviations of annual costs per person of functional independence limitations based on markers of frailty, mobility, or cognition and each cost perspective. Appendix Table 5. Modified version of CHEERS checklist to assess quality of economic and cost-of-illness studies. [file 12877_2022_3466_MOESM1_ESM.docx]

**Appendix Table 1.** Characteristics of Studies

| **Publication, Country, Study Design** | **Sample Characteristics** Size Mean Age (SD) %Female Disease Status  Number of Chronic Conditions | **Frailty Characteristics** IADL/ADL, FAI, Barthel Index, GFI  Mean (SD)^1^ | **Mobility Characteristics** SPPB, TUG, 6MW   Mean (SD)^1^ | | **Global Cognition** MMSE, MoCA, GDS  Mean (SD)^1^ |
| --- | --- | --- | --- | --- | --- |
| Åkerborg 2016, Sweden, observational study | 170 N/A 66.5% Dementia  Quan-Charlson comorbidity index: 0.49 (1.37) | IADL: 3.26 (1.97)  ADL: 1.74 (2.28) | | N/A | 17.02 (7.73) |
| Akincigil 2020, USA, longitudinal cohort study | 6160 79 67% Not reported  70% of participants had ≥1 chronic condition | 2+ IADL (High Impairment): 29.3% | | N/A | N/A |
| Ankuda 2017, USA, prospective cohort study | 3101 76.21 (0.18) 42.7% 16.2% have dementia  Not reported | Number of ADL and IADL impairments: 2.75 (0.06) | | N/A |  |
| Beech 1999, England, randomized controlled trial | Size 331 - Intervention: 167 - Control: 164 Age - Intervention: 70 - Control: 72  %Female - Intervention: 45% - Control: 43% Disease Status: All subjects were stroke patients  Chronic Conditions: Not reported | Barthel Index Scores Intervention: - 0-14: 42% - 15-19: 47% - 20: 10% Control: - 0-14: 40% - 15-19: 48% - 20: 12% | | N/A | N/A |

*Appendix Table 1 Continued*

| **Publication, Country, Study Design** | **Sample Characteristics** Size Mean Age (SD) %Female Disease Status  Number of Chronic Conditions | **Frailty Characteristics** IADL/ADL, FAI, Barthel Index, GFI  Mean (SD)^1^ | **Mobility Characteristics** SPPB, TUG, 6MW   Mean (SD)^1^ | | **Global Cognition** MMSE, MoCA, GDS  Mean (SD)^1^ |
| --- | --- | --- | --- | --- | --- |
| Bleijenberg 2017, Netherlands, cluster-randomized controlled trial | Size 3092 - Intervention (Fraily Screening + GP Care): 790 - Intervention (Frailty Screening + Nurse-Led Care): 1446 - Control: 856 Age 73.5 (8.2) %Female 55.3% Disease Status Not reported  Chronic Conditions: Not reported | Intervention (Fraily Screening + GP Care: Multimorbidity as indicated by Frailty Index Score ≥0.20 (≥ 10/50 of the frailty deficits) Intervention (Frailty Screening + Nurse-Led Care): Multimorbidity as indicated by Frailty Index Score ≥0.20 (≥ 10/50 of the frailty deficits) & Groningen Frailty Indicator (GFI) score of ≥4 (total score ranges from 0 (not frail) to 15 (severely frail) Control: Multimorbidity as indicated by Frailty Index Score ≥0.20 (≥ 10/50 of the frailty deficits) | | N/A1 | N/A |

*Appendix Table 1 Continued*

| **Publication, Country, Study Design** | **Sample Characteristics** Size Mean Age (SD) %Female Disease Status  Number of Chronic Conditions | **Frailty Characteristics** IADL/ADL, FAI, Barthel Index, GFI  Mean (SD)^1^ | **Mobility Characteristics** SPPB, TUG, 6MW   Mean (SD)^1^ | | **Global Cognition** MMSE, MoCA, GDS  Mean (SD)^1^ |
| --- | --- | --- | --- | --- | --- |
| Boult 2001, USA, randomized clinical trial | Size 568 - Control: 274 - Intervention: 294 Age - Control: 78.7(5.8) - Intervention: 78.8 (5.3) %Female - Control: 41.6% - Intervention: 45.9% Disease Status Predominant history of coronary artery disease and arthritis among participants  Chronic Conditions: 66.9% with ≥1 chronic condition | N/A | | N/A | GDS ≥ 11 (%)  Intervention: 8.1%  Control: 12.5% |
| Braithwaite 2003, USA, computer prospective cohort simulation | Sample size not reported 80 80% All had hip fractures  Not reported | N/A | | N/A | N/A |
| Brinda 2014, India, cross-sectional | 85 74.3 (6.7) 65.9% Not reported  Not reported | all unable to perform at least one of the Activities of Daily Living | | N/A | N/A |

*Appendix Table 1 Continued*

| **Publication, Country, Study Design** | **Sample Characteristics** Size Mean Age (SD) %Female Disease Status  Number of Chronic Conditions | **Frailty Characteristics** IADL/ADL, FAI, Barthel Index, GFI  Mean (SD)^1^ | **Mobility Characteristics** SPPB, TUG, 6MW   Mean (SD)^1^ | | **Global Cognition** MMSE, MoCA, GDS  Mean (SD)^1^ |
| --- | --- | --- | --- | --- | --- |
| Challis 2002, England, quasi-experimental conrolled trial | Size 86 - Experimental: 43 - Control: 43 Age - Experimental: 80.4 - Control: 79.8 %Female - Experimental: 30.2% - Control: 30.2% Disease Status All subjects had dementia  Chronic Conditions  Not reported | N/A | | N/A | N/A |
| Challis 2004, England, randomized controlled trial | Size 256 - Experimental: 129 - Control: 127 Age - Experimental: 82 (7.2)  - Control: 82 (7.8) %Female - Experimental: 74% - Control: 72% Disease Status Most common chronic conditions among participants were arthritis and hypertension  Chronic Conditions  Not reported | Barthel Index: - Experimental: 78.1 (16.6) - Control: 76.6 (15.2) | | N/A | MMSE <24: Experimental: 67% Control: 54%  GDS >5:  Experimental: 39%  Control: 35% |

*Appendix Table 1 Continued*

| **Publication, Country, Study Design** | **Sample Characteristics** Size Mean Age (SD) %Female Disease Status  Number of Chronic Conditions | **Frailty Characteristics** IADL/ADL, FAI, Barthel Index, GFI  Mean (SD)^1^ | **Mobility Characteristics** SPPB, TUG, 6MW   Mean (SD)^1^ | | **Global Cognition** MMSE, MoCA, GDS  Mean (SD)^1^ |
| --- | --- | --- | --- | --- | --- |
| Chavan 2020, USA, observational study | 23695  74 (SD unreported)  58%  Cancer survivors  67.4% of cancer survivors with ≥2 chronic condition | ADL limitations (≥2): 12.9%  IADL limitations (≥2): 8.0% | | N/A | N/A |
| Chen 2000/2001, USA, cost-effectiveness study | Size (Patient characteristics by discharge location) Stroke: - Home: 160 - Home Care: 125  - Rehab: 79 Hip Procedure: - Home: 183 - Home Care: 81 Hip Fracture: - Home: 120 - Home Care: 115 - Rehab: 89 Age Mean age not reported in this study, patients were 65 and older. %Female: Not reported Disease Status: Not reported  Chronic Conditions: Not reported | Prior ADL/IADL (0-100): Stroke: - Home: 89.68 (15.96) - Home Care: 86.13 (19.48) - Rehab: 92.24 (10.18) Hip Procedure: - Home: 91.87 (11.97) - Home Care: 87.67 (14.01) Hip Fracture: - Home: 89.26 (16.66) - Home Care: 85.73 (18.76) - Rehab: 86.68 (16.19)  Discharge ADL/IADL (0-100): Stroke: - Home: 69.87 (24.89) - Home Care: 56.25 (24.80) - Rehab: 37.56 (15.33) Hip Procedure: - Home: 66.29 (16.74) - Home Care: 57.00 (16.82) Hip Fracture: - Home: 52.74 (17.84) - Home Care: 48.13 (20.13) - Rehab: 41.46 (16.55) | | N/A | N/A |

*Appendix Table 1 Continued*

| **Publication, Country, Study Design** | **Sample Characteristics** Size Mean Age (SD) %Female Disease Status  Number of Chronic Conditions | **Frailty Characteristics** IADL/ADL, FAI, Barthel Index, GFI  Mean (SD)^1^ | **Mobility Characteristics** SPPB, TUG, 6MW   Mean (SD)^1^ | | **Global Cognition** MMSE, MoCA, GDS  Mean (SD)^1^ |
| --- | --- | --- | --- | --- | --- |
| Chiatti 2015, Italy, cost-of-illness study based on randomized controlled trial | 438 Age, Mean (SE): 81.5 (5.7) 62.1% Alzheimer's  Not reported | IADL, Mean (SE): 35.1 (13.4)  ADL, Mean(SE): 1.5 (1.6) | | N/A | MMSE, Mean (SE): 16 (3) |
| Chiu 2000, Taiwan, cost-effectiveness analysis | Size - Hospital: 106 - Home Care: 60 - Family Care: 87 Age - Hospital: 72.5 (3.15) - Home Care: 73.5 (3.87) - Family Care: 69.8 (2.01) %Female - Hospital: 35.8% - Home Care: 33.3% - Family Care: 34.5% Disease Status All participants were hospitalized stroke patients with severe physical disabilities  Chronic Conditions  Not reported | ADL Scores at Discharge:  - Hospital: 17.9 (4.67) - Home Care: 17.3 (4.96) - Family Care: 17.5 (3.94) | | N/A | N/A |

*Appendix Table 1 Continued*

| **Publication, Country, Study Design** | **Sample Characteristics** Size Mean Age (SD) %Female Disease Status  Number of Chronic Conditions | **Frailty Characteristics** IADL/ADL, FAI, Barthel Index, GFI  Mean (SD)^1^ | **Mobility Characteristics** SPPB, TUG, 6MW   Mean (SD)^1^ | | **Global Cognition** MMSE, MoCA, GDS  Mean (SD)^1^ |
| --- | --- | --- | --- | --- | --- |
| Coleman 1999, USA, randomized controlled trial | Size 170 - Intervention: 96 - Control: 73 Age: - Intervention: 77.3 - Control: 77.4 %Female - Intervention: 47.9% - Control: 49.3% Disease Status Heart failure  Chronic Conditions  Mean Chronic Disease Score = 7.7 | N/A | | N/A | N/A |
| Colon-Emeric 2020, USA, observational study | 5634  77.4 (17.37)  56%  Not reported  Mean Charlson Comorbidity Index: 2.33 | 17% of participants had >3 ADL disabilities before hospitalization  15% of participants had >3 IADL disabilities before hospitalization | | SPPB score before hospitalization: 6.32 (10.18) | N/A |
| Davis 2011, Canada, randomized controlled trial | 123 69.4 (2.8) 100% N/A  Not reported | N/A | | TUG: 6.6 (1.4) | MMSE: 28.6 (1.4)  GDS: 0.5 (20.0) |
| Deardorff 2019, USA, retrospective analysis | **With Dementia:** 871 81.9 (7.0) 63.7% Dementia Comorbidity score, mean (SD): 3.2 (1.8) **Without Dementia:** 23138 76.4 (7.0) 57.9% No Dementia  Comorbidity score, mean (SD): 2.7 (2.7) | N/A | | N/A | N/A |

*Appendix Table 1 Continued*

| **Publication, Country, Study Design** | **Sample Characteristics** Size Mean Age (SD) %Female Disease Status  Number of Chronic Conditions | **Frailty Characteristics** IADL/ADL, FAI, Barthel Index, GFI  Mean (SD)^1^ | **Mobility Characteristics** SPPB, TUG, 6MW   Mean (SD)^1^ | | **Global Cognition** MMSE, MoCA, GDS  Mean (SD)^1^ |
| --- | --- | --- | --- | --- | --- |
| Deb 2018, USA, retrospective cross-sectional | **Alzheimer’s:** 662 65-75 years: 15.4% 75 and older: 84.6% 66.3% Alzheimer’s and related dementias  % with >3 Chronic Conditions: 37.5%  **No Alzheimer’s:** 13,398 65-75 years: 56.7% 75 and older: 43.3% 56.4% N/A  % with >3 Chronic Conditions: 25.1% | N/A | | N/A | N/A |
| Del Sindaco, 2007, Italy, randomized open trial | Size 173 - Intervention: 86 - Control: 87 Age - Intervention: 77.4 (5.9) - Control: 77.5 (5.7) %Female - Intervention: 51.2% - Control:52.8% Disease Status Heart failure  Number of Chronic Conditions  Charlson Comorbidity Index, Mean (SE):  - Intervention: 2.36 (1.56)  - Control: 2.27 (1.54) | Full sample not reported  IADL Disability - Intervention: 55.8% had IADL disability (impairment in at least 2 IADL or 1 BADL) - Control: 56.3% had IADL disability | | N/A | N/A |

*Appendix Table 1 Continued*

| **Publication, Country, Study Design** | **Sample Characteristics** Size Mean Age (SD) %Female Disease Status  Number of Chronic Conditions | **Frailty Characteristics** IADL/ADL, FAI, Barthel Index, GFI  Mean (SD)^1^ | **Mobility Characteristics** SPPB, TUG, 6MW   Mean (SD)^1^ | | **Global Cognition** MMSE, MoCA, GDS  Mean (SD)^1^ |
| --- | --- | --- | --- | --- | --- |
| Dodel 2015, France, ermany, UK, observational study | 1497 77.6 (7.7) 55% Alzheimer’s disease and caregivers  Number of chronic conditions, Mean (SD): 1.4 (1.2) | N/A | | N/A | MMSE: 17.4 (6.3) - Mild MMSE Severity (21-26) - Moderate MMSE Severity (15-20) - Moderately Severe/Severe MMSE Severity (0-15) |
| Eamer 2018, Canada, cohort observational study | 150 75.5 (7.6) 45.9% Not reported  Number of chronic conditions, Mean (SD): 1.0 (1.0) | no preoperative dependence in 3 or more activities of daily living | | N/A | N/A |
| Eamer 2019, Canada, prospective cohort cost analysis | 321 76.1 (7.7) 45% All participants underwent emergency general surgery  Number of chronic conditions, Mean (SD): 1.6 (1.8) | N/A | | N/A | N/A |
| Ensrud 2018, USA, prospective cohort study | 2150 80.2 (4.4) 100% Not reported  % with ≥4 chronic conditions: 23.8% | IADL Impairments: 1.1 (1.5) | | N/A | MMSE: 23.8 (2.4)  GDS: 2.4 (2.8) |

*Appendix Table 1 Continued*

| **Publication, Country, Study Design** | **Sample Characteristics** Size Mean Age (SD) %Female Disease Status  Number of Chronic Conditions | **Frailty Characteristics** IADL/ADL, FAI, Barthel Index, GFI  Mean (SD)^1^ | **Mobility Characteristics** SPPB, TUG, 6MW   Mean (SD)^1^ | | **Global Cognition** MMSE, MoCA, GDS  Mean (SD)^1^ |
| --- | --- | --- | --- | --- | --- |
| Fairhall 2015, Australia, cost-effectiveness study embedded within a randomized controlled trial | Size 241 - Intervention: 120 - Control: 121 Age, Mean (SD; Range) - Intervention: 83.4 (5.81; 71–99) - Control: 83.2 (5.91; 71–101) %Female - Intervention: 67% - Control: 68% Disease Status: Not reported  Number of Chronic Conditions, Mean (SD;Range)  - Intervention: 7.44 (2.90; 0-13)  - Control: 7.37 (2.58; 0-12) | Barthel Index, Mean (SD; Range): - Intervention: 93.9 (11.1; 45–100) - Control: 92.5 (14.3; 2–100) | | SPPB, Mean (SD; Range): - Intervention: 5.2 (1.89; 0–11) - Control: 5.74 (2.12; 0–12) | MMSE, Mean (SD; Range): - Intervention: 26.6 (2.58; 19–30) - Control: 25.9 (3.14; 18–30)  GDS, Mean (SD; Range):  - Intervention: 4.76(3.18;0-14)  - Control: 5.06 (3.19; 0-14) |
| Farre 2016, Spain, cross-sectional study | 174 82.5 (7.3) 66.1% All participants had dementia  Charlson Comorbidity Index, Mean (SD): 1.9 (1.3) | Dependency in ADL (Katz Index): 2.9 (2.0) - Functional Independence: 31.6% - Partial Disability: 38.5% - Total Disability: 29.9% | | N/A | MMSE: 16.1 (5.4) - Mild: 19.8% - Moderate: 45.6% - Moderately severe/severe: 34.6% |
| Forster 2009, UK, randomized controlled trial | Size 265 - Intervention: 132 - Control: 133 Age, Median (IQR): - Intervention: 77 (70–83) - Control: 79 (70–84) %Female - Intervention: 57% - Control: 52% Disease Status: Stroke  Number of Chronic Conditions: Not reported | Full sample not reported  Barthel, Median (IQR): - Intervention: 16 (11–18) - Control: 15 (9–18) | | N/A | N/A |

*Appendix Table 1 Continued*

| **Publication, Country, Study Design** | **Sample Characteristics** Size Mean Age (SD) %Female Disease Status  Number of Chronic Conditions | **Frailty Characteristics** IADL/ADL, FAI, Barthel Index, GFI  Mean (SD)^1^ | **Mobility Characteristics** SPPB, TUG, 6MW   Mean (SD)^1^ | | **Global Cognition** MMSE, MoCA, GDS  Mean (SD)^1^ |
| --- | --- | --- | --- | --- | --- |
| Graff 2008, Netherlands, single-blind randomised controlled trial | Full sample not reported  Size 135 - Intervention: 68 - Control: 67 Age - Intervention: 79.1 (6.2) - Control: 77.1 (6.3) %Female - Intervention: 57.4% - Control: 53.7% Disease Status All subjects had dementia  Number of Chronic Conditions  Cumulative Illness Comorbidity Index:  - Intervention: 10.7 (3.5)  - Control: 11.6 (4.3) | N/A | | N/A | MMSE: - Intervention: 19.0 (5.7) - Control: 19.0 (4.0)  GDS:  - Intervention: 6.9 (3.0)  - Control; 7.5 (3.0) |
| Gustavsson 2010, 12 European Countries, prospective observational study | 1381 76.3 65% Alzheimer's disease  Not reported | N/A | | N/A | 20.4 (10-26) |

*Appendix Table 1 Continued*

| **Publication, Country, Study Design** | **Sample Characteristics** Size Mean Age (SD) %Female Disease Status  Number of Chronic Conditions | **Frailty Characteristics** IADL/ADL, FAI, Barthel Index, GFI  Mean (SD)^1^ | **Mobility Characteristics** SPPB, TUG, 6MW   Mean (SD)^1^ | | **Global Cognition** MMSE, MoCA, GDS  Mean (SD)^1^ |
| --- | --- | --- | --- | --- | --- |
| Han 2019, UK, retrospective longitudinal | Full sample not reported  Size 566101 - Non-frail: 245,294 - Mild frailty: 216,354  - Moderate frailty: 82,187 - Severe frailty: 22,266 Age: - Non-frail: 73.02 (6.47) - Mild frailty: 76.23 (7.25) - Moderate frailty: 79.64 (7.38) - Severe frailty: 82.44 (7.01) %Female - Non-frail: 52.25% - Mild frailty: 56.92% - Moderate frailty: 63.30% - Severe frailty: 70.04% Disease Status Heart failure  Number of Chronic Conditions  Not reported | N/A | | N/A | N/A |
| Hardy 2010, USA, analysis of longitudinal data | 5895 Age, Mean (SE): 75.5 (0.1) 57% Not reported  % with ≥1 Chronic Condition: 68% | IADL limitations: - None: 65% - 1-2: 23% - 3 or more: 11% | | N/A | N/A |

*Appendix Table 1 Continued*

| **Publication, Country, Study Design** | **Sample Characteristics** Size Mean Age (SD) %Female Disease Status  Number of Chronic Conditions | **Frailty Characteristics** IADL/ADL, FAI, Barthel Index, GFI  Mean (SD)^1^ | **Mobility Characteristics** SPPB, TUG, 6MW   Mean (SD)^1^ | **Global Cognition** MMSE, MoCA, GDS  Mean (SD)^1^ |
| --- | --- | --- | --- | --- |
| Harrow 2004, USA, obervational study | 1207 78.7 (8.2) 55.4% Alzheimer's  Not reported | N/A | N/A | MMSE: 12.6 |
| Hay 2002, USA, randomized controlled trial | Size 163 - Intervention: 51 - Combined Control: 112 Age Intervention:  - <70: 26% - 70-79: 47% - ≥80: 27% Combined Control: - <70:23% - 70-79: 52% - ≥80: 25% %Female - Intervention: 71% - Combined Control: 71% Disease Status Not reported  Number of Chronic Conditions  Not reported | N/A | N/A | MMSE:  Intervention: - MMSE ≤ 23: 10% - MMSE > 23: 90% Combined Control: - Impaired: 17% - Unimpaired: 83%  GDS:  Intervention:  - GDS ≤ 5: 82%  - GDS > 5: 18%  Combined Control:  - GDS ≤ 5: 76%  - GDS > 5: 24% |

*Appendix Table 1 Continued*

| **Publication, Country, Study Design** | **Sample Characteristics** Size Mean Age (SD) %Female Disease Status  Number of Chronic Conditions | **Frailty Characteristics** IADL/ADL, FAI, Barthel Index, GFI  Mean (SD)^1^ | **Mobility Characteristics** SPPB, TUG, 6MW   Mean (SD)^1^ | **Global Cognition** MMSE, MoCA, GDS  Mean (SD)^1^ |
| --- | --- | --- | --- | --- |
| Hektoen 2016, Norway, further exploration of costs of randomized controlled trial, used 1st year after hip fracture for all participants | 396 83.3 73.7% Not reported  Not reported | Nottingham Extended ADL Scale: 42.2 (17.6) | N/A | N/A |
| Hendriks 2008, Netherlands, randomized controlled trial | Full sample not reported  Size 333 - Intervention: 166 - Control: 167 Age - Intervention: 74.5 (5.9) - Control: 75.2 (6.9) %Female - Intervention: 66.9% - Control: 70.1% Disease Status  N/A  Number of Chronic Conditions Number of illnesses, Mean (SD): - Intervention: 2.8 (2.0) - Control: 3.2 (2.5) | FAI:  Intervention: - At Baseline: 23.2 (8.7) - At 12 Months: 25.6 (7.9) Control: - At Baseline: 23.7 (8.6) - At 12 Months: 24.5 (9.1) | N/A | N/A |

*Appendix Table 1 Continued*

| **Publication, Country, Study Design** | **Sample Characteristics** Size Mean Age (SD) %Female Disease Status  Number of Chronic Conditions | **Frailty Characteristics** IADL/ADL, FAI, Barthel Index, GFI  Mean (SD)^1^ | **Mobility Characteristics** SPPB, TUG, 6MW   Mean (SD)^1^ | **Global Cognition** MMSE, MoCA, GDS  Mean (SD)^1^ |
| --- | --- | --- | --- | --- |
| Hughes 2000, USA, multi-site randomized controlled trial | Size 1966 - Intervention: 981 - Control: 985 Age - Intervention: 70.4 (10.3) - Control: 70.4 (10.3) %Female - Intervention: 96.5% - Control: 96.2% Disease Status Terminally Ill: - Intervention: 20.7% - Control: 20.1% Not Terminally Ill: Severely Disabled: - Intervention: 75.1% - Control: 74.4%  Number of chronic conditions  % of participants with high Smith Comorbidity Index:  - Intervention: 33.6%  - Control: 37.5% | Katz ADL: - Intervention: 3.2 (1.8) - Control: 3.2 (1.8) | N/A | N/A |
| Hui 1995, Hong Kong, randomized controlled trial | Size 120 - Intervention: 59 - Control: 61 Age: - Intervention: 73.1 (5.42) - Control: 74.1 (5.89) %Female - Intervention: 58% - Control: 54% Disease Status Stroke  Number of chronic conditions  Not reported | Barthel: - Intervention: 9.9 (4.9) - Control: 10.4 (5.3) | N/A | N/A |

*Appendix Table 1 Continued*

| **Publication, Country, Study Design** | **Sample Characteristics** Size Mean Age (SD) %Female Disease Status  Number of Chronic Conditions | **Frailty Characteristics** IADL/ADL, FAI, Barthel Index, GFI  Mean (SD)^1^ | **Mobility Characteristics** SPPB, TUG, 6MW   Mean (SD)^1^ | **Global Cognition** MMSE, MoCA, GDS  Mean (SD)^1^ |
| --- | --- | --- | --- | --- |
| Joling 2015, Netherlands, prospective cohort | 192 74.7 (8.9) 32.3% Alzheimer's: 57.3%  Mean Number of Chronic Conditions: 1.6 (1.4) | IADL: 2.6 (1.6) | N/A | MMSE: 21.6 (5.3) |
| Kehusmaa 2013, Finland, multilevel regression model | 732 78 86% Not reported  Not reported | 61% had limited functioning capacity 22% had poor IADL skills | N/A | MMSE: 29% had MMSE <24  GDS: 10% had GDS >7 |
| Komisar 1997, USA, observational study | 397 Not reported Not reported Not reported  Not reported | ADL: 3 or more limitations | N/A | N/A |
| Kramer 1997, USA, prospective cohort study | Full sample not reported Size - Hip Fracture Patients: 183 - Stroke Patients: 292 Age - Hip Fracture Patients: 80.9 - Stroke Patients: 75.8 %Female - Hip Fracture Patients: 80% - Stroke Patients: 58% Disease Status Not reported  Number of Chronic Conditions  Not reported | **At Admission to Rehabilitation:** Barthel Index:  - Hip Fracture Patients: 10.8 - Stroke Patients: 8.2 No. of ADL Dependencies:  - Hip Fracture Patients: 3.6 - Stroke Patients: 4.3 | N/A | **At Admission to Rehabilitation:** MMSE, mean: - Hip Fracture Patients: 22.8 - Stroke Patients: 19.8  GDS score indicative of depression:  - Hip Fracture Patients: 26  -Stroke Patients: 37 |

*Appendix Table 1 Continued*

| **Publication, Country, Study Design** | **Sample Characteristics** Size Mean Age (SD) %Female Disease Status  Number of Chronic Conditions | **Frailty Characteristics** IADL/ADL, FAI, Barthel Index, GFI  Mean (SD)^1^ | **Mobility Characteristics** SPPB, TUG, 6MW   Mean (SD)^1^ | **Global Cognition** MMSE, MoCA, GDS  Mean (SD)^1^ |
| --- | --- | --- | --- | --- |
| Kronborg 2006, 4 Danish counties, randomized controlled trial | Full sample not reported  Size 2092 Age: - 75 y/o - 80 y/o %Female - 75 y/o: 54% - 80 y/o: 59% Disease Status Not reported  Number of Chronic Conditions  Not reported | % Independent of help from others: 6 activities - 75 y/o: 82% - 80 y/o: 71% 5 activities - 75 y/o: 9% - 80 y/o: 12% 4 activities - 75 y/o: 4% - 80 y/o: 8% 3 activities: - 75 y/o: 2% - 80 y/o: 3% 2 activities - 75 y/o: 2% - 80 y/o: 3% 1 activity - 75 y/o: 1% - 80 y/o: 2% 0 activities - 75 y/o: 1% - 80 y/o: 1% | N/A | N/A |
| Ku 2019, Taiwan, linear mixed mode | 161 80 59.6% Dementia  Not reported | ADL: 75.8 | N/A | % MMSE score ≤ 8: 17.7% |

*Appendix Table 1 Continued*

| **Publication, Country, Study Design** | **Sample Characteristics** Size Mean Age (SD) %Female Disease Status  Number of Chronic Conditions | **Frailty Characteristics** IADL/ADL, FAI, Barthel Index, GFI  Mean (SD)^1^ | **Mobility Characteristics** SPPB, TUG, 6MW   Mean (SD)^1^ | **Global Cognition** MMSE, MoCA, GDS  Mean (SD)^1^ |
| --- | --- | --- | --- | --- |
| Lafortune 2020, Canada, secondary analysis of data collected during the randomized trial | 1164 82.2 70.9% Not reported  ≥ 3 chronic conditions: 38.9% | Difference with 0–2 activities: 35.6% Difference with 3–4 activities: 26.6% Difference with 4+ activities: 37.8% | N/A | N/A |
| Langa 2004, USA, comparative study | Size 6649 - no depressive symptoms: 2,531 - 1-3 depressive symptom: 2,954 - 4-8 depressive symptoms: 1,164 Age - no depressive symptoms: 76.3 (0.3) - 1-3 depressive symptom: 77.6 (0.3) - 4-8 depressive symptoms: 78.3 (0.4) %Female - no depressive symptoms: 58% - 1-3 depressive symptom: 65% - 4-8 depressive symptoms:73%  Disease Status Not reported  Number of Chronic Conditions  50.3% with ≥1 Chronic Condition | **Number of impaired IADL, % of sample:** 0 - no depressive symptoms: 86 - 1-3 depressive symptom:73 - 4-8 depressive symptoms: 54 1-3 - no depressive symptoms: 13 - 1-3 depressive symptom:26 - 4-8 depressive symptoms: 43 4-5 - no depressive symptoms: 1 - 1-3 depressive symptom 1 - 4-8 depressive symptoms:3 | N/A | N/A |
| Leeuwan 2015, Netherlands, economic evaluation alongside randomized controlled trial | 1147 80.5 (7.5) 66.5% Not reported  Not reported | IADL limitations: 2.6 (1.6)  ADL limitations: 0.9 (1.2) | N/A | N/A |

*Appendix Table 1 Continued*

| **Publication, Country, Study Design** | **Sample Characteristics** Size Mean Age (SD) %Female Disease Status  Number of Chronic Conditions | **Frailty Characteristics** IADL/ADL, FAI, Barthel Index, GFI  Mean (SD)^1^ | **Mobility Characteristics** SPPB, TUG, 6MW   Mean (SD)^1^ | **Global Cognition** MMSE, MoCA, GDS  Mean (SD)^1^ |
| --- | --- | --- | --- | --- |
| Lewin 2013, Australia, randomized controlled trial | Size 1501 - Intention to treat - Control: 375 - Intention to treat - Intervention: 375 - Actual treatment - Control: 375 - Actual treatment - Intervention: 376 Age - Intention to treat - Control: 82.7 (7.7) - Intention to treat - Intervention: 81.8 (7.2) - Actual treatment - Control: 82.7 (7.6) - Actual treatment - Intervention: 81.9 (7.4) %Female - Intention to treat - Control: 64.5% - Intention to treat - Intervention: 70.1% - Actual treatment - Control: 64.3% - Actual treatment - Intervention: 72.3%  Disease Status  Not reported  Number of Chronic Conditions  Not reported | Full sample not reported  IADL Silver Chain Score: 1501 - Intention to treat - Control: 7.2 (3.6) - Intention to treat - Intervention: 8.1 (3.2) - Actual treatment - Control: 7.2 (3.7) - Actual treatment - Intervention: 8.2 (3.1) | N/A | N/A |

*Appendix Table 1 Continued*

| **Publication, Country, Study Design** | **Sample Characteristics** Size Mean Age (SD) %Female Disease Status  Number of Chronic Conditions | **Frailty Characteristics** IADL/ADL, FAI, Barthel Index, GFI  Mean (SD)^1^ | **Mobility Characteristics** SPPB, TUG, 6MW   Mean (SD)^1^ | **Global Cognition** MMSE, MoCA, GDS  Mean (SD)^1^ |
| --- | --- | --- | --- | --- |
| Liotta 2019, Italy, part of a longitudinal study | Size Pre-Frail: 459 Frail: 175 Very Frail: 97 Age Pre-Frail: 76.95 (6.33) Frail: 79.68 (7.51) Very Frail: 83.72 (6.94) %Female Pre-Frail: 55% Frail: 69.1% Very Frail: 67% Disease Status  Not reported  Number of Chronic Conditions  Chronic conditions reported by the participant’s GP, Mean (SD): 4.1 (2.3) | N/A | N/A | N/A |
| Lu 2020, China, observational study | Size  Non-depressed: 838  Mild Depression: 1403  Moderate Depression: 367  Moderate-Severe Depression: 99  Age  Non-depressed: 76.9 (8.3)  Mild Depression: 76.6 (8.4)  Moderate Depression: 75.7 (8.0)  Moderate-Severe Depression: 74 (9.1)  %Female  Non-depressed: 76%  Mild Depression: 79%  Moderate Depression: 81%  Moderate-Severe Depression: 75%  Disease Status  Depression  Number of Chronic Conditions  % with ≥ Chronic Conditions: 11.1% | N/A | N/A | MoCA: 21.6 (4.4) |

*Appendix Table 1 Continued*

| **Publication, Country, Study Design** | **Sample Characteristics** Size Mean Age (SD) %Female Disease Status  Number of Chronic Conditions | **Frailty Characteristics** IADL/ADL, FAI, Barthel Index, GFI  Mean (SD)^1^ | **Mobility Characteristics** SPPB, TUG, 6MW   Mean (SD)^1^ | | **Global Cognition** MMSE, MoCA, GDS  Mean (SD)^1^ |
| --- | --- | --- | --- | --- | --- |
| Mann 1999, USA, randomized controlled trial | Size 104 - Intervention: 52 - Control: 52 Age 73.0 (8.4) - Intervention: 74.3 (7.7) - Control: 71.6 (8.9) %Female 70.2% - Intervention: 65.4% - Control: 75.0% Disease Status Not reported  Number of Chronic Conditions  6.5 (2.9) - Intervention: 6.2 (2.7) - Control: 7.1 (3.3) | IADL Total - Initial: - Intervention: 9.6 (3.1) - Control: 9.2 (3.1) | | N/A | MMSE - Initial:  - Intervention: 28.8 (1.7) - Control: 28.3 (1.8) |
| Marshall 1999, USA, randomized controlled trial | Size 280 - Intervention: 140 - Control: 140 Age: - Intervention: 81 - Control: 82 %Female - Intervention: 65% - Control: 71% Disease Status Not reported  Number of Chronic Conditions  Not reported | IADL score **Intervention**: - base: 5.65 - year 1: 5.68  - year 2: 5.57 **Control**: - base: 5.68  - year 1: 5.83  - year 2: 6.06 | | N/A | N/A |

*Appendix Table 1 Continued*

| **Publication, Country, Study Design** | **Sample Characteristics** Size Mean Age (SD) %Female Disease Status  Number of Chronic Conditions | **Frailty Characteristics** IADL/ADL, FAI, Barthel Index, GFI  Mean (SD)^1^ | **Mobility Characteristics** SPPB, TUG, 6MW   Mean (SD)^1^ | **Global Cognition** MMSE, MoCA, GDS  Mean (SD)^1^ |
| --- | --- | --- | --- | --- |
| Maru 2015, Australia, randomized controlled trial | 280 71 27% Chronic Heart Failure  Charlson Comorbidity Index, Mean (SD): 6.2 (2.4) | N/A | N/A | N/A |
| Max 1995, USA, observational study | 93 74.7 (8.9) 59.1% Alzheimer's Disease  Not reported | N/A | N/A | 6.5 (6.6) |
| McCusker 2003, Canada, quasi-randomized controlled trial | Size 388 - Intervention: 178 - Control: 210 Age - Intervention: 76.7 (7.1) - Control: 76.5 (7.0) %Female - Intervention: 55.6% - Control: 65.2% Disease Status Not reported  Number of Chronic Conditions  Comorbidity Index:  - Intervention: 2.4 (2.2)  - Control: 2.3 (1.9) | Disability at baseline was a 4-category variable derived from ADL:   Disability (premorbid): Intervention: - None-Mild: 52.3% - Moderate-Severe: 47.7% Control: - None-Mild: 52.4% - Moderate-Severe: 47.6% | N/A | N/A |
| McNamee 1999, England, prospective/longitudinal randomized study | 1055 80 (quartiles 75-85 years) 71% Not reported  Not reported | All participants were classified as frail older people  70% of participants were physically frail determined by ADL 11% were both mentally and physically frail | N/A | 19% were mentally frail determined by the MMSE |

*Appendix Table 1 Continued*

| **Publication, Country, Study Design** | **Sample Characteristics** Size Mean Age (SD) %Female Disease Status  Number of Chronic Conditions | **Frailty Characteristics** IADL/ADL, FAI, Barthel Index, GFI  Mean (SD)^1^ | **Mobility Characteristics** SPPB, TUG, 6MW   Mean (SD)^1^ | **Global Cognition** MMSE, MoCA, GDS  Mean (SD)^1^ |
| --- | --- | --- | --- | --- |
| Melin 1993, Sweden, randomized controlled trial | 173 80.5 72% Not reported  51.5% had ≥ 1 chronic condition | Personal ADL according to Katz index: 3.5  Instrumental ADL: 6 | N/A | MMSE, Mean (SE): - Treatment: 22.7 (0.4) - Control: 22.3 (0.5) |
| Metzelthin 2015, Netherlands, cost-effectiveness study alongside a cluster randomized controlled trial | Full sample not reported  Size 346 - Intervention: 193 - Control: 153 Age - Intervention: 77.5 (5.3) - Control: 76.8 (4.9) %Female - Intervention: 55% - Control: 60% Disease Status Not reported  Number of Chronic Conditions  Not reported | GFI: - Intervention: 7.1 (1.9) - Control: 6.7 (1.7) | N/A | N/A |
| Michalowsky 2016, Germany, cross-sectional | 262 80.69 (5.4) 56.5% Dementia  Number of ICD-10 Diagnoses, Mean (SD): 13.4 (7.4) | B-ADL: 4.19 (2.5) | N/A | MMSE: 21.66 (5.0)  GDS: 3.34 (2.4) |
| Miller 2005, England, randomized controlled trial | Size at 12-months: 182 - Intervention: 132 - Control: 140 Median Age: 80 67% All participants were elderly admitted acutely to the hospital  Not reported | N/A | N/A | N/A |

*Appendix Table 1 Continued*

| **Publication, Country, Study Design** | **Sample Characteristics** Size Mean Age (SD) %Female Disease Status  Number of Chronic Conditions | **Frailty Characteristics** IADL/ADL, FAI, Barthel Index, GFI  Mean (SD)^1^ | **Mobility Characteristics** SPPB, TUG, 6MW   Mean (SD)^1^ | **Global Cognition** MMSE, MoCA, GDS  Mean (SD)^1^ |
| --- | --- | --- | --- | --- |
| Mintzer 1997, USA, quasi-experimental study | Size Intervention: 68 Control: 112 Age Intervention: 78.44 (6.7) Control: 78.1 (7.2) %Female Intervention: 62% Control: 64% Disease Status Dementia  Number of Chronic Conditions  Not reported | N/A | N/A | MMSE: Intervention: 12.7 (7.48) Control: 11.8 (7.38) |
| Mitchell 2019, Australia, retrospective analysis | Size 19,499 Age: 65-69: 6.2% 70-79: 84.6% 80-89: 50.9% 90+: 17.2% %Female 71.8% Disease Status Not reported  Number of Chronic Conditions  % ≥1 on Charlson Comorbidities Scale: 36.6% | N/A | All had fall-related hip fracture and hospitalized | N/A |
| Murray 2003, USA, economic analysis | 277 85 56% Not reported  Not reported | IADL (based on HARP tool): 1.35 - Independent in 2-7 activities (2 points): 67.5% - Dependent in 0-1 activities (0 points): 32.5% | N/A | MMSE - short term (based on HARP tool): 0.35 - 15-21 (0 points): 65% - 0-14 (1 points): 35% |

*Appendix Table 1 Continued*

| **Publication, Country, Study Design** | **Sample Characteristics** Size Mean Age (SD) %Female Disease Status  Number of Chronic Conditions | **Frailty Characteristics** IADL/ADL, FAI, Barthel Index, GFI  Mean (SD)^1^ | **Mobility Characteristics** SPPB, TUG, 6MW   Mean (SD)^1^ | **Global Cognition** MMSE, MoCA, GDS  Mean (SD)^1^ |
| --- | --- | --- | --- | --- |
| Nikolaus 1999, Germany, randomized controlled trial | Size 545 *At hospital discharge:* - Intervention: 181 - Assessment: 179 - Control: 185 *At 12-month after admission to study:* - Intervention: 140 - Assessment: 139 - Control: 141 Age 81.4 %Female 73.4% Disease Status  Not reported  Number of Chronic Conditions  Not reported | Barthel Score: All: 71.0  Functional Status at Hospital Discharge: Intervention - Basic ADL: 91.8 (89.7–93.9) - IADL: 5.7 (5.4–6.0) Assessment - Basic ADL: 92.6 (90.5–94.7) - IADL: 5.5 (5.3–5.7) Control - Basic ADL: 91.1 (89.3–93.9) - IADL: 5.5 (5.3–5.7) | N/A | N/A |
| Pinedo‑Villanueva 2019, UK, used data from cohort study | Size 442 - Without muscle weakness:393 - W/ muscle weakness: 49 Age, Median (lower quartile, upper quartile): - Without muscle weakness:75.2 (73.3, 77.7) - W/ muscle weakness: 76.6 (74.3, 78.7) %Female - Without muscle weakness: 48.9% - W/ muscle weakness: 59.2% Disease Status Not reported  Chronic Conditions  % with ≥1 Chronic Condition: 82.3% | Townsend disability*, Median (lower quartile, upper quartile) - Without muscle weakness:2.0 (0.0, 4.0) - W/ muscle weakness: 5.0 (2.0, 8.0)  **Townsend disability scale (14) is a score to reflect the difficulty in performing activities of daily living* | N/A | N/A |

*Appendix Table 1 Continued*

| **Publication, Country, Study Design** | **Sample Characteristics** Size Mean Age (SD) %Female Disease Status  Number of Chronic Conditions | **Frailty Characteristics** IADL/ADL, FAI, Barthel Index, GFI  Mean (SD)^1^ | **Mobility Characteristics** SPPB, TUG, 6MW   Mean (SD)^1^ | **Global Cognition** MMSE, MoCA, GDS  Mean (SD)^1^ |
| --- | --- | --- | --- | --- |
| Pitkala 2013, Finland, randomized controlled trial | Size 70 patient-caregiver dyads in each group (210 total) Age - Intervention 1: 77.7 (5.4) - Intervention 2: 78.3 (5.1) - Control: 78.1 (5.3) %Female - Intervention 1: 42.9% - Intervention 2: 35.7% - Control: 37.1% Disease Status Alzheimer’s Disease  Number of Chronic Conditions  Charlson Comorbidity Index:  - Intervention 1: 2.6 (1.8)  - Intervention 2: 2.5 (1.8)  - Control: 3.0 (1.7) | N/A | **SPPB**: Intervention 1: 9.8 (2.2) Intervention 2: 9.3 (2.4) Control: 9.7 (2.1) | **MMSE**: Intervention 1: 17.8 (6.6) Intervention 2: 18.5 (6.3) Control: 17.7 (6.2) |
| Reuben 2004, USA, longitudinal study | 4464 78.4 65.1% Not reported  Not reported | N/A | N/A | N/A |
| Rigaud 2003, France, retrospective cross sectional | Size 50 Age: - Males: 80.2 ± 4.4 - Females: 80.9 ± 6.3 %Female 78% Disease Status Alzheimer's Disease  Number of Chronic Conditions  Not reported | N/A | N/A | N/A |

*Appendix Table 1 Continued*

| **Publication, Country, Study Design** | **Sample Characteristics** Size Mean Age (SD) %Female Disease Status  Number of Chronic Conditions | **Frailty Characteristics** IADL/ADL, FAI, Barthel Index, GFI  Mean (SD)^1^ | **Mobility Characteristics** SPPB, TUG, 6MW   Mean (SD)^1^ | **Global Cognition** MMSE, MoCA, GDS  Mean (SD)^1^ |
| --- | --- | --- | --- | --- |
| Rojas 2010, Argentina, retrospective, observational and cross-sectional study | Size - Healthy Subjects: 29 - Dementia of Alzheimer Type (DAT): 44 - Frontotemporal Dementia (FTD): 34 - Vascular Dementia (VaD): 26 Age - Healthy Subjects: 68.0 (7.8) - DAT: 69.7 (5.1) - FTD: 68.7 (7.3) - VaD: 67.6 (7.9) %Female - Healthy Subjects: 65.5% - DAT: 54.5% - FTD: 52.9% - VaD: 50% Disease Status Dementia  Number of Chronic Conditions  Not reported | N/A | N/A | MMSE: - Healthy Subjects: 28.1 (2.1) - DAT: 18.6 (5.1) - FTD: 19.7 (9.7) - VaD: 20.6 (8.0) |
| Ruchlin 2001, USA, randomized controlled trial | Size 114 - Intervention: 59 - Control: 55 Age - Intervention: 79.2 (7.4)  - Control: 79.0 (7.8) %Female - Intervention: 74.6% - Control: 85.5% Disease Status Hip Fracture  Number of Chronic Conditions  Not reported | N/A | N/A | N/A |

*Appendix Table 1 Continued*

| **Publication, Country, Study Design** | **Sample Characteristics** Size Mean Age (SD) %Female Disease Status  Number of Chronic Conditions | **Frailty Characteristics** IADL/ADL, FAI, Barthel Index, GFI  Mean (SD)^1^ | **Mobility Characteristics** SPPB, TUG, 6MW   Mean (SD)^1^ | **Global Cognition** MMSE, MoCA, GDS  Mean (SD)^1^ |
| --- | --- | --- | --- | --- |
| Ruikes 2018, Netherlands, economic evaluation from a health care perspective performed alongside a cluster-controlled effectiveness study | Size 536 - Intervention: 287 - Control: 249 Age: - Intervention: 83.1 (5.6) - Control: 80.5 (6.0) %Female - Intervention: 66.9% - Control: 64.3% Disease Status Not reported  Number of Chronic Conditions  Not reported | N/A | N/A | MMSE:  - Intervention: 7.5 (7.0) - Control: 5.3 (4.8) |
| Schnaider-Beeri 2002a, Israel, qualitative prospective study | Size AD Patients: 71 Primary Caregivers: 71 Age AD Patients: 76.4 (7.5) Primary Caregivers: 61.2 (12.8) %Female AD Patients: 56.3% Primary Caregivers: 73.2% Disease Status Alzheimer’s Disease  Number of Chronic Conditions  Not reported | ADL: 2.7 (1.4) | N/A | MMSE: 12 (7.9) |
| Schnaider-Beeri 2002b, Israel, prospective study | Size AD Patients: 121 Healthy: 50 Age AD Patients: 78.5 (9.5) Healthy: 72.1 (5.9)  %Female AD Patients: 62.8% Healthy: 88.0% Disease Status Alzheimer's Disease  Number of Chronic Conditions  Not reported | Participants with Alzheimer’s Disease  ADL: 3.2 (1.8) | N/A | Participants with Alzheimer’s Disease  MMSE: 9.0 (10.2) |

*Appendix Table 1 Continued*

| **Publication, Country, Study Design** | **Sample Characteristics** Size Mean Age (SD) %Female Disease Status  Number of Chronic Conditions | **Frailty Characteristics** IADL/ADL, FAI, Barthel Index, GFI  Mean (SD)^1^ | **Mobility Characteristics** SPPB, TUG, 6MW   Mean (SD)^1^ | **Global Cognition** MMSE, MoCA, GDS  Mean (SD)^1^ |
| --- | --- | --- | --- | --- |
| Schousboe 2019, USA, prospective cohort study | 2508 71.5 (10.5) 100% Not reported  Not reported | **By GDS score (%):** 0-1: - 0: 71.2 - 1: 15.1 - 2 or more: 13.8 2-5: - 0: 43.9 - 1: 21.3  - 2 or more: 34.9 6 or more: - 0: 23.1 - 1: 18.5 - 2 or more: 58.4 | N/A | MMSE score stratified by GDS score (%): - 0-1: 27.9 (2.0) - 2-5: 27.6 (2.3)  - 6 or more: 26.8 (2.8) |
| Schraeder 2008, USA, randomized controlled trial | Size 677 - Intervention: 400 - Control: 277 Age: - Intervention: 75.4 (7.1) - Control: 76.4 (7.9) %Female - Intervention: 53.4% - Control: 60.3% Disease Status  Not reported  Number of Chronic Conditions  42.1% with ≥1 chronic condition | 2 or more IADL limitations:  - intervention: 16.8% - control: 17.3% | N/A | N/A |
| Schwarzkopf 2011, Germany, cluster-randomized trial | 383 80.4 (6.8) 68.1% Not reported  Not reported | IADL hours per day at baseline: 2.4 (2.0)  Barthel: 73.3 (26.5) | N/A | MMSE: 18.7 (3.8) |

*Appendix Table 1 Continued*

| **Publication, Country, Study Design** | **Sample Characteristics** Size Mean Age (SD) %Female Disease Status  Number of Chronic Conditions | **Frailty Characteristics** IADL/ADL, FAI, Barthel Index, GFI  Mean (SD)^1^ | **Mobility Characteristics** SPPB, TUG, 6MW   Mean (SD)^1^ | **Global Cognition** MMSE, MoCA, GDS  Mean (SD)^1^ |
| --- | --- | --- | --- | --- |
| Scott 2004, USA, randomized controlled trial | Size 294 - Intervention: 145 - Control: 149  Age - Intervention: 74.2 (7.6) - Control: 74.1 (7.4)  %Female - Intervention: 61% - Control: 57% Disease Status Not reported  Number of Chronic Conditions  59.4% with ≥1 chronic condition | No basic ADL deficits: - Intervention: 33.8% - Control: 32.2%   No advanced ADL deficits: - Intervention: 97.9% - Control: 93.3%*.*  No household ADL deficits: - Intervention: 81.4% - Control: 79.9% | N/A | N/A |
| Taylor 2001, USA, observational | Size 4834 Age: - Severe Alzheimer’s disease and related dementias (ADRD): 81.4 - Moderate ADRD: 80.6 - No ADRD: 77 %Female N/A Disease Status Alzheimer's Disease  Number of Chronic Conditions  Not reported | Number of ADLs (0-6):  - Severe ADRD: 2.5 - Moderate ADRD: 1.7  Number of IADLs (0-6):  - Severe ADRD: 3.1 - Moderate ADRD: 1.7 | N/A | N/A |
| Van Lier 2016, Netherlands, within-subject study | 656 83.2 (7.2) 67% Not reported  % with multimorbidity: 57% | IADL: 25.4 (12.8) | N/A | N/A |

*Appendix Table 1 Continued*

| **Publication, Country, Study Design** | **Sample Characteristics** Size Mean Age (SD) %Female Disease Status  Number of Chronic Conditions | **Frailty Characteristics** IADL/ADL, FAI, Barthel Index, GFI  Mean (SD)^1^ | **Mobility Characteristics** SPPB, TUG, 6MW   Mean (SD)^1^ | **Global Cognition** MMSE, MoCA, GDS  Mean (SD)^1^ |
| --- | --- | --- | --- | --- |
| Wang 2008, China, observational study | By MMSE Severity:  Size 66 - Mild: 13 - Moderate: 37 - Severe: 16  Age 74.0 (8.6) - Mild: 70.6 (10.4) - Moderate: 73.5 (7.8) - Severe: 77.9 (8.07) %Female 65.2% - Mild: 61.5% - Moderate: 67.6% - Severe: 62.5% Disease Status Alzheimer’s Disease  Number of Chronic Conditions  54.5% had ≥1 chronic condition | N/A | N/A | MMSE: Mild (21-26): 19.7% Moderate (11-20): 56.1% Severe (0-10): 24.2% |

*Appendix Table 1 Continued*

| **Publication, Country, Study Design** | **Sample Characteristics** Size Mean Age (SD) %Female Disease Status  Number of Chronic Conditions | **Frailty Characteristics** IADL/ADL, FAI, Barthel Index, GFI  Mean (SD)^1^ | **Mobility Characteristics** SPPB, TUG, 6MW   Mean (SD)^1^ | **Global Cognition** MMSE, MoCA, GDS  Mean (SD)^1^ |
| --- | --- | --- | --- | --- |
| Wang 2010, China, cross-sectional | By cognitive impairment severity: Size 71 - Mild: 18 - Moderate: 43 - Severe: 10 Age: - Mild: 75.7 (4.8) - Moderate: 74.1 (8.4) - Severe: 72.4 (8.4) %Female - Mild: 18 - Moderate: 43 - Severe: 10 Disease Status Alzheimer’s Disease Number of Chronic Conditions  Not reported | By cognitive impairment severity:  ADL score: - Mild: 32.6 (6.5) - Moderate: 39.3 (8.5) - Severe: 49.1 (7.3) | N/A | MMSE:  Mild cognitive impairment (>= 21): n=18 Moderate cognitive impairment (11-20): n=43 Severe cognitive impairment (0-10): n=10 |
| Witham 2012, Scotland, parallel-group, single-blind, randomized controlled trial | Size 107 - Exercise: 53 - Control: 54 Age 80 - Exercise: 80.4 (5.8) - Control: 79.5 (4.9) %Female 33% - Exercise: 34% - Control: 31% Disease Status  Heart Failure  Number of Chronic Conditions  Not reported | N/A | 6MW: - Exercise: 266 (118) - Control: 267 (112) | MMSE, Median: - Exercise: 29 - Control: 29 |

*Appendix Table 1 Continued*

| **Publication, Country, Study Design** | **Sample Characteristics** Size Mean Age (SD) %Female Disease Status  Number of Chronic Conditions | **Frailty Characteristics** IADL/ADL, FAI, Barthel Index, GFI  Mean (SD)^1^ | **Mobility Characteristics** SPPB, TUG, 6MW   Mean (SD)^1^ | **Global Cognition** MMSE, MoCA, GDS  Mean (SD)^1^ |
| --- | --- | --- | --- | --- |
| Wolff 2019, USA, observational | Size 442 Age : household disability: - none: 73.8 (0.1) - no negative consequences: 76.8 (0.3) - negative consequences: 75.6 (0.5) mobility disability:  - none: 74.0 (0.1) - no negative consequences: 76.5 (0.3) - negative consequences: 77.2 (0.5) self-care disability - none: 74.0 (0.1) - no negative consequences: 76.9 (0.3) - negative consequences: 78.2 (0.7) %Female household disability: - none: 52% - no negative consequences: 61.1% -negative consequences: 60.1% mobility disability:  - none: 53.3% - no negative consequences: 56.9% - negative consequences: 67.2% self-care disability - none: 54.1% - no negative consequences: 56.4% - negative consequences: 66.3  Disease Status Not reported  Number of Chronic Conditions  2.98 (0.1) | all had 1 or more activity limitation | N/A | N/A |

*Appendix Table 1 Continued*

| **Publication, Country, Study Design** | **Sample Characteristics** Size Mean Age (SD) %Female Disease Status Number of Chronic Conditions | **Frailty Characteristics** IADL/ADL, FAI, Barthel Index, GFI  Mean (SD)^1^ | **Mobility Characteristics** SPPB, TUG, 6MW   Mean (SD)^1^ | **Global Cognition** MMSE, MoCA, GDS  Mean (SD)^1^ |
| --- | --- | --- | --- | --- |
| Wolstenholme 2002, UK, retrospective analysis of longitudinal data | 100 78 (7) 49% Not reported  Not reported | Barthel index mean not reported | N/A | N/A |
| Woods 2012, England & Wales, randomized controlled trial | Size 487 patient-carer dyads - Intervention: 268 - Control: 219 Age of Patients 77.53 (7.3) - Intervention: 77.72 (7.4) - Control: 77.30 (7.18) %Female 50% - Intervention: 47% - Control: 53% Disease Status All-cause Dementia  Number of Chronic Conditions  Not reported | Total score not reported  Bristol Activities of Daily Living Scale: Baseline: - Intervention: 16.61 (9.43) - Control: 15.06 (9.75) 3-Months: - Intervention: 18.13 (10.16) - Control: 16.53 (10.14) 10-Months: - Intervention: 19.67 (10.81) - Control: 19.03 (10.70) | N/A | N/A |

*Appendix Table 1 Continued*

| **Publication, Country, Study Design** | **Sample Characteristics** Size Mean Age (SD) %Female Disease Status  Number of Chronic Conditions | **Frailty Characteristics** IADL/ADL, FAI, Barthel Index, GFI  Mean (SD)^1^ | **Mobility Characteristics** SPPB, TUG, 6MW   Mean (SD)^1^ | **Global Cognition** MMSE, MoCA, GDS  Mean (SD)^1^ |  |
| --- | --- | --- | --- | --- | --- |
| Wübker 2014, 8 European countries (Estonia, Finland, France, Germany, the Netherlands, Spain, Sweden and the UK), prospective cohort study | Size - Mild: 340 - Moderate: 464 - Severe: 244 Age - Mild: 83 (7) - Moderate: 83 (7) - Severe: 81 (7) %Female - Mild: 78% - Moderate: 65% - Severe: 66% Disease Status Dementia  Number of Chronic Conditions  Not reported | ADL Independence: - Mild: 4.3 (1.6) - Moderate: 3.7 (1.7) - Severe: 2.3 (1.8) | N/A | SMMSE: - Mild: 21.2 (1.7) - Moderate: 14.4 (2.5) - Severe: 4.5 (3.1) | |
| Zhang 2003, USA, retrospective analysis of older fee-for-service HF patients, using the 1996 Medicare Current Beneficiary Survey and linked Medicare claims | 1269 Age 65–74: 22% 75–84: 40% 85 or more: 38% %Female 65% Disease Status Heart Failure Patients  Number of Chronic Conditions  % of participants with Charlson Comorbidity Index ≥1: 80% | IADL deficiencies: 0: 24% 1: 19% 2 or more: 57% | N/A | N/A | |

^1^ For studies that did not explicitly state year of currency used, the year of currency was assumed to be the same as the study base year.

^2^ Costs reported as Mean (SD) unless otherwise stated; non-adjusted costs in square brackets

^3^ Abbreviations: IADL: Instrumental Activities of Daily Living; ADL: Activities of Daily Living; FAI: Functional Aging Index; GFI: Groningen Frailty Index; SPPB: Short Performance Physical Battery; TUG: Timed Up and Go; 6MW: Six-minute walk test; MMSE: Mini-Mental State Examination; MoCA: Montreal Cognitive Assessment; GDS: Geriatric Depression Scale

**Appendix Table 2.** Cost Items Related to Impaired or Loss of Functional Independence

| **Publication** | **Cost Perspectives** Currency, Year^1^, Time Period Costs Were Measured, Discounting | **Total Cost of Outcomes Related to Impaired or Loss of Functional Independence**  Mean (SD)^2^ | **Societal Costs**  Mean (SD)^2^ | **Health Care Costs**  Mean (SD)^2^ | **Personal Costs**  Mean (SD)^2^ | **Sensitivity Analysis** |
| --- | --- | --- | --- | --- | --- | --- |
| Åkerborg 2016 | Assumed Societal, Health Care System, Personal  Euros €, 2012, 1-year, N/A | $37,382.32 ($30,271.22) [€43,259 (35,030)] | Home Health care: $2,868.12 ($5,539.21) [€3,319 (6,410)]  Accomodation: $25,539.13 ($29,883.21) [€29,554 (34,581)] | Inpatient: $2,908.73 ($6,830.25) [€3,366 (7,904)]  Outpatient: $2,608.01 ($5,958.32) [€3,018 (6,895)] | Home Help (Indirect): $3,459.20 ($5,615.25) [€4,003 (6,498)] | N/A |
| Akincigil 2020 | Assumed Health Care System  USD$, 2013, 1-year, N/A |  | Home Health care All: $711.10 [$632] IADL 2+: $1,679.86 [$1,493] | All Costs  All: $15,515.91 [$13,790] IADL 2+: $24,064.84 [$21,388]  Inpatient All: $4,771.79 [$4,241] IADL 2+: $8,646.83 [$7,685]  Outpatient All: $10,034.14 [$8,918] IADL 2+:  $13,738.16 [$12,210] |  | N/A |

*Appendix Table 2 Continued*

| **Publication** | **Cost Perspectives** Currency, Year^1^, Time Period Costs Were Measured, Discounting | **Total Cost of Outcomes Related to Impaired or Loss of Functional Independence**  Mean (SD)^2^ | **Societal Costs**  Mean (SD)^2^ | **Health Care Costs**  Mean (SD)^2^ | **Personal Costs**  Mean (SD)^2^ | **Sensitivity Analysis** |
| --- | --- | --- | --- | --- | --- | --- |
| Ankuda 2017 | Assumed Health Care System  USD$, 2012, 6-months, N/A |  |  | All Costs: $10,003.28 ($258.34) [$8,751 (226)] |  | N/A |
| Beech 1999 | Assumed health care system  Pounds Sterling £, 1997, 1-year, N/A |  |  | All Costs Intervention: $7,850.54 [£6,800] Control: $8,580.18 [£7,432]  Inpatient Intervention: $5,613.14 [£4,862] Control: $7,322.94 [£6,343]  Outpatient Intervention: $2,237.40 [£1,938] Control: $1,257.24 [£1,089] |  | One-way - controlling overhead rate and pre- and post-randomization average length of stay |

*Appendix Table 2 Continued*

| **Publication** | **Cost Perspectives** Currency, Year^1^, Time Period Costs Were Measured, Discounting | **Total Cost of Outcomes Related to Impaired or Loss of Functional Independence**  Mean (SD)^2^ | **Societal Costs**  Mean (SD)^2^ | **Health Care Costs**  Mean (SD)^2^ | **Personal Costs**  Mean (SD)^2^ | **Sensitivity Analysis** |
| --- | --- | --- | --- | --- | --- | --- |
| Bleijenberg 2017 | Societal  Euros €, 2012, 1-year, N/A |  | Day Care: $41.31/day [€47.80/day] | All Costs Intervention (Frailty Screening + GP Care): $4,258.54 ($9,874.66) [€4,928 (11,427)] Intervention (Frailty Screening + Nurse-Led Care): $4,153.11 ($6,491.50) [€4,806 (7,512)] Control: $4,857.39 ($10,619.55) [€5,621 (12,289)]  Clinical Intervention (Frailty Screening + GP Care): U-PRIM: $4,857.39 ($10,619.55) [€5,621 (12,289)] Intervention (Frailty Screening + Nurse-Led Care): $113.20 (0) [€131 (0)] Control: $0 (0) [€0 (0)]  Hospital: $419.37/day [€485.30/day]  Emergency Department Visits: $138.57/visit [€160.35/visit] | Informal Care Costs Intervention (Frailty Screening + GP Care): $1,464.74 ($5,928.94) [€1,695 (6,861)] Intervention (Frailty Screening + Nurse-Led Care): $1,631.52 ($6,870.00) [€1,888 (7,950)] Control: $1,711.02 ($6,992.71) [€1,980 (8,092)]  Home Help (Indirect): $32.15/hr [€37.20/hr] | 3 one-way sensitivity analyses varying the following parameters: health care perspective, unadjusted QALYs, and complete cases (adjusted QALY) |

*Appendix Table 2 Continued*

| **Publication** | **Cost Perspectives** Currency, Year^1^, Time Period Costs Were Measured, Discounting | **Total Cost of Outcomes Related to Impaired or Loss of Functional Independence**  Mean (SD)^2^ | **Societal Costs**  Mean (SD)^2^ | **Health Care Costs**  Mean (SD)^2^ | **Personal Costs**  Mean (SD)^2^ | **Sensitivity Analysis** | |
| --- | --- | --- | --- | --- | --- | --- | --- |
| Boult 2001 | Assumed societal  USD$, assumed 1997, 18-month, N/A |  | Home Health care: Control: $1,265.38 ($4,322.15) [$777 (2,654)] Intervention: $1,291.43 ($5,357.90) [$793 (3,290)] | All Costs Control: $19,193.99 ($31,297.32) [$11,786 (19,218)] Intervention: $18,490.46 ($30,540.04) [$11,354 (18,753)]  Hospital Control: $11,105.03 ($22,146.54) [$6,819 (13,599)] Intervention: $10,290.76 ($22,224.71) [$6,319 (13,647)]  Clinical Control: $3,680.50 ($4,880.74) [$2,260 (2,997)] Intervention: $3,706.56 ($4,151.15) [$2,276 (2,549)]  Outpatient Control: $1,148.12 ($1,965.65) [$705 (1,207)] Intervention: $1,304.46 ($2,099.19) [$801 (1,289)] |  | N/A |  |

*Appendix Table 2 Continued*

| **Publication** | **Cost Perspectives** Currency, Year^1^, Time Period Costs Were Measured, Discounting | **Total Cost of Outcomes Related to Impaired or Loss of Functional Independence**  Mean (SD)^2^ | **Societal Costs**  Mean (SD)^2^ | **Health Care Costs**  Mean (SD)^2^ | **Personal Costs**  Mean (SD)^2^ | **Sensitivity Analysis** |
| --- | --- | --- | --- | --- | --- | --- |
| Braithwaite 2003 | Societal  USD$, 2001, lifetime attributable costs, lifetime discount rate of 3% | Lifetime Attributable Cost: $120,302.18 [$81,300] Discounted 3%: $111,719.73 [$75,500] | All Home Health care: $9,174.34 [$6,200] | All Costs in First 6-Months: $39,804.78 [$26,900]  All Hospital Initial Hospitalization: $13,169.61 [$8,900] Subsequent Hospitalizations: $5,770.95 [$3,900]  Rehabilitation**:** $3,403.38 [$2,300] | All Informal Care: $36,401.40 [$24,600] | One-way |
| Brinda 2014 | Societal and Health Care System  Rupee ₹, 2012, 1-year, N/A |  |  |  | All Informal Care: $5,337.04 ($2,127.32) [₹75453.0 (₹30075.2)] | N/A |

*Appendix Table 2 Continued*

| **Publication** | **Cost Perspectives** Currency, Year^1^, Time Period Costs Were Measured, Discounting | **Total Cost of Outcomes Related to Impaired or Loss of Functional Independence**  Mean (SD)^2^ | **Societal Costs**  Mean (SD)^2^ | **Health Care Costs**  Mean (SD)^2^ | **Personal Costs**  Mean (SD)^2^ | **Sensitivity Analysis** |
| --- | --- | --- | --- | --- | --- | --- |
| Challis 2002 | Assumed societal  Pounds Sterling £, assumed 1999, 1-year, N/A |  | All Society Experimental: $26,786.31 [£23,402] Control: $21,808.37 [£19,053]  Assisted Services Experimental: $10,089.79 [£8,815] Control: $5,352.22 [£4,676]  Home Health care Experimental: $5,472.41 [£4,781] Control: $2,585.69 [£2,259]  Day Care: Experimental: $1,988.20 [£1,737] Control: $1,325.47 [£1,158] | NHS Experimental: $6,911.19 [£6,038] Control: $5,158.78 [£4,507]  Hospital Experimental: $5,234.33 [£4,573] Control: $3,496.80 [£3,055]  Clinical Experimental: $2,810.03 [£2,455] Control: $1,271.67 [£1,111]  Outpatient Experimental: $447.54 [£391] Control: $640.99 [£560] | Personal Expenditures Experimental: $3,552.89 [£3,104] Control: $3,556.32 [£3,107] | N/A |

*Appendix Table 2 Continued*

| **Publication** | **Cost Perspectives** Currency, Year^1^, Time Period Costs Were Measured, Discounting | | **Total Cost of Outcomes Related to Impaired or Loss of Functional Independence**  Mean (SD)^2^ | | **Societal Costs**  Mean (SD)^2^ | | **Health Care Costs**  Mean (SD)^2^ | | **Personal Costs**  Mean (SD)^2^ | | **Sensitivity Analysis** | |
| --- | --- | --- | --- | --- | --- | --- | --- | --- | --- | --- | --- | --- |
| Challis 2004 | | Assumed health care system  Pounds Sterling £, 2000/2001, 1-week, N/A | |  | | All Costs Experimental: $189.20 [£175] Control: $205.42 [£190] | | All Costs Experimental: $78.92 [£73] Control: $89.74 [£83] | | All Costs Experimental: $118.93 [£110] Control: $102.71 [£95] | | N/A |
| Chavan 2020 | | Assumed health care system  USD$, Assumed 2020, Cost per hospitalization (assumed annual) | |  | |  | | Hospital  **Having Physical Limitations**  Cancer Survivors: $22,971 ($2,478.40)  Non-cancer patients $20,068 ($854.20)  **Having ADL Limitations**  Cancer Survivors: $23,711 ($2,689.20)  Non-cancer patients $23,711 ($1,170.10)  **Having IADL Limitations**  Cancer Survivors: $24,916 ($3,877.10)  Non-cancer patients $22,699 ($1,533.40) | |  | | N/A |
| Chen 2000/2001 | | Assumed health care system  USD$, 1988, 6-weeks;1-year, N/A | |  | |  | | All Costs, By Discharge Location: **At 6-Weeks Post-Discharge** Stroke  - Home: $7,264.68 [$3,244] - Home Care: $8,859.15 [$3,956] - Rehab: $37,467.75 [$16,731] **At 1-Year Post-Discharge** Stroke - Home: $28,776.56 [$12,850] - Home Care: $29,759.66 [$13,289] - Rehab: $58,818.39 [$26,265] Hip Procedure - Home Care: $13,199.15 [$5,894] Hip Fracture - Home: $29,022.90 [$12,960] - Home Care: $20,080.89 $8,967] - Rehab: $61,586.31 [$27,501] | |  | | Taylor's approximation method to obtain the confidence intervals of the cost-effectiveness ratio |

*Appendix Table 2 Continued*

| **Publication** | **Cost Perspectives** Currency, Year^1^, Time Period Costs Were Measured, Discounting | **Total Cost of Outcomes Related to Impaired or Loss of Functional Independence**  Mean (SD)^2^ | **Societal Costs**  Mean (SD)^2^ | **Health Care Costs**  Mean (SD)^2^ | **Personal Costs**  Mean (SD)^2^ | **Sensitivity Analysis** |
| --- | --- | --- | --- | --- | --- | --- |
| Chiatti 2015 | Societal, Health Care System, Personal  Euros €, 2013, 6-months;1-year, N/A |  | All Costs Estimated Yearly Cost: $16,641.35 [€20,128] | All Costs Estimated Yearly Cost: $3,748.60 [€4,534]   Hospital Estimate Yearly Cost: $534.10 [€646]  Outpatient Estimated Yearly Cost: $33.07 [€40] | All Costs  Estimated Yearly Cost: $2,458.83 [€2,974]  Medications Estimated Yearly Cost: - Alzheimer's drugs: $737.48 [€892] - Other drugs: $421.66 [€510]  Informal Care Estimated Yearly Cost: $11,235.89 [€13,590] | N/A |

*Appendix Table 2 Continued*

| **Publication** | **Cost Perspectives** Currency, Year^1^, Time Period Costs Were Measured, Discounting | | **Total Cost of Outcomes Related to Impaired or Loss of Functional Independence**  Mean (SD)^2^ | | **Societal Costs**  Mean (SD)^2^ | | **Health Care Costs**  Mean (SD)^2^ | | **Personal Costs**  Mean (SD)^2^ | | **Sensitivity Analysis** |
| --- | --- | --- | --- | --- | --- | --- | --- | --- | --- | --- | --- |
| Chiu 2000 | | Assumed personal  USD$, 1996, 3-months, N/A | |  | |  | |  | All Costs 3-Months Post-Discharge - Hospital: $2,943.41 [$1,754] - Home Care: $7,401.33 [$4,410.50] - Family Care: $5,325.33 [$3,173.40]  Informal Care - Hospital: $0 [$0] - Home Care: $6,665.64 [$3,972.10] - Family Care: $4,605.59 [$2,744.50]  Direct Health-Care Related - Hospital: $154.22 [$91.90] - Home Care: $5.37 [$3.20] - Family Care: $9.73 [$5.80]   Out-of-Pocket Expenditures for Miscellaneous Items - Hospital: $635.00 [$378.40] - Home Care: $704.81 [$420] - Family Care: $710.01 [$423.10] | N/A | |

*Appendix Table 2 Continued*

| **Publication** | **Cost Perspectives** Currency, Year^1^, Time Period Costs Were Measured, Discounting | | **Total Cost of Outcomes Related to Impaired or Loss of Functional Independence**  Mean (SD)^2^ | | **Societal Costs**  Mean (SD)^2^ | | **Health Care Costs**  Mean (SD)^2^ | | **Personal Costs**  Mean (SD)^2^ | | | **Sensitivity Analysis** |  |
| --- | --- | --- | --- | --- | --- | --- | --- | --- | --- | --- | --- | --- | --- |
| Coleman 1999 | | Assumed health care system  USD$, Not Reported, 2-years, N/A | | Total Annual Cost Intervention - Baseline: $16,662.58 [$10,566] - 24-Month Follow-Up: $15,036.69 [$9,535] Control - Baseline: $16,695.69 [$10,587] - 24-Month Follow-Up: $15,952.93 [$10,116] | |  | |  | | Medications Intervention - Baseline: $1,340.45 [$850]  - 24-Month Follow-Up: $1,692.12 [$1,073] Control - Baseline: $1,501.30 [$952] - 24-Month Follow-Up: $1,688.97 [$1,071] | N/A | |  |
| Colon-Emeric 2020 | | Assumed health care system  USD$, 6 Months, 2020, N/A | |  | |  | | Hospital  **Costs reported by cost-trajectory group:**  Low variable: $9,972.39 ($1,931.49)  Low-spike-recover: $7,112.67 ($603.47)  Persistently moderate: $8,334.08 ($442.19)  Persistently high: $12,239 ($640.99) |  | | N/A | | |

*Appendix Table 2 Continued*

| **Publication** | **Cost Perspectives** Currency, Year^1^, Time Period Costs Were Measured, Discounting | **Total Cost of Outcomes Related to Impaired or Loss of Functional Independence**  Mean (SD)^2^ | **Societal Costs**  Mean (SD)^2^ | **Health Care Costs**  Mean (SD)^2^ | **Personal Costs**  Mean (SD)^2^ | **Sensitivity Analysis** |
| --- | --- | --- | --- | --- | --- | --- |
| Davis 2011 | Health Care System  CAD$, 2009, 21-months, N/A |  |  | All Costs - Control: $799.78 ($439.22) [$783 (430)] - Experimental (Once-Weekly Resistance Training): $1,150.13 ($2,047.97) [$1,126 (2,005)] - Experimental (Twice-Weekly Resistance Training): $1,625.10 ($3,247.14) [$1,591 (3,179)]  Hospital Cost (Per Day) - Control: $1,558.71 ($5,167.43) [$1,526 (5,059)] - Experimental (Once-Weekly Resistance Training): $697.64 ($2,249.20) [$683 (2,202)] - Experimental (Twice-Weekly Resistance Training): $600.60 ($3,326.81) [$588 (3,257)] |  | Probabilistic and selective one-way sensitivity analyses |

*Appendix Table 2 Continued*

| **Publication** | **Cost Perspectives** Currency, Year^1^, Time Period Costs Were Measured, Discounting | | **Total Cost of Outcomes Related to Impaired or Loss of Functional Independence**  Mean (SD)^2^ | | **Societal Costs**  Mean (SD)^2^ | | **Health Care Costs**  Mean (SD)^2^ | **Personal Costs**  Mean (SD)^2^ | | | **Sensitivity Analysis** |
| --- | --- | --- | --- | --- | --- | --- | --- | --- | --- | --- | --- |
| Deardorff 2019 | | Assumed Health Care System  USD, 2006, 1-year, N/A | |  | |  | Mean (95% CI)  All Costs Per Participant With Dementia - No VI or HI: $19,955.87 ($17,781.67-$22,395.32) [$15,273 (13,609-17,140)] - VI only: $27,139.62 ($22,349.58-$32,927.91) [$20,771 (17,105-25,201)] - HI only: $22,350.89 ($18,759.02-$26,541.19) [$17,106 (14,357-20,313)] - DSI: $25,344.34 ($21,552.55-$29,734.55) [$19,397 (16,495-22,757)]  Mean (95% CI)  All Costs Per Participant Without Dementia  - No VI or HI: $11,146.70 ($10,908.90-$11,391.04) [$8,531 (8,349-8,718)] - VI only: $12,930.22 ($12,484.67-$13,599.21) [$9,896 (9,555-10,408)] - HI only: $13,042.59 ($12,595.73-$13,599.21) [$9,982 (9,640-10,408)] - DSI: $4,364.08 ($4,195.53-$4,533.94) [$3,340 (3,211-3,470)]  **VI: visual impairment, HI: hearing impairment, DSI: dual-sensory impairment* | |  | N/A | |

*Appendix Table 2 Continued*

| **Publication** | **Cost Perspectives** Currency, Year^1^, Time Period Costs Were Measured, Discounting | | **Total Cost of Outcomes Related to Impaired or Loss of Functional Independence**  Mean (SD)^2^ | | **Societal Costs**  Mean (SD)^2^ | | | **Health Care Costs**  Mean (SD)^2^ | **Personal Costs**  Mean (SD)^2^ | | | | **Sensitivity Analysis** | |
| --- | --- | --- | --- | --- | --- | --- | --- | --- | --- | --- | --- | --- | --- | --- |
| Deb 2018 | | Assumed Health Care System  USD$, 2013, 1-year, N/A | | Mean (SE)  All Alzheimer's: $19,163.66 ($1,390.69) [$17,032 (1,236)] No Alzheimer's: $11,332.57 ($236.28) [$10,072 (210)] | | Mean (SE)  Home Health care Alzheimer's: $6,570.91 ($1,280.43) [$5,840 (1,138)] No Alzheimer's: $499.57 ($33.75) [$444 (30)] | Mean (SE)  Hospital Alzheimer's: $4,287.97 ($481.57) [$3,811 (428)] No Alzheimer's: $3,637.63 ($174.40) [$3,233 (155)]  Outpatient Alzheimer's: $2,890.53 ($329.67) [$2,569 (293)] No Alzheimer's: $3,495.86 ($81.01) [$3,107 (72)] | | | | Mean (SE)  Medications Alzheimer's: $4,287.97 ($249.78) [$3,811 (222)] No Alzheimer's: $2,737.51 ($55.13) [$2,433 (49)] | N/A | |  |
| Del Sindaco 2007 | | Assumed Health Care System  Euros €, 2001, 2-years, N/A | |  | |  | Hospital: $4,091.77 [€3,184.26] | | |  | | N/A | |  |
| Dodel 2015 | | Societal  Euros €, 2010, 1-month, N/A | |  | | All Costs, by MMSE Severity Mild: $2,035.70 [€2,226] Moderate: $2,278.05 [€2,491] Moderately Severe/Severe: $2,184.77 [€2,389] |  | | |  | | Sensitivity analyses included alternative GLM models based on assumptions that the costs were normally distributed, using a forward selection procedure | |  |

*Appendix Table 2 Continued*

| **Publication** | **Cost Perspectives** Currency, Year^1^, Time Period Costs Were Measured, Discounting | | **Total Cost of Outcomes Related to Impaired or Loss of Functional Independence**  Mean (SD)^2^ | | **Societal Costs**  Mean (SD)^2^ | | | **Health Care Costs**  Mean (SD)^2^ | **Personal Costs**  Mean (SD)^2^ | | | **Sensitivity Analysis** | |
| --- | --- | --- | --- | --- | --- | --- | --- | --- | --- | --- | --- | --- | --- |
| Eamer 2018 | | Assumed Health Care System and Personal  CAD$, 2016, 6-months, N/A | | Median (IQR)  All Costs: $449.48 ($126.87-$1,765.29) [$496 (140–1,948)]  According to Clinical Frailty Score: **1:** $991.39 ($272.77-$24,155.84) [$1,094 (301–26,656)] **2:** $271.86 ($87.00-$6,419.57) [$300 (96–7,084)] **3:** $324.42 ($104.21-$1,018.58) [$358 (115–1,124)]  **4:** $795.65 ($279.11-$3,291.34) [$878 (308–3,632)] **5:** $177.62 ($87.00-$490.26) [$196 (96–541)] **6:** $1,217.04 ($827.37-$3,796.10) [$1,343 (913–4,189)] | |  | Median (IQR)  Clinical:  $125.06 ($58.90-$300.86) [$138 (65–332)]  According to Clinical Frailty Score: **1:** $81.56 ($36.25-$1,942.91) [$90 (40–2,144)] **2:** $84.28 ($36.25-$177.62) [$93 (40–196)] **3:** $104.21 ($58.90-$298.14) [$115 (65–329)]  **4:** $241.05 ($116.90-$778.43) [$266 (129–859)] **5:** $81.56 ($36.25-$192.12) [$90 (40–212)] **6:** $750.34 ($299.95-$963.30) [$828 (331–1,063)] | | | Median (IQR)  Medications:  $0 ($0-$336.20) [$0 (0–371)]  According to Clinical Frailty Score: **1:** $0 ($0-$135.93) [$0 (0–150)] **2:** $0 ($0-$77.03) [$0 (0–85)] **3:** $0 ($0-$208.43) [$0 (0–230)]  **4:** $0 ($0-$438.60) [$0 (0–484)] **5:** $77.03 ($0-$414.14) [$85 (0–457)] **6:** $801.99 ($377.89-$2,971.45) [$885 (417–3,279)] | Assessed model fit using the Bayesian Information Criterion (BIC), lower BIC indicates a more plausible model given the data | |  |
| Eamer 2019 | | Health Care System  CAD$, 2016, 6-months, N/A | | $36,610.74 ($41,383.73) [$40,400 ($45,667)] | |  | All Costs 6-Month Post-discharge: $30,989.54 ($39,091.03) [$34,197 ($43,137)] | | | All Costs 6-Month Post-discharge: $2,743.09 ($7,319.43) [$3,027 ($8,077)] | N/A | |  |

*Appendix Table 2 Continued*

| **Publication** | **Cost Perspectives** Currency, Year^1^, Time Period Costs Were Measured, Discounting | | **Total Cost of Outcomes Related to Impaired or Loss of Functional Independence**  Mean (SD)^2^ | | **Societal Costs**  Mean (SD)^2^ | | | **Health Care Costs**  Mean (SD)^2^ | **Personal Costs**  Mean (SD)^2^ | | | **Sensitivity Analysis** | |
| --- | --- | --- | --- | --- | --- | --- | --- | --- | --- | --- | --- | --- | --- |
| Ensrud 2018 | | Health Care System  USD$, 2014, 1-year, N/A | |  | |  | All Costs: $7,399.21 ($13,808.18) [$6,680 (12,466)]  Outpatient: $2,588.62 ($3,774.93) [$2,337 (3,408)] | | |  | N/A | |  |
| Fairhall 2015 | | Health Care System  AUD$, 2011, 1-year, N/A | |  | | Community Services: $3,135.51 ($3,904.01) [$4,027 ($5,014)]  Home Health care Intervention: $1,125.89 ($2,022.86) [$1,446 ($2,598)] Control: $866.61 ($1,489.51) [$1,113 ($1,913)]  Meal Delivery Intervention: $263.17 ($656.38) [$338 ($843)] Control: $64.63 ($284.98) [$83 ($366)] | Hospital: $15,118.52 ($22,802.74) [$19,417 ($29,286)] Intervention: $15,532.74 ($22,239.02) [$19,949 ($28,562)] Control: $14,710.52 ($23,431.87) [$18,893 ($30,094)]  Primary Care: $837.02 ($587.08) [$1,075 ($754)] Intervention: $843.25 ($654.82) [$1,083 ($841)] Control: $830.79 ($513.11) [$1,067 ($659)]  Health Professional: $787.97 ($1,535.44) [$1,012 ($1,972)] Intervention: $886.07 ($1,861.69) [$1,138 ($2,391)] Control: $689.08 ($1,116.54) [$885 ($1,434)] | | |  | N/A | |  |

*Appendix Table 2 Continued*

| **Publication** | **Cost Perspectives** Currency, Year^1^, Time Period Costs Were Measured, Discounting | **Total Cost of Outcomes Related to Impaired or Loss of Functional Independence**  Mean (SD)^2^ | **Societal Costs**  Mean (SD)^2^ | **Health Care Costs**  Mean (SD)^2^ | **Personal Costs**  Mean (SD)^2^ | **Sensitivity Analysis** |
| --- | --- | --- | --- | --- | --- | --- |
| Farre 2016 | Societal  Euros €, 2010, 1-month, N/A | **MMSE (Disease Severity)** Mild: $1,358.78 ($1,230.66) [€1,485.8 (1,345.7)] Moderate: $1,607.62 ($1,214.56) [€1,757.9 (1,328.1]) Moderately Severe/Severe: $2,117.55 ($1,244.10) [€2,315.5 (1,360.4)]  **Katz Index (ADL) Functional Independence:** $1,412.19 ($1,395.82) [€1,544.2 (1,526.3)] Partial Disability: $1,914.53 ($1,144.42)[€2,093.5 (1,251.4)] Total Disability: $2,025.92 ($1,446.12) [€2,215.3 (1,581.3)] |  | Inpatient **MMSE (Disease Severity)** Mild: $168.82 ($740.02) [€184.6 (809.2)] Moderate: $61.59 ($245.22) [€67.35 (268.14)] Moderately Severe/Severe: $44.08 ($253.69) [€48.2 (277.4)] **Katz (ADL)** Functional Independence: $108.64 ($539.84) [€118.8 (590.3)] Partial Disability: $47.19 ($258.17) [€51.6 (282.3)] Total Disability: $202.02 ($808.98) [€220.9 (884.6)]  Outpatient **MMSE (Disease Severity)** Mild: $58.53 ($66.94) [€64.0 (73.2)] Moderate: $93.39 ($313.22) [€102.12 (342.5)] Moderately Severe/Severe: $69.23 ($117.15) [€75.7 (128.1)] **Katz (ADL)** Functional Independence: $78.74 ($326.57) [€86.1 (357.1)] Partial Disability: $77.18 ($128.31) [€84.4 (140.3)] Total Disability: $75.63 ($112.03) [€82.7 (122.5)] | Medications **MMSE (Disease Severity)** Mild: $45.54 ($43.71) [€49.8 (47.8)] Moderate: $48.91 ($42.94) [€53.48 (46.95)] Moderately Severe/Severe: $39.60 (40.6113579) [€43.3 (53.1)] **Katz (ADL)** Functional Independence: $41.70 ($43.53) [€45.6 (47.6)] Partial Disability: $48.19 ($44.72) [€52.7 (48.9)] Total Disability: $32.56 ($45.45) [€35.6 (49.7)]  Informal Care **MMSE (Disease Severity)** Mild: $732.80 ($601.47) [€801.30 (657.7)] Moderate: $980.18 ($770.06) [€1,071.81 (842.05)] Moderately Severe/Severe: $1,379.82 ($928.87) [€1,508.8 (1,015.7)] **Katz (ADL)** Functional Independence: $811.63 ($793.80) [€887.5 (868.0)] Partial Disability: $1,179.26 ($798.64) [€1,289.5 (873.3)] Total Disability: $1,339.76 ($814.28) [€1,465.0 (890.4)] | One-way - controlling distinct valuation rates of informal caregivers’ time |

*Appendix Table 2 Continued*

| **Publication** | **Cost Perspectives** Currency, Year^1^, Time Period Costs Were Measured, Discounting | **Total Cost of Outcomes Related to Impaired or Loss of Functional Independence**  Mean (SD)^2^ | **Societal Costs**  Mean (SD)^2^ | **Health Care Costs**  Mean (SD)^2^ | **Personal Costs**  Mean (SD)^2^ | **Sensitivity Analysis** |
| --- | --- | --- | --- | --- | --- | --- |
| Forster 2009 | Assumed Health Care System  Pounds Sterling £, 2003, 8-months, N/A | Intervention: $2,943.43 ($4,098.75) [£2,963 (4,126)] Control: $3,138.13 ($3,955.70) [£3,159 (3,982)] |  | Hospital Intervention: $1,235.78 ($3,927.88) [£1,244 (3,954)] Control: $1,471.22 ($3,330.85) [£1,481 (3,353)]  Outpatient Intervention: $840.41 ($621.87) [£846 (626)] Control: $835.45 ($696.37) [£841 (701)] | Medications Intervention: $12.91 ($3.97) [£13 (4)] Control: $12.91 ($8.94) [£13 (9)] | N/A |
| Graff 2008 | Societal  Euros €, assumed 2005, 3-month, N/A | Intervention: $14,511.04 ($7,655.75) [€12,563 (6,628)] Usual Care: $16,530.09 ($9,047.60) [€14,311 (7,833)] | Assisted Services Intervention: $30.03 ($78.54) [€26 (68)] Control: $39.27 ($103.96) [€34 (90)]  Home Health care Intervention: $1,746.45 ($2,851.85) [€1,512 (2,469)] Control: $2,228.11 ($3,697.35) [€1,929 (3,201)]  Day Care Intervention: $471.27 ($1,360.66) [€408 (1,178)] Control: $698.81 ($1,491.18) [€605 (1,291)]  Meals-on-Wheels Intervention: $154.78 ($315.33) [€134 (273)] Control: $164.02 ($321.11) [€142 (278)] | Physiotherapy Intervention: $175.57 ($334.97) [€152 (290)] Control: $217.15 ($419.29) [€188 (363)]  Hospital Intervention: $853.59 ($3,713.52) [€739 (3,215)] Control: $1,133.12 ($4,751.92) [€981 (4,114)] | Home Help (Indirect Costs) Intervention: $477.04 ($1,143.51) [€413 (990)] Control: $582.15 ($1,219.75) [€504 (1,056)] | One-way |

*Appendix Table 2 Continued*

| **Publication** | **Cost Perspectives** Currency, Year^1^, Time Period Costs Were Measured, Discounting | **Total Cost of Outcomes Related to Impaired or Loss of Functional Independence**  Mean (SD)^2^ | **Societal Costs**  Mean (SD)^2^ | **Health Care Costs**  Mean (SD)^2^ | **Personal Costs**  Mean (SD)^2^ | **Sensitivity Analysis** |
| --- | --- | --- | --- | --- | --- | --- |
| Gustavsson 2010 | Societal  Euros €, 2006, 1-year, N/A | Mean (95% CI) $8,462.37 ($7,784.95 – $9,139.79) [€7,820 (€7,194 – €8,446)] | Home Health care: $2,538.71 [€2346] | Direct Medical Costs: $1,353.98 [€1,251.2] | Informal Care: $241.10 [€222.8] | N/A |
| Han 2019 | Health Care System  Pounds Sterling £, 2013/14, 1-year, N/A |  |  | All Costs Non-frail: $1,273.80 [£1,628.35] Mild frailty: $1,712.69 [£2,189.40] Moderate frailty: $2,219.24 [£2,836.95] Severe frailty: $2,922.96 [£3,736.55] |  | N/A |
| Hardy 2010 | Assumed Heath Care System  USD$, 2003, 1-year, N/A |  |  | Mean (95% CI)  All Costs  **By IADL Limitations** No difficulty: $13,561.09 ($12,548.65-$14,559.28) [$9,510 (8,800-10,210)] Difficulty: $17,511.06 ($15,928.22-$19,093.90) [$12,280 (11,170-13,390)] Unable: $19,136.68 ($16,726.77-$21,560.85) [$13,420 (11,730-15,120)] | Mean (95% CI)  All Costs  **By IADL Limitations** No difficulty: $2,495.47 ($2,281.57-$2,723.63) [$1,750 (1,600-1,910)] Difficulty: $2,894.74 ($2,552.51-$3,222.72) [$2,030 (1,790-2,260)] Unable: $2,638.07 ($2,295.83-$2,994.56) [$1,850 (1,610-2,100)] | N/A |
| Harrow 2004 | Assumed health care system and personal  USD$, 1997, 1-month;1-year, N/A |  | Home Health care Monthly Cost: $1,094.38 [$672] Annual Cost: $13,132.56 [$8,064] |  | Informal Care Monthly Cost: $3,180.54 [$1,953] Annual Cost: $38,166.51 [$23,436] | N/A |

*Appendix Table 2 Continued*

| **Publication** | **Cost Perspectives** Currency, Year^1^, Time Period Costs Were Measured, Discounting | **Total Cost of Outcomes Related to Impaired or Loss of Functional Independence**  Mean (SD)^2^ | **Societal Costs**  Mean (SD)^2^ | **Health Care Costs**  Mean (SD)^2^ | **Personal Costs**  Mean (SD)^2^ | **Sensitivity Analysis** |
| --- | --- | --- | --- | --- | --- | --- |
| Hay 2002 | Societal  USD$, 1995, 1-year, N/A | Intervention At treatment: $8,172.97 ($20,090.24) [$4,741 (11,654)] At follow-up: $7,145.53 ($18,619.76) [$4,145 (10,801)]  Combined Control At treatment: $8,141.94 ($19,516.18) [$4,723 (11,321)] At follow-up: $8,995.27 ($16,528.68) [$5,218 (9,588)] |  | All Costs Intervention - At treatment: $4,337.31 ($11,091.52) [$2,516 (6,434)] - At follow-up: $1,667.00 ($3,116.80) [$967 (1,808)] Combined Control - At treatment: $3,742.57 ($10,712.27) [$2,171 (6,214)] - At follow-up: $4,470.05 ($10,201.99) [$2,593 (5,918)]  Program Costs - Intervention: $944.69 ($548) - Combined Control: $117.22 ($68) | Caregiver Costs (include paid and unpaid home help): Intervention - At treatment: $3,837.38 ($13,401.54) [$2,226 (7,774)] - At follow-up: $5,476.81 ($17,971.58) [$3,177 (10,425)] Combined Control - At treatment: $4,397.65 ($11,944.85) [$2,551 (6,929)] - At follow-up: $4,525.22 ($12,267.22) [$2,625 (7,116)] | N/A |
| Hektoen 2016 | Assumed Societal and Health Care System  Euros €, 2013, 1-year, N/A | $56,531.64 ($40,580.65) [€68,376 (49,083)] | Meals-on-Wheels: $2,028.91 ($925.16) [€2,454 (1,119)]  Day Care: $2,152.10 ($6,682.83) [€2,603 (8,083)]  Home Health care: $2,114.07 ($4,610.10) [€2,557 (5,576)] | Hospital: $17,721.95 ($15,425.99) [€21,435 (18,658)]  Outpatient: $2,510.92 ($5,368.26) [€3,037 (6,493)]  GP Visits: $770.56 ($670.52) [€932 (811)]  Rehabilitation: $8,129.69 ($9,321.07) [€9,833 (11,274)] | Medications: $320.79 ($320.79) [€388 (388)] | N/A |

*Appendix Table 2 Continued*

| **Publication** | **Cost Perspectives** Currency, Year^1^, Time Period Costs Were Measured, Discounting | **Total Cost of Outcomes Related to Impaired or Loss of Functional Independence**  Mean (SD)^2^ | **Societal Costs**  Mean (SD)^2^ | **Health Care Costs**  Mean (SD)^2^ | **Personal Costs**  Mean (SD)^2^ | **Sensitivity Analysis** |
| --- | --- | --- | --- | --- | --- | --- |
| Hendriks 2008 | Societal  Euros €, 2004, 1-year, N/A | Intervention: $5,751.95 ($5,293.64) [€4,857 (4,470)] Control: $5,910.64 ($8,094.41) [€4,991 (6,835)] |  | All Costs Intervention: $4,546.37 ($4,390.05) [€3,839 (3,707)] Control: $4,892.18 ($7,147.01) [€4,131 (6,035)]  Hospital Intervention: $1,459.01 ($1,933.90) [€1,232 (1,633)] Control: $2,087.85 ($3,505.41) [€1,763 (2,960)]  GP Visits Intervention: $190.67 ($202.51) [€161 (171)] Control: $225.01 ($248.69) [€190 (210)]  Paramedics and Alternative Medicine Intervention: $445.28 ($743.71) [€376 (628)] Control: $431.07 ($606.34) [€364 (512)]  Formal Care Intervention: $1,325.19 ($2,591.16) [€1,119 (2,188)] Control: $1,657.96 ($5,374.17) [€1,400 (4,538)] | All Costs Intervention: $1,205.58 ($1,923.24) [€1,018 (1,624)] Control: $1,018.46 ($2,311.67) [€860 (1,952)]  Medications Intervention: $176.45 ($150.40) [€149 (127)] Control: $177.64 ($144.48) [€150 (122)]  Informal Care Intervention: $528.18 ($1,549.01) [€446 (1,308)] Control: $387.25 ($1,466.11) [€327 (1,238)]  Home Help (Indirect Costs) Intervention: $324.49 ($840.82) [€274 (710)] Control: $344.62 ($708.19) [€291 (598)]  Home Modifications Intervention: $352.91 ($987.67) [€298 (834)] Control: $286.59 ($1,084.78) [€242 (916)]  Medical Aids and Assistive Devices Intervention: $420.41 ($932.01) [€355 (787)] Control: $242.77 ($498.57) [€205 (421)] | One-way |

*Appendix Table 2 Continued*

| **Publication** | **Cost Perspectives** Currency, Year^1^, Time Period Costs Were Measured, Discounting | **Total Cost of Outcomes Related to Impaired or Loss of Functional Independence**  Mean (SD)^2^ | **Societal Costs**  Mean (SD)^2^ | **Health Care Costs**  Mean (SD)^2^ | **Personal Costs**  Mean (SD)^2^ | **Sensitivity Analysis** |
| --- | --- | --- | --- | --- | --- | --- |
| Hughes 2000 | Assumed health care system  USD$, 1996, 1-year, N/A |  | Home Health care (Non-VA Use) Intervention: $2,000.31 ($7,710.94) [$1,192 (4,595)] Control: $3,873.09 ($9,253.13) [$2,308 (5,514)] | All Costs Intervention: $52,694.50 ($54,746.83) [$31,401 (32,624)] Control: $47,000.65 ($51,372.14) [$28,008 (30,613)]  All Costs, Veteran Affairs (VA) Intervention: $42,983.24 ($45,877.99) [$25,614 (27,339)] Control: $36,393.29 ($43,142.67) [$21,687 (25,709)]  All Costs, Non-VA Intervention: $9,711.25 ($28,816.60) [$5,787 (17,172)] Control: $10,607.37 ($22,167.90) [$6,321 (13,210)]  Inpatient (VA Use) - Intervention: $21,983.31 ($35,977.11) [$13,100 (21,439)] - Control: $21,686.28 ($34,257.05) [$12,923 (20,414)]  Inpatient (Non-VA Use) - Intervention: $3,441.81 ($16,534.47) [$2,051 (9,853)] - Control: $2,857.83 ($10,845.66) [$1,703 (6,463)]  Outpatient (VA Use) - Intervention: $6,355.02 ($9,425.97) [$3,787 (5,617)] - Control Group: $6,928.94 ($9,316.90) [$4,129 (5,552)]  Outpatient (Non-VA Use) - Intervention: $401.07 ($2,790.71) [$239 (1,663)] - Control: $429.60 ($2,307.41) [$256 (1,375)] |  | 2 one-way |

*Appendix Table 2 Continued*

| **Publication** | **Cost Perspectives** Currency, Year^1^, Time Period Costs Were Measured, Discounting | **Total Cost of Outcomes Related to Impaired or Loss of Functional Independence**  Mean (SD)^2^ | **Societal Costs**  Mean (SD)^2^ | **Health Care Costs**  Mean (SD)^2^ | **Personal Costs**  Mean (SD)^2^ | **Sensitivity Analysis** |
| --- | --- | --- | --- | --- | --- | --- |
| Hui 1995 | Assumed Health Care System  HKD$, 1992, 6-months, N/A | $16,732.41 ($7,449.73) [$58,168 (25,898)] |  | Hospital: $605.52/day [$2,105/day]  Rehabilitation: $261.77/day [$910/day]  Outpatient: $90.04/visit [$313/visit] |  | N/A |
| Joling 2015 | Societal  Euros €, 2009, 1-year, Costs in the second follow-up year were discounted by 4% | Mean (SE) Year 1: $66,693.04 ($3,915.93) [€71,514 (4,199)] Year 2: $87,968.15 ($6,041.30) [€94,327 (6,478)] | Mean (SE)  Home Health care Year 1: $1,468.82 ($364.64) [€1,575 (391)] Year 2: $4,139.75 ($1,687.98) [€4,439 (1,810)] | Mean (SE)  Outpatient Year 1: $978.28 ($77.40) [€1,049 (83)] Year 2: $1,650.68 ($206.10) [€1,770 (221)]  Inpatient Year 1: $16,494.67 ($1,812.02) [€17,687 (1,943)] Year 2: $31,406.74 ($3,135.36) [€33,677 (3,362)] | Mean (SE)  Medications Year 1: $982.01 ($72.74) [€1,053 (78)] Year 2: $1,201.17 ($166.00) [€1,288 (178)]  Informal Care Year 1: $46,769.24 ($2,929.26) [€50,150 (3,141)] Year 2: $49,569.80 ($3,462.70) [€53,153 (3,713)] | Two sensitivity analyses were carried out replicating the prediction models using different assumptions for people who died or were institutionalized during the study |

*Appendix Table 2 Continued*

| **Publication** | **Cost Perspectives** Currency, Year^1^, Time Period Costs Were Measured, Discounting | **Total Cost of Outcomes Related to Impaired or Loss of Functional Independence**  Mean (SD)^2^ | **Societal Costs**  Mean (SD)^2^ | **Health Care Costs**  Mean (SD)^2^ | **Personal Costs**  Mean (SD)^2^ | **Sensitivity Analysis** |
| --- | --- | --- | --- | --- | --- | --- |
| Kehusmaa 2013 | Assumed Societal, Health Care System, and Personal   Euros €, 2010, 1-year, discounted to year 2010 | Received formal care only: $23,137.14 [€25,300]  Received informal care from co-resident family member: $4,481.11 [€4,900]  Received informal care only for elderly living alone: $5,487.07 [€6,000]  Received a combination of formal and informal care: $4,196.56 [€22,300] |  |  |  | N/A |
| Komisar 1997 | Health Care System  USD$, 1992, 1-year, N/A | $22,101.45 [$11,780] | Home Health care: $4,521.60 [$2,410] | Hospital: $11,613.58 [$6,190]  Physician: $3,902.46 [$2,080]  Outpatient: $975.62 [$520] |  | N/A |

*Appendix Table 2 Continued*

| **Publication** | **Cost Perspectives** Currency, Year^1^, Time Period Costs Were Measured, Discounting | **Total Cost of Outcomes Related to Impaired or Loss of Functional Independence**  Mean (SD)^2^ | **Societal Costs**  Mean (SD)^2^ | **Health Care Costs**  Mean (SD)^2^ | **Personal Costs**  Mean (SD)^2^ | **Sensitivity Analysis** |
| --- | --- | --- | --- | --- | --- | --- |
| Kramer 1997 | Health Care System  USD$, assumed 1991, 6-months, N/A |  |  | All Costs Hip Fracture Patients: $33,113.34 [$17,202] Stroke Patients: $44,530.34 [$23,133]  Rehabilitation Hip Fracture Patients: $18,753.06 [$9,742] Stroke Patients: $26,044.85 [$13,530]  Hospital Hip Fracture Patients: $5,686.36 [$2,954] Stroke Patients: $6,356.25 [$3,302]  Outpatient Hip Fracture Patients: $508.19 [$264] Stroke Patients: $1,751.72 [$910] |  | N/A |

*Appendix Table 2 Continued*

| **Publication** | **Cost Perspectives** Currency, Year^1^, Time Period Costs Were Measured, Discounting | **Total Cost of Outcomes Related to Impaired or Loss of Functional Independence**  Mean (SD)^2^ | **Societal Costs**  Mean (SD)^2^ | **Health Care Costs**  Mean (SD)^2^ | **Personal Costs**  Mean (SD)^2^ | **Sensitivity Analysis** |
| --- | --- | --- | --- | --- | --- | --- |
| Kronborg 2006 | Societal  75 y/o: Danish Crown, 2002, 3-years 80 y/o: Kroner, 2002, 3-years  Present values using a 3% and 6% discount rate | Mean (SE)  Intervention  75 y/o: $2,204.20 ($103.44) [12,899 (605.36)] 80 y/o: $2,871.04 ($215.20) [17,773 (1,332.17)]   Control  75 y/o: $2,354.41 ($100.47) [13,778 (587.94)] 80 y/o: $2,755.70 ($190.77) [17,059 (1,180.97)] | Intervention [Mean (SE)] Home Health care 75 y/o: $415.75 ($42.01) [2,433 (245.84)] 80 y/o: $834.35 ($95.80) [5,165 (593.03)] Day Care 75 y/o: $59.30 ($9.86) [347 (57.68)] 80 y/o: $92.40 ($21.87) [572 (135.39)] Meals-on-Wheels 75 y/o: $46.99 ($5.19) [275 (30.39)] 80 y/o: $110.98 ($14.11) [687 (87.34)]  Control [Mean (SE)] Home Health care 75 y/o: $384.31 ($40.82) [2,249 (238.87)] 80 y/o: $688.81 ($84.91) [4,264 (525.60)] Day Care 75 y/o: $49.38 ($9.57) [289 (56.03)] 80 y/o: $59.12 ($19.39) [366 (120.02)] Meals-on-Wheels 75 y/o: $45.28 ($5.04) [265 (29.49)] 80 y/o: $87.88 ($12.52) [544 (77.52)] | Intervention [Mean (SE)] Hospital 75 y/o: $994.36 ($51.36) [5,819 (300.54)] 80 y/o: $1,007.20 ($95.78) [6,235 (592.90)] Rehabilitation 75 y/o: $23.24 ($3.43) [136 (20.09)] 80 y/o: $26.49 ($5.63) [164 (34.83)] Medical Specialist 75 y/o: $30.08 ($1.63) [176 (9.56)] 80 y/o: $27.62 ($3.51) [171 (21.75)] GP Visits 75 y/o: $90.74 ($1.91) [531 (11.19)] 80 y/o: $88.04 ($3.32) [545 (20.54)] Physiotherapy 75 y/o: $14.18 ($2.01) [83 (11.76)] 80 y/o: $13.57 ($3.85) [84 (23.85)]  Control [Mean (SE)] Hospital 75 y/o: $1,032.30 ($49.89) [6,041 (291.96)] 80 y/o: $1,126.09 ($84.91) [6,971 (525.60)] Rehabilitation 75 y/o: $13.67 ($3.34) [80 (19.52)] 80 y/o: $12.44 ($4.99) [77 (30.87)] Medical Specialist 75 y/o: $32.98 ($1.59) [193 (9.29)] 80 y/o: $38.12 ($3.11) [236 (19.28)] GP Visits 75 y/o: $96.38 ($1.86) [564 (10.87)] 80 y/o: $93.37 ($2.94) [578 (18.21)] Physiotherapy 75 y/o: $16.23 ($1.95) [95 (11.42)] 80 y/o: $13.41 ($3.42) [83 (21.15)] | Intervention [Mean (SE)]  Medications 75 y/o: $15.21 ($1.85) [89 (10.83)] 80 y/o: $17.77 ($2.99) [110 (18.49)]  Control  [Mean (SE)**]** Medications 75 y/o: $13.67 ($1.80) [80 (10.52)] 80 y/o: $21.65 ($3.37) [134 (20.85)] | Performed sensitivity analysis by varying the unit costs and assumptions used at prespecified intervals |

*Appendix Table 2 Continued*

| **Publication** | **Cost Perspectives** Currency, Year^1^, Time Period Costs Were Measured, Discounting | **Total Cost of Outcomes Related to Impaired or Loss of Functional Independence**  Mean (SD)^2^ | **Societal Costs**  Mean (SD)^2^ | **Health Care Costs**  Mean (SD)^2^ | **Personal Costs**  Mean (SD)^2^ | **Sensitivity Analysis** |
| --- | --- | --- | --- | --- | --- | --- |
| Ku 2019 | Assumed Societal, Health Care System, and Personal  NTD$, 2016, 1-year, N/A | $48,707.59 ($40,922.38) [$264,190 (221,963)] | All Costs: $17,124.45 ($24,816.19) [$92,883 (134,603)] | All Costs: $12,029.87 ($15,432.34) [$65,250 (83,705)] | All Costs (Informal Care): $19,553.28 ($28,224.19) [$106,057 (153,088)] | N/A |
| Lafortune 2020 | Assumed Health Care System and Personal  CAD$, 2000, 1-day, N/A | $87.52 ($28.87) [$69.99 (23.09)] | Community-Based Care: $25.60 ($8.00) [$20.47 (6.40)] | Acute Hospitalization: $42.90 ($16.24) [$34.31 (12.99)]  Institution-Based Care: $63.55 ($28.63) [$50.82 (22.90)] |  | Authors ran several sensitivity analyses |
| Langa 2004 | Assumed personal  USD$, 2000, 1-year, N/A |  |  |  | Mean (95% CI)  Informal Care:  No depressive symptoms: $1,903.35 ($1,841.95-$2,041.49) [$1,240 (1,200-1,330)] 1-3 depressive symptoms: $2,824.32 ($2,686.18-$2,885.72) [$1,840 (1,750-1,880)] 4-8 depressive symptoms: $3,944.84 ($3,745.30-$4,067.64) [$2,570 (2,440-2,650)] | Authors performed a sensitivity analysis for annual national caregiving costs |

*Appendix Table 2 Continued*

| **Publication** | **Cost Perspectives** Currency, Year^1^, Time Period Costs Were Measured, Discounting | **Total Cost of Outcomes Related to Impaired or Loss of Functional Independence**  Mean (SD)^2^ | **Societal Costs**  Mean (SD)^2^ | **Health Care Costs**  Mean (SD)^2^ | **Personal Costs**  Mean (SD)^2^ | **Sensitivity Analysis** |
| --- | --- | --- | --- | --- | --- | --- |
| Leeuwen 2015 | Societal   USD$, 2011, 6-Months, discounted by 4% in the second year of follow-up |  | Mean (SE)  Home Health care Intervention: $3,027.24 ($154.13) [$2,573 (131)] Control: $2,427.20 ($188.25) [$2,063 (160)]  Assisted Services Intervention: $495.32 ($42.36) [$421 (36)] Control: $417.67 ($50.59) [$355 (43)] | Mean (SE)  Hospital Intervention: $791.81 ($70.59) [$673 (60)] Control: $983.59 ($120.01) [$836 (102)]  Outpatient Intervention: $611.80 ($41.18) [$520 (35)] Control: $805.93 ($77.65) [$685 (66)]  Physiotherapy & OT Intervention: $489.44 ($28.24) [$416 (24)] Control: $521.21 ($34.12) [$443 (29)] | Mean (SE) Medications Intervention: $818.87 ($61.18) [$696 (52)] Control: $975.35 ($103.54) [$829 (88)]  Informal Care Intervention: $2,884.87 ($198.84) [$2,452 (169)] Control: $2,658.98 ($248.25) [$2,260 (211)] | A second sensitivity analysis was performed using a health care system perspective, including only direct health care costs and excluding costs related to informal care time. |
| Lewin 2013 | Health Care System, Personal  AUD$, 2007, 2-years, N/A | **Intention to Treat** - Control: $20,405.96 [$22,757] - Intervention: $17,833.36 [$19,888]  **Actual Treatment** - Control: $21,007.64 [$23,428] - Intervention: $17,117.80 [$19,090] |  | Hospital **Intention to Treat** - Control: $12,262.23 [$13,675] - Intervention: $11,987.84 [$13,369] **Actual Treatment** - Control: $12,697.12 [$14,160] - Intervention: $11,532.32 [$12,861]  Emergency **Intention to Treat** - Control: $634.86 [$708] - Intervention: $615.13 [$686] **Actual Treatment** - Control: $651.00 [$726] - Intervention: $590.92 [$659] |  | N/A |

*Appendix Table 2 Continued*

| **Publication** | **Cost Perspectives** Currency, Year^1^, Time Period Costs Were Measured, Discounting | **Total Cost of Outcomes Related to Impaired or Loss of Functional Independence**  Mean (SD)^2^ | **Societal Costs**  Mean (SD)^2^ | **Health Care Costs**  Mean (SD)^2^ | **Personal Costs**  Mean (SD)^2^ | **Sensitivity Analysis** |
| --- | --- | --- | --- | --- | --- | --- |
| Liotta 2019 | Health Care System  Euros €, 2014, 1-year, N/A |  |  | All Costs: $5,536.57 [€6,778.63]  Hospital Admissions: $4,506.66 [€5,517.67]  Emergency Room: $137.22 [€168.00]  Day Hospital: $892.69 [€1,092.96] |  | N/A |
| Lu 2020 | Societal, Health Care System  USD$, 2020, 1-year, N/A |  | Assisted services: $91 ($1817) | All Costs: $4,714 ($$27,796)  Rehabilitation: $215 ($1,846)  Psychiatric care: $148 ($969) |  | Did not adjust for previous diagnosis of depression or anxiety |

*Appendix Table 2 Continued*

| **Publication** | **Cost Perspectives** Currency, Year^1^, Time Period Costs Were Measured, Discounting | **Total Cost of Outcomes Related to Impaired or Loss of Functional Independence**  Mean (SD)^2^ | **Societal Costs**  Mean (SD)^2^ | **Health Care Costs**  Mean (SD)^2^ | **Personal Costs**  Mean (SD)^2^ | **Sensitivity Analysis** |
| --- | --- | --- | --- | --- | --- | --- |
| Mann 1999 | Assumed health care system  USD$, assumed 1995, 1-year, N/A |  |  | All Costs Intervention: $24,431.00 ($23,722.48) [$14,172 (13,761)] Control: $54,492.23 ($72,815.48) [$31,610 (42,239)]  Hospital Intervention: $8,614.29 ($19,995.43) [$4,997 (11,599)] Control: $35,901.78 ($70,336.53) [$20,826 (40,801)]  Outpatient Intervention - Nurse visits: $734.38 ($1,236.03) [$426 (717)] - Case manager visits: $189.63 ($282.72) [$110 (164)] - OT visits:$1,068.81 ($3,701.20) [$620 (2,147)] - PT visits: $2,037.64 ($4,544.18) [$1,182 (2,636)] - Aide hours: $6,180.15 ($9,850.32) [$3,585 (5,714)] Control  - Nurse visits: $1,451.52 ($2,501.37) [$842 (1,451)] - Case manager visits: $332.71 ($332.71) [$193 (138)] - OT visits: $1,582.53 ($4,713.12) [$918 (2,734)] - PT visits: $2,796.15 ($6,712.84) [$1,622 (3,894)] - Aide hours: $9,850.32 ($13,187.78) [$5,714 (7,650)] |  | N/A |

*Appendix Table 2 Continued*

| **Publication** | **Cost Perspectives** Currency, Year^1^, Time Period Costs Were Measured, Discounting | **Total Cost of Outcomes Related to Impaired or Loss of Functional Independence**  Mean (SD)^2^ | **Societal Costs**  Mean (SD)^2^ | **Health Care Costs**  Mean (SD)^2^ | **Personal Costs**  Mean (SD)^2^ | **Sensitivity Analysis** |
| --- | --- | --- | --- | --- | --- | --- |
| Marshall 1999 | Assumed Health Care System  USD$, 1992, 1-year, N/A |  |  | All Costs Intervention Base: $11,455.08 [$6,105.52] Year 1: $12,960.27 [$6,907.78] Year 2: $10,162.91 [$5,416.80] Control Base: $10,798.38 [$5,755.50] Year 1: $8,991.14 [$4,792.25] Year 2: $5,956.79 [$3,174.95]  Hospital Intervention Base: $6,077.73 [$3,239.41] Year 1: $6,872.54 [$3,663.04] Year 2: $4,267.77 [$2,274.71] Control Base: $10,798.38 [$5,755.50] Year 1: $8,991.14 [$4,792.25] Year 2: $5,956.79 [$3,174.95]  Outpatient Intervention Base: $3,885.63 [$2,071.03] Year 1: $3,524.15 [$1,878.36]  Year 2: $3,378.56 [$1,800.76] Control Base: $5,563.50 [$2,965.33] Year 1: $4,448.77 [$2,371.18]  Year 2: $2,251.42 [$1,200] |  | N/A |

*Appendix Table 2 Continued*

| **Publication** | **Cost Perspectives** Currency, Year^1^, Time Period Costs Were Measured, Discounting | **Total Cost of Outcomes Related to Impaired or Loss of Functional Independence**  Mean (SD)^2^ | **Societal Costs**  Mean (SD)^2^ | **Health Care Costs**  Mean (SD)^2^ | **Personal Costs**  Mean (SD)^2^ | **Sensitivity Analysis** |
| --- | --- | --- | --- | --- | --- | --- |
| Maru 2015 | Health Care System  AUD$, 2013, time horizon of the analysis was the combined follow-up periods (mean 2.7, median 3.2, maximum 4.8 years), discounting assumption was varied and the base-case (cost 5%, QALY 5%) was compared against two scenarios: undiscounted (cost 0%, QALY 0%) and discounting cost-only (cost 5%, QALY 0%) |  |  | All Costs Per 100 Days Home-Based Intervention: $4,885.11 ($7,535.65) [$6,283 (9,692)] Clinic-Based Intervention: $10,596.72 ($21,884.65) [$13,629 (28,147)]  Hospital Home-Based Intervention: $4,198.57 ($7,062.92) [$5,400 (9,084)] Clinic-Based Intervention: $9,634.16 ($21,184.89) [$12,391 (27,247)] | Medications Home-Based Intervention: $375.54 ($298.56) [$483 (384)] Clinic-Based Intervention: $492.17 ($562.14) [$633 (723)] | One way |
| Max 1995 | Personal  USD$, 1990, 1-year, N/A |  |  |  | Informal Care by Severity: Severe: $74,006.49 [$36,389] Mild/Moderate: $64,952.19 [$31,937] Total: $70,199.29 [$34,517] | N/A |

*Appendix Table 2 Continued*

| **Publication** | **Cost Perspectives** Currency, Year^1^, Time Period Costs Were Measured, Discounting | **Total Cost of Outcomes Related to Impaired or Loss of Functional Independence**  Mean (SD)^2^ | **Societal Costs**  Mean (SD)^2^ | **Health Care Costs**  Mean (SD)^2^ | **Personal Costs**  Mean (SD)^2^ | **Sensitivity Analysis** |
| --- | --- | --- | --- | --- | --- | --- |
| McCusker 2003 | Societal  CAD$, 1999, 4-month, N/A |  |  | All Public Health Care Resource Costs Intervention: $4,228.52 ($5,419.07) [$3,193 ($4,092)] Control: $4,733.08 ($5,372.72) [$3,574 ($4,057)]  All Private Health Care Resource Costs Intervention: $720.42 ($864.77) [$544 ($653)] Control: $727.05 ($950.85) [$549 ($718)]  Acute Hospitalization Intervention: $1,528.25 ($4,077.54) [$1,154 ($3,079)] Control: $1,905.68 ($3,837.85) [$1,439 ($2,898)] | Patient Costs Intervention: $332.40 ($443.64) [$251 ($335)] Control: $331.08 ($683.34) [$250 ($516)]  Caregiver Costs Intervention: $74.16 ($176.13) [$56 ($133)] Control: $56.95 ($239.70) [$43 ($181)]  Medications Intervention: $1,236.90 ($1,174.66) [$934 ($887)] Control: $1,018.39 ($773.40) [$769 ($584)] | N/A |
| McNamee 1999 | Assumed societal  Pounds Sterling £, 1994/5, 1-week, N/A |  | Costs Per Week Household Structure **-** Living alone: $82.79 [£70.57] - Living with others: $69.11 [£58.91] Frailty Type - Mentally & physically frail: $128.01 [£109.11] - Physically frail: $75.25 [£64.14] - Mentally frail: $46.11 [£39.30] Age - 65-75: $63.21 [£53.88] - 76-85: $77.06 [£65.68] - Over 85: $85.30 [£72.71] Sex - Female: $71.31 [£60.78] - Male: $85.97 [£73.28] |  |  | N/A |

*Appendix Table 2 Continued*

| **Publication** | **Cost Perspectives** Currency, Year^1^, Time Period Costs Were Measured, Discounting | **Total Cost of Outcomes Related to Impaired or Loss of Functional Independence**  Mean (SD)^2^ | **Societal Costs**  Mean (SD)^2^ | **Health Care Costs**  Mean (SD)^2^ | **Personal Costs**  Mean (SD)^2^ | **Sensitivity Analysis** |
| --- | --- | --- | --- | --- | --- | --- |
| Melin 1993 | Assumed Health Care System  Swedish Krona, 1989, 6-months, N/A |  |  | All Costs Treatment: $33,046.96 [130,000] Control: $41,181.60 [162,000]  Inpatient Treatment - Short term: $15,252.44 [60,000] - Long term: $5,846.77 [23,000] - Rehabilitation: $762.62 [3,000] Control - Short term: $15,506.65 [61,000] - Long term: $17,794.52 [70,000]  Rehabilitation: $1,016.83 [4,000]  Outpatient Treatment: $8,897.26 [35,000] Control: $5,846.77 [23,000]  Other Health care Treatment: $1,016.83 [4,000] Control: $762.62 [3,000] |  | N/A |

*Appendix Table 2 Continued*

| **Publication** | **Cost Perspectives** Currency, Year^1^, Time Period Costs Were Measured, Discounting | **Total Cost of Outcomes Related to Impaired or Loss of Functional Independence**  Mean (SD)^2^ | **Societal Costs**  Mean (SD)^2^ | **Health Care Costs**  Mean (SD)^2^ | **Personal Costs**  Mean (SD)^2^ | **Sensitivity Analysis** |
| --- | --- | --- | --- | --- | --- | --- |
| Metzelthin 2015 | Societal  Euros €, 2010, 2-years, N/A | Intervention: $24,237.30 ($24,941.48) [€26,503 (27,273)] Control: $18,793.21 ($17,276.04) [€20,550 (18,891)] | Home Health care Intervention: $7,344.44 ($9,567.62) [€8,031 (10,462)] Control: $6,419.87 ($6,834.15) [€7,020 (7,473)] | All Costs Intervention: $16,153.93 ($16,714.53) [€17,664 (18,277)] Control: $11,854.81 ($9,546.59) [€12,963 (10,439)]  Hospital Intervention: $2,656.66 ($6,293.67) [€2,905 (6,882)] Control: $1,360.79 ($2,043.93) [€1,488 (2,235)]  Allied Professionals Intervention: $1,087.35 ($1,754.03) [€1,189 (1,918)] Control: $1,208.98 ($1,771.41) [€1,322 (1,937)]  GP Care Intervention: $980.36 ($669.42) [€1,072 (732)] Control: $666.68 ($498.41) [€729 (545)] | All Costs Intervention: $8,083.37 ($16,880.97) [€8,839 (18,459)] Control: $6,938.40 ($13,279.62) [€7,587 (14,521)]  Medications Intervention: $2,933.75 ($5,766.91) [€3,208 (6,306)] Control: $1,995.46 ($2,021.99) [€2,182 (2,211)]  Informal Care Intervention: $7,578.56 ($16,783.12) [€8,287 (18,352)] Control: $6,684.17 ($13,251.27) [€7,309 (14,490)]  Aids/In-Home Modifications Intervention: $504.81 ($975.78) [€552 (1,067)] Control: $254.23 ($588.03) [€278 (643)] | One-way |
| Michalowsky 2016 | Societal  Euros €, 2014, 1-year, N/A |  | Home Health care: $927.85 ($2,382.51) [€1,136 (2,917)] |  | Informal Care: $13,454.62 ($13,914.46) [€16,473 (17,036)] | In the sensitivity analyses, three different alternative scenarios were calculated |

*Appendix Table 2 Continued*

| **Publication** | **Cost Perspectives** Currency, Year^1^, Time Period Costs Were Measured, Discounting | **Total Cost of Outcomes Related to Impaired or Loss of Functional Independence**  Mean (SD)^2^ | **Societal Costs**  Mean (SD)^2^ | **Health Care Costs**  Mean (SD)^2^ | **Personal Costs**  Mean (SD)^2^ | **Sensitivity Analysis** |
| --- | --- | --- | --- | --- | --- | --- |
| Miller 2005 | Assumed health care system  Pounds Sterling £, 2000, 1-year, N/A | Mean (SE) Intervention: $9,039.45 ($583.82) [£8,361 (£540)] Control: $10,906.59 ($770.86) [£10,088 (£713)] | Mean (SE)  Social Services Intervention: $713.56 ($101.63) [£660 (£94)] Control: $825.99 ($97.30) [£764 (£90)]  Community Health Services  Intervention: $435.70 ($73.52) [£403 (£68)] Control: $449.76 ($64.87) [£416 (£60)] | Mean (SE)  Initial Inpatient Admission Intervention: $2,400.14 (151.43482) [£2,220 (£215)] Control: $4,157.00 ($463.81) [£3,845 (£429)]  Inpatient Readmissions  Intervention: $3,356.96 ($404.35) [£3,105 (£374)] Control: $3,286.68 ($480.03) [£3,040 (£444)]  Early Discharge Rehabilitation Service (EDRS): Intervention: $551.38 ($40.00) [£510 (£37)] Control: $3.24 ($3.24) [£3 (£3)]  Outpatient Visits  Intervention: $230.28 ($33.52) [£213 (£31)] Control: $236.77 ($22.70) [£219 (£21)] |  | Probabilistic |
| Mintzer 1997 | Health Care System  USD$, assumed 1992, costs were measured over the period of each respective program (21-days & 7-days+(length of hospital stay-7)), N/A |  |  | All Costs Intervention: $34,818.22 ($10,938.15) [$18,558 ($5,830)] Control: $18,011.37 ($6,579.78) [$9,600 ($3,507)] |  | N/A |

*Appendix Table 2 Continued*

| **Publication** | **Cost Perspectives** Currency, Year^1^, Time Period Costs Were Measured, Discounting | **Total Cost of Outcomes Related to Impaired or Loss of Functional Independence**  Mean (SD)^2^ | **Societal Costs**  Mean (SD)^2^ | **Health Care Costs**  Mean (SD)^2^ | **Personal Costs**  Mean (SD)^2^ | **Sensitivity Analysis** |
| --- | --- | --- | --- | --- | --- | --- |
| Mitchell 2019 | Assumed Health Care System  AUD$, 2009, 1-year, N/A |  |  | All Costs: $32,384.97 [$38,041]  Hospital: $47,946.21 [$56,320]  Acute Care: $23,177.97 [$27,226]  Rehabilitation: $12,384.12 [$14,547]  Sub-Acute Care: $14,861.45 [$17,457] |  | N/A |
| Murray 2003 | Assumed health care system  USD$, 2002, 7-months, N/A |  |  | Hospital Costs by HARP Scores [Mean (Range)]  Score = 3: $9,119.01 ($987.54-$32,831.65) [$6,233 ($675-$22,441)] Score = 4: $8,775.20 ($1,032.89-$35,722.58) [$5,998 ($706-$24,417)] Score = 5: $7,211.23 ($7,307.79-$22,534.91) [$4,929 ($4,995-$15,403)]  Hospital Costs by Discharge Disposition [Mean (Range)] Routine discharge to home: $6,068.61 [$4,148] Discharge home with home care: $8,210.47 [$5,612] |  | N/A |
| Nikolaus 1999 | Assumed health care system  USD$, assumed 1995, 1-year, N/A  *Costs reported per 100 people per year* | Intervention: $3,314,010.40 [$1,922,400] Assessment: $3,924,613.02 [$2,276,600] Control: $4,082,693.93 [$2,368,300] | Assisted Services Intervention: $325,126.07 [$188,600] Assessment: $246,344.20 [$142,900] Control: $206,867.07 [$120,000] | Hospital **Initial** - Intervention: $1,715,272.75 [$995,000] - Assessment: $2,084,185.69 [$1,209,000] - Control: $2,189,343.11 [$1,270,000] **Readmissions**  - Intervention: $659,043.99 [$382,300] - Assessment: $985,032.01 [$571,400] - Control: $1,010,718.01 [$586,300]  Physician Visits - Home Intervention: $87,746.11 [$50,900] - Assessment: $78,954.26 [$45,800] - Control: $76,885.59 [$44,600]  Medical Staff - Intervention: $236,173.23 [$137,000] - Assessment: $49,303.32 [$28,600] |  | N/A |

*Appendix Table 2 Continued*

| **Publication** | **Cost Perspectives** Currency, Year^1^, Time Period Costs Were Measured, Discounting | **Total Cost of Outcomes Related to Impaired or Loss of Functional Independence**  Mean (SD)^2^ | **Societal Costs**  Mean (SD)^2^ | **Health Care Costs**  Mean (SD)^2^ | **Personal Costs**  Mean (SD)^2^ | **Sensitivity Analysis** |
| --- | --- | --- | --- | --- | --- | --- |
| Pinedo‑Villanueva 2019 | Health Care System  Pounds Sterling £, 2015, 1 year, N/A | W/out muscle weakness: $1,446.92 ($134.33) [£1,885 (£175)] W/ Muscle Weakness: $3,524.80 ($621.75) [£4,592 (£810)] |  | Primary Care Without muscle weakness: $333.14 ($23.80) [£434 (31)] W/ muscle weakness: $674.72 ($156.59) [£879 (204)]  Outpatient Without muscle weakness: $76.76 ($7.68) [£100 (10)] W/ muscle weakness: $105.16 ($33.01) [£137 (43)]  Inpatient Without muscle weakness: $196.50 ($42.99) [£256 (£56)] W/ muscle weakness: $792.93 ($287.85) [£1,033 (£375)]  Formal Care Without muscle weakness: $295.52 ($69.08) [£385 (£90)] W/ muscle weakness: $323.16 ($109.00) [£421 (£142)] | Medications Without muscle weakness: $167.34 ($22.26) [£218 (£29)] W/ muscle weakness: $297.83 ($143.54) [£388 (£187)]  Informal Care Without muscle weakness: $377.66 ($72.92) [£492 (£95)] W/ muscle weakness: $1,331.01 ($297.83) [£1,734 (£388)] | N/A |

*Appendix Table 2 Continued*

| **Publication** | **Cost Perspectives** Currency, Year^1^, Time Period Costs Were Measured, Discounting | **Total Cost of Outcomes Related to Impaired or Loss of Functional Independence**  Mean (SD)^2^ | **Societal Costs**  Mean (SD)^2^ | **Health Care Costs**  Mean (SD)^2^ | **Personal Costs**  Mean (SD)^2^ | **Sensitivity Analysis** |
| --- | --- | --- | --- | --- | --- | --- |
| Pitkala 2013 | Assumed societal & health care system  USD$, 2012, 2-years, N/A | All Costs Intervention 1: $3,720,478.43 [$3,254,724] Intervention 2: $3,248,450.85 [$2,841,788] Control: $4,297,834.77 [$3,759,803]  All Costs + Intervention Intervention 1: $4,370,214.78 [$3,823,122] Intervention 2: $4,028,411.56 [$3,524,108] Control: $4,297,834.77 [$3,759,803] | Home Health care - Intervention 1: $307.49 [$269] - Intervention 2: $5,228.54 [$4,574] - Control: $10,148.45 [$8,878]  Day Care - Intervention 1: $61,981.23 [$54,222] - Intervention 2: $13,205.10 [$11,552] - Control: $14,283.05 [$12,495]  Respite Care - Intervention 1: $654,795.72 [$572,824] - Intervention 2: $571,411.07 [$499,878] - Control: $597,500.06 [$522,701] | Primary Care Hospitals - Intervention 1: $453,900.28 [$397,078] - Intervention 2: $271,532.22 [$237,540] - Control: $262,448.00 [$229,593]  Specialized Care Hospitals - Intervention 1: $1,215,718.83 [$1,063,527] - Intervention 2: $1,146,786.41 [$1,003,224] - Control: $1,909,744.63 [$1,670,670]  Physician Visits - Intervention 1: $101,489.08 [$88,784] - Intervention 2: $121,385.90 [$106,190] - Control: $117,165.57 [$102,498]  Physician Home Visits - Intervention 1: $9,445.44 [$8,263] - Intervention 2: $7,435.87 [$6,505] - Control: $10,249.04[$8,966]  Outpatient - Intervention 1: $60,564.92 [$52,983] - Intervention 2: $57,536.85 [$50,334] - Control: $58,546.21 [$51,217]  Primary Care Nurse Visits - Intervention 1: $47,258.08 [$41,342] - Intervention 2: $55,784.47 [$48,801] - Control: $53,957.80 [$47,203]  Physiotherapist Visits - Intervention 1: $55,418.68 [$48,481] - Intervention 2: $26,125.57 [$22,855] - Control: $73,469.39 [$64,272] | Home Help (Indirect Costs) - Intervention 1: $108,157.94 [$94,618] - Intervention 2: $103,846.16 [$90,846] - Control: $287,861.42 [$251,825] | N/A |

*Appendix Table 2 Continued*

| **Publication** | **Cost Perspectives** Currency, Year^1^, Time Period Costs Were Measured, Discounting | **Total Cost of Outcomes Related to Impaired or Loss of Functional Independence**  Mean (SD)^2^ | **Societal Costs**  Mean (SD)^2^ | **Health Care Costs**  Mean (SD)^2^ | **Personal Costs**  Mean (SD)^2^ | **Sensitivity Analysis** |
| --- | --- | --- | --- | --- | --- | --- |
| Reuben 2004 | Assumed Health Care System  USD$ , 1981, 1-year;4-years, N/A |  | 1-Year Hospital Costs by SPPB Score: - 10-12: $4,467.26 [$1,500] - 7–9: $7,147.61 [$2,400] - 4-6: $9,530.15 [$3,200] - 0-3: $14,295.23 [$4,800]  4-Year Hospital Costs by SPPB Score: - 10-12: $25,910.10 [$8,700] - 7–9: $32,164.26 [$10,800] - 4-6: $46,459.49 [$15,600] - 0-3: $58,967.81 [$19,800] |  |  | N/A |
| Rigaud 2003 | Assumed Health Care System  Euros €, 1991, 1-month, N/A | Total Costs in Past 6-Months By MMSE Score ≤ 10: $8,388.67 [€4,438.4] 11–15: $3,123.45 [€1,652.6] 16–20: $1,875.09 [€992.1] > 21: $992.64 [€525.2] |  | All Costs ≤ 10: $988.29 [€522.9] 11–15: $817.06 [€432.3] 16–20: $709.89 [€375.6] > 21: $560.20 [€296.4] | Informal Care ≤ 10: $5,698.42 [€3,015.0] 11–15: $1,776.05 [€939.7] 16–20: $897.19 [€474.7] > 21: $333.02 [€176.2]  Paid Assistance ≤ 10: $988.29 [€522.9] 11–15: $817.06 [€432.3] 16–20: $709.89 [€375.6] > 21: $560.20 [€296.4] | N/A |

*Appendix Table 2 Continued*

| **Publication** | **Cost Perspectives** Currency, Year^1^, Time Period Costs Were Measured, Discounting | **Total Cost of Outcomes Related to Impaired or Loss of Functional Independence**  Mean (SD)^2^ | **Societal Costs**  Mean (SD)^2^ | **Health Care Costs**  Mean (SD)^2^ | **Personal Costs**  Mean (SD)^2^ | **Sensitivity Analysis** |
| --- | --- | --- | --- | --- | --- | --- |
| Rojas 2010 | Assumed health care system  USD$, 2008, 3-months, N/A |  |  | All Costs According to Diagnosis Healthy Subjects: $182.39 ($174.06) [$148.59 (141.8)] DAT: $1,419.34 ($1,910.61) [$1,156.28 (1,556.5)] FTD: $1,511.10 (1,425.5) [$1,231.04 (1,425.5)] VaD: $1,568.87 ($1,766.01) [$1,278.1 (1,438.7)]  Hospital Healthy Subject: $0 (0) [$0 (0)] DAT: $395.26 ($1,396.90) [$322.00 (1,138.0)] FTD: $440.18 ($1,396.90) [$358.60 (1,138.0)] VaD: $649.47 ($1,428.81) [$529.10 (1,164)]  Physician’s Visits Healthy Subjects: $34.74 ($34.74) [$28.30 (28.3)] DAT: $49.71 ($79.54) [$40.50 (64.8)] FTD: $36.70 ($44.56) [$29.90 (36.3)] VaD: $32.28 ($42.35) [$26.30 (34.5)]  Day Hospital Healthy Subject: $17.06 ($91.08) [$13.90 (74.2)] DAT: $342.47 ($629.59) [$279.00 (512.9)] FTD: $259.86 ($569.93) [$211.70 (464.3)] VaD: $207.69 ($484.49) [$169.20 (394.7)] | Medications Healthy Subject: $37.44 ($37.56) [$30.50 (30.6)] DAT: $220.58 ($651.80) [$179.70 (531)] FTD: $237.52 ($217.27) [$193.50 (177)] VaD: $177.50 ($252.01) [$144.60 (205.3)] | N/A |

*Appendix Table 2 Continued*

| **Publication** | **Cost Perspectives** Currency, Year^1^, Time Period Costs Were Measured, Discounting | **Total Cost of Outcomes Related to Impaired or Loss of Functional Independence**  Mean (SD)^2^ | **Societal Costs**  Mean (SD)^2^ | **Health Care Costs**  Mean (SD)^2^ | **Personal Costs**  Mean (SD)^2^ | **Sensitivity Analysis** |
| --- | --- | --- | --- | --- | --- | --- |
| Ruchlin 2001 | Societal  USD$, 1995, 18-months, all costs and beneﬁts occurring after the ﬁrst year were discounted at 3% | Intervention: $23,862.12 ($20,585.00) [$13,842 (11,941)] Control: $29,545.79 ($37,196.42) [$17,139 (21,577)] |  | All Costs Intervention: $18,824.90 ($16,735.55) [$10,920 (9,708)] Control: $20,778.07 (15,183) [$12,053 (15,183)]  Formal Help Intervention: $4,418.34 ($11,272.53) [$2,563 ($6,539)] Control: $7,555.82 ($16,471.79) [$4,383 ($9,555)] | Informal Care Intervention: $468.90 ($1,272.23) [$272 (738)] Control: $1,018.82 ($4,442.47) [$591 (2,577)] | N/A |
| Ruikes 2018 | Health Care System  Euros €, 2011, 1-year, N/A | Intervention: $9,389.20 ($872.69) [€10,576 (983)] Control: $7,203.48 ($7,203.48) [€8,114 (845)] | Day Care Intervention: $374.64 ($90.55) [€422 (102)] Control: $303.62 ($89.67) [€342 (101)]  Home Health care Intervention: $3,295.45 ($375.53) [€3,712 (423)] Control: $2,474.25 ($365.77) [€2,787 (412)] | Hospital Intervention: $1,382.28 ($452.77) [€1,557 (510)] Control: $1,087.53 ($220.17) [€1,225 (248)]  Outpatient Intervention: $212.18 ($21.31) [€239 (24)] Control: $269.89 ($35.51) [€304 (40)]  GP Visits Intervention: $144.71 ($11.54) [€163 (13)] Control: $150.04 ($15.98) [€169 (18)]  Physiotherapy Intervention: $877.13 ($274.33) [€988 (309)] Control: $430.58 ($77.24) [€485 (87)] | Medications Intervention: $1,435.55 ($262.78) [€1,617 (296)] Control: $868.25 ($111.86) [€978 (126)]  Home Help (Indirect Costs) Intervention: $1,306.82 ($80.79) [€1,472 (91)] Control: $1,257.99 ($100.32) [€1,417 (113)] | Performed sensitivity analysis without medication costs |

*Appendix Table 2 Continued*

| **Publication** | **Cost Perspectives** Currency, Year^1^, Time Period Costs Were Measured, Discounting | **Total Cost of Outcomes Related to Impaired or Loss of Functional Independence**  Mean (SD)^2^ | **Societal Costs**  Mean (SD)^2^ | **Health Care Costs**  Mean (SD)^2^ | **Personal Costs**  Mean (SD)^2^ | **Sensitivity Analysis** |
| --- | --- | --- | --- | --- | --- | --- |
| Schnaider-Beeri 2002a | Assumed Personal  USD$, 1999, 1-year, N/A |  |  | Clinical: $41.63/yr [$26.40/yr] | All Costs: $22,740.33 [$14,420]  Indirect: $16,590.03 [$10,520]  Direct Health-Care Related: $6,150.30 [$3,900]  Medications: $791.02/yr [$501.60/yr]  Home Help (Indirect): $5,317.64/yr [$3,372/yr] | N/A |
| Schnaider-Beeri, 2002b | Assumed Personal  USD$, 1999, 6 months, N/A | $27331/yr [$17730/yr] | Assisted Services: $4793/yr [$2268/yr]  Home Healthcare: $6492/yr [$3372/yr] | All: $10837/yr [$7030/yr]  Clinician: $139/yr [$90/yr] | All: $8124/yr [$5270/yr]  Home Help (Indirect): $6493/yr [$4212/yr]  Direct Health-Care Related: $536/yr [$348/yr]  Medications: $1095/yr [$710/yr] | N/A |

*Appendix Table 2 Continued*

| **Publication** | **Cost Perspectives** Currency, Year^1^, Time Period Costs Were Measured, Discounting | **Total Cost of Outcomes Related to Impaired or Loss of Functional Independence**  Mean (SD)^2^ | **Societal Costs**  Mean (SD)^2^ | **Health Care Costs**  Mean (SD)^2^ | **Personal Costs**  Mean (SD)^2^ | **Sensitivity Analysis** |
| --- | --- | --- | --- | --- | --- | --- |
| Schousboe 2019 | Assumed Health Care System  USD$, 2017, 1-year, N/A |  |  | All Costs by GDS Score 0-1: $4,965.66 ($10,331.13) [$4,654 ($9,682.72)] 2-5: $8,398.09 ($15,507.29) [$7,871 (14,534)] 6 or more: $9,613.37 ($16,621.20) [$9,010 (15,578)]  Outpatient 0-1: $2,014.43 ($2,576.72) [$1,888 (2,415)] 2-5: $2,643.94 ($3,524.19) [$2,478 (3,303)] 6 or more: $2,895.75 ($3,669.30) [$2,714 (3,439)] |  | N/A |
| Schraeder 2008 | Assumed Health Care System  USD$, not reported, 1-month, N/A |  |  | All Costs: $1,464.41 ($2,397.31) [$1,193 ($1,953)] |  | N/A |
| Schwarzkopf 2011 | Societal  Euros €, 2008, 1-year, N/A | $46,364.45 [€47,792] |  | Formal Care: $9,339.44 [€9,627]  Hospital: $3,797.09 [€3,914]  Non-Physician: $262.91 [€271]  Rehabilitation: $206.64 [€213]  Physician Visits: $934.24 [€963] | Medications: $1,560.94 [€1,609]  Informal Care: $37,025.01 [€38,165]  Medical aids and assistive devices: $312.38 [€322] | N/A |

*Appendix Table 2 Continued*

| **Publication** | **Cost Perspectives** Currency, Year^1^, Time Period Costs Were Measured, Discounting | **Total Cost of Outcomes Related to Impaired or Loss of Functional Independence**  Mean (SD)^2^ | **Societal Costs**  Mean (SD)^2^ | **Health Care Costs**  Mean (SD)^2^ | **Personal Costs**  Mean (SD)^2^ | **Sensitivity Analysis** |
| --- | --- | --- | --- | --- | --- | --- |
| Scott 2004 | Assumed health care system  Assumed USD$, 1995, 2-years, N/A |  | Home Health care Intervention: $363.74 ($1,175.69) [$211 (682)] Control: $675.77 ($1,596.32) [$392 (926)] | All Costs Intervention: $13,646.33 ($16,182.18) [$7,916 (9,387)] Control: $1,922.14 ($29,369.95) [$1,115 (17,037)]  Hospital Intervention: $3,690.85 ($8,478.10) [$2,141 (4,918)] Control: $7,640.29 ($20,166.09) [$4,432 (11,698)]  Professional Services Intervention: $1,206.72 ($2,111.77) [$700 (1,225)] Control: $2,285.88 ($4,120.10) [$1,326 (2,390)]  CHCC Clinic Intervention: $3,696.02 ($3,161.62) [$2,144(1,834)] Control: $3,666.72 ($3,054.74) [$2,127 (1,772)]  Outpatient Intervention: $956.76 ($4,980.32) [$555 (2,889)] Control: $858.50 ($2,622.04) [$498 (1,521)] | Medications Intervention: $1,144.66 ($1,308.43) [$664 (759)] Control: $1,382.56 ($2,101.42) [$802 (1,219)] | N/A |

*Appendix Table 2 Continued*

| **Publication** | **Cost Perspectives** Currency, Year^1^, Time Period Costs Were Measured, Discounting | **Total Cost of Outcomes Related to Impaired or Loss of Functional Independence**  Mean (SD)^2^ | **Societal Costs**  Mean (SD)^2^ | **Health Care Costs**  Mean (SD)^2^ | **Personal Costs**  Mean (SD)^2^ | **Sensitivity Analysis** |
| --- | --- | --- | --- | --- | --- | --- |
| Taylor 2001 | Health Care System, Personal  USD$, 1994, 1-year, N/A | By Alzheimer’s Disease and Related Dementias (ADRD) Severity Severe ADRD: $31,928.62 [$18,016] Moderate ADRD: $18,367.46 [$10,364] |  | All Costs Severe ADRD: $9,499.19 [$5,360] Moderate ADRD: $7,664.92 [$4,325] | Medications Severe ADRD: $1,213.98 [$685] Moderate ADRD: $1,291.96 [$729]  Caregiving for ADLs Severe ADRD: $9,041.95 [$5,102] Moderate ADRD: $3,540.93 [$1,998]  Caregiving for IADLs Severe ADRD: $11,402.57 [$6,434] Moderate ADRD: $5,169.61 [$2,917] | N/A |
| Van Lier 2016 | Societal and Health Care System  Euros €, 2015, 3-months, N/A |  | All Costs Assessed with RUD Lite: $5,246.02 ($6,851.08) [€6,295 (8,221)] Assessed with interRAI-HC: $5,916.05 ($6,190.22) [€7,099 (7,428)]  Meals-on-Wheels Assessed with RUD Lite: $147.51 ($230.84) [€177 (277)] Assessed with interRAI-HC: $185.01 ($232.51) [€222 (279)]  Home Health care Assessed with RUD Lite: $1,974.24 ($2,383.42) [€2,369 (2,860)] Assessed with interRAI-HC: $2,169.24 ($2,087.58) [€2,603 (2,505)] | Hospital Assessed with RUD Lite: $566.69 ($2,140.08) [€680 (2,568)] Assessed with interRAI-HC: $997.54 ($3,114.28) [€1,197 (3,737)]  Physician Visits Assessed with RUD Lite: $69.17 ($131.67) [€83 (158)] Assessed with interRAI-HC: $77.50 ($148.34) [€93 (178)] | Informal Care Assessed with RUD Lite: $2,397.59 ($5,641.87) [€2,877 (6,770)] Assessed with interRAI-HC: $2,388.42 ($4,822.67) [€2,866 (5,787)] | One-way |

*Appendix Table 2 Continued*

| **Publication** | **Cost Perspectives** Currency, Year^1^, Time Period Costs Were Measured, Discounting | **Total Cost of Outcomes Related to Impaired or Loss of Functional Independence**  Mean (SD)^2^ | **Societal Costs**  Mean (SD)^2^ | **Health Care Costs**  Mean (SD)^2^ | **Personal Costs**  Mean (SD)^2^ | **Sensitivity Analysis** |
| --- | --- | --- | --- | --- | --- | --- |
| Wang 2008 | Assumed health care system  Chinese Yuan Renminbi ¥, 2006, 1-year, N/A | Total AD-Related Costs: $8,656.76 ($5,028.40) [¥19,001 (11,037)]  **Comparison of Overall Mean Costs for AD Per Patient by Country** - China, 2007: $1,086.14 [$2,384] - Turkey, 2005: $804.58-$2,246.08 [$1,766–4,930] - Argentina, 2007: $2,406.00-$5,121.80 [$5,281–11,242] - Spain, 2007: $16,467.02 [$36,144] - USA, 2006: $11,688.74 [$25,656] - North Europe, 2006: $9,693.24 [$21,276] |  | Direct Medical Costs: $2,569.56 ($2,252.46) [¥5,640 (4,944)]  Direct Non-Medical Costs: $1,272.02 ($1,001.85) [¥2,792 (2,199)]  Formal Care: $1,100.72 ($798.66) [¥2,416 (1,753)] | Medications: $2,370.00 ($2,235.15) [¥5,202 (4,906)]  Indirect Costs*: $4,814.73 ($3,739.98) [¥10,568 (8,209)]  Informal Care: $4,814.73 ($3,739.98) [¥10,568 (8,209)]  Transportation: $91.57 ($66.52) [¥201 (146)]  **"Indirect costs refer to those resources for loss due to the illness, including premature deaths, patient and care-giver lost productivity, and unpaid caregiving time"* | N/A |

*Appendix Table 2 Continued*

| **Publication** | **Cost Perspectives** Currency, Year^1^, Time Period Costs Were Measured, Discounting | **Total Cost of Outcomes Related to Impaired or Loss of Functional Independence**  Mean (SD)^2^ | **Societal Costs**  Mean (SD)^2^ | **Health Care Costs**  Mean (SD)^2^ | **Personal Costs**  Mean (SD)^2^ | **Sensitivity Analysis** |
| --- | --- | --- | --- | --- | --- | --- |
| Wang 2010 | Assumed Personal  USD$, 2008, 1-year, N/A |  |  |  | Annual Cost of Informal Care: Personal ADL: - Mild: $1,108.43 ($3,141.18) [$903 (2,559)] - Moderate: $3,267.61 ($4,566.31) [$2,662 (3,720)] - Severe: $7,682.93 ($9,263.95) [$6,259 (7,547)] - Total: $3,342.49 ($5,469.75) [$2,723 (4,456)]  IADL: - Mild: $4,961.56 ($10,884.26) [$4,042 (8,867)] - Moderate: $4,072.85 ($6,682.52) [$3,318 (5,444)] - Severe: $9,384.25 ($10,099.88) [$7,645 (8,228)] - Total: $5,046.26 ($8,482.03) [$4,111 (6,910)]  Supervision: - Mild: $1,069.15 ($3,151.00) [$871 (2,567)] - Moderate: $3,549.93 ($6,564.68) [$2,892 (5,348)] - Severe: $7,576.14 ($9,452.99) [$6,172 (7,701)] - Total: $3,487.33 ($6,607.64) [$2,841 (5,383)] | Sensitivity analysis conducted to understand the distribution of informal care cost among six administrative regions |

*Appendix Table 2 Continued*

| **Publication** | **Cost Perspectives** Currency, Year^1^, Time Period Costs Were Measured, Discounting | **Total Cost of Outcomes Related to Impaired or Loss of Functional Independence**  Mean (SD)^2^ | **Societal Costs**  Mean (SD)^2^ | **Health Care Costs**  Mean (SD)^2^ | **Personal Costs**  Mean (SD)^2^ | **Sensitivity Analysis** |
| --- | --- | --- | --- | --- | --- | --- |
| Witham 2012 | Assumed health care system  Pounds Sterling £, assumed 2010, 24-week, N/A |  |  | All Costs Exercise: $1,584.49 ($2,610.56) [£1,888.24 (3,111)] Control: $1,631.23 ($3,818.92) [£1,943.93 (4,551)]  Inpatient Exercise: $662.70 ($2,232.11) [£789.74 (2,660)] Control: $1,089.61 ($3,818.92) [£1,298.49 (4,551)]   Outpatient Exercise: $99.12 ($97.34) [£118.12 (116)] Control: $112.77 ($111.61) [£134.39 (133)]  GP Visits Exercise: $109.99 ($108.25) [£131.08 (129)] Control: $147.30 ($151.88) [£175.54 (181)] | Medications Exercise: $249.29 ($420.41) [£297.08 (501)] Control: $297.58 ($160.28) [£248.87 (191)] | One-way, with and without data imputation |

*Appendix Table 2 Continued*

| **Publication** | **Cost Perspectives** Currency, Year^1^, Time Period Costs Were Measured, Discounting | **Total Cost of Outcomes Related to Impaired or Loss of Functional Independence**  Mean (SD)^2^ | **Societal Costs**  Mean (SD)^2^ | **Health Care Costs**  Mean (SD)^2^ | **Personal Costs**  Mean (SD)^2^ | **Sensitivity Analysis** |
| --- | --- | --- | --- | --- | --- | --- |
| Wolff 2019 | Assumed Health Care System   USD$, 2015, 1-year, N/A  *Costs reported as median (95% CI) and by disability and experience of negative consequences due to inadequate support* |  | Home Health care  **Household Disability** - No negative consequences: $4,809.36 ($4,197.38-$5,420.23) [$4,338 (3,786-4,889)] - Negative consequences: $4,570.99 ($3,352.58-$5,789.41) [$4,123 (3,024-5,222)]  **Mobility Disability** - No negative consequences: $4,174.10 ($3,663.00-$4,684.08) [$3,765 (3,304-4,225)] - Negative consequences: $5,528.87 ($4,408.02-$6,649.73) [$4,987 (3,976-5,998)]  **Self-Care Disability** - No negative consequences: $4,463.45 ($3,882.52-$5,066.56) [$4,026 (3,502-4,570)] - Negative consequences: $7,051.06 ($5,811.58-$8,291.65) [$6,360 (5,242-7,479)] | All Costs  **Household Disability** - None: $2,191.82 [$1,977] - No negative consequences: $4,539.95 [$4,095] - Negative consequences: $5,394.73 [$4,866] **Mobility Disability** - None: $2,219.53 [$2,002] - No negative consequences: $4,562.13 [$4,115] - Negative consequences: $8,055.50 [$7,266] **Self-Care Disability** - None: $2,338.16 [$2,109] - No negative consequences: $4,918.00 [$4,436] - Negative consequences: $12,123.17 [$10,935]  Clinician Services + Test **Household Disability** - No negative consequences: $2,342.59 ($2,176.29-$2,508.89) [$2,113 (1,963-2,263)] - Negative consequences: $3,076.52 ($2,318.20-$4,833.75) [$2,775 (2,091-4,360)] **Mobility Disability** - No negative consequences: $2,511.11 ($2,189.60-$2,832.62) [$2,265 (1,975-2,555)] - Negative consequences: $2,743.93 ($2,190.71-$3,297.15) [$2,475 (1,976-2,974)] **Self-Care Disability** - No negative consequences: $2,445.70 ($2,186.27-$2,705.12) [$2,206 (1,972-2,440)] - Negative consequences: $2,908.01 ($2,248.36-$3,457.90) [$2,623 (2,028-3,119)]  Outpatient **Household Disability** - No negative consequences: $1,126.40 ($940.14-$1,312.65) [$1,016 (848-1,184)] - Negative consequences: $1,259.43 ($829.28-$1,689.59) [$1,136 (748-1,524)] **Mobility Disability** - No negative consequences: $1,192.92 ($985.60-$1,401.34) [$1,076 (889-1,264)] - Negative consequences: $1,411.32 ($1,015.53-$1,806.00) [$1,273 (916-1,629)] **Self-Care Disability** - No negative consequences: $1,067.64 ($1,054.33-$1,413.54) [$963 (651-1,275)] - Negative consequences: $1,321.52 ($929.05-$1,715.09) [$1,192 (838-1,547)] |  | Authors assessed the effects of exclusion criteria by conducting separate sensitivity analyses with participants who did not survive the observation period or lived in a residential care facility but otherwise met eligibility criteria |

*Appendix Table 2 Continued*

| **Publication** | **Cost Perspectives** Currency, Year^1^, Time Period Costs Were Measured, Discounting | **Total Cost of Outcomes Related to Impaired or Loss of Functional Independence**  Mean (SD)^2^ | **Societal Costs**  Mean (SD)^2^ | **Health Care Costs**  Mean (SD)^2^ | **Personal Costs**  Mean (SD)^2^ | **Sensitivity Analysis** |
| --- | --- | --- | --- | --- | --- | --- |
| Wolstenholme 2002 | Assumed Health Care System  Pounds Sterling £, 1998, 40-months; 1-year, N/A | All Costs Over 40-months: $77,073.45 [£66,697]  Mean (95% CI)  Annual Cost of Care  **By Barthel Index** Mild: $9,343.99 ($228.80-$10,371.29) [£8,086 (198-8,975)] Mild/Moderate: $14,735.91 ($13,039.52-$16,432.29) [£12,752 (11,284-14,220)] Moderate: $26,855.59 ($22,726.71-$30,985.62) [£23,240 (19,667-26,814)] Severe: $27,174.53 ($24,344.52-$30,003.38) [£23,516 (21,067-25,964)] **By MMSE** Mild: $9,605.15 ($8,026.63-$11,703.67) [£8,312 (6,946-10,128)] Mild/Moderate: $13,454.37 ($11,143.22-$15,764.37) [£11,643 (9,643-13,642)] Moderate: $18,120.59 ($15,481.25-$20,761.08) [£15,681 (13,397-17,966)] Severe: $25,731.21 ($23,351.88-$28,111.70) [£22,267 (20,208-24,327)] |  |  |  | N/A |
| Woods 2012 | Societal  Pounds Sterling £, 2010, 10-month, N/A |  | Community Care  Intervention: $899.56 ($1,518.00) [£1,072 (1,809)]  Control: $981.79 ($1,664.01) [£1,170 (1,983)]  Day Care  Intervention: $921.37 ($3,735.00) [£1,098 (4,451)]  Control: $511.87 ($1,187.38) [£610 (1,415)] | Hospital Use  Intervention: $2,281.62 ($5,962.92) [£2,719 (7,106)]  Control: $2,122.18 (5675.246338) [£2,529 (8,087)] |  | N/A |

*Appendix Table 2 Continued*

| **Publication** | **Cost Perspectives** Currency, Year^1^, Time Period Costs Were Measured, Discounting | **Total Cost of Outcomes Related to Impaired or Loss of Functional Independence**  Mean (SD)^2^ | **Societal Costs**  Mean (SD)^2^ | **Health Care Costs**  Mean (SD)^2^ | **Personal Costs**  Mean (SD)^2^ | **Sensitivity Analysis** |
| --- | --- | --- | --- | --- | --- | --- |
| Wübker 2014 | Societal  Euros €, 2010, 1-month, N/A | Total Societal Costs* of Care for Each Country Stratified by Dementia Severity Group in a Home Care Setting (Mean [95% CI])  **Estonia: -** Mild, n=23: $463.84 ($317.34-$609.98) [€507.20 (347-667)] - Moderate, n=72: $657.44 ($506.64-$807.51) [€718.90 (554-883)] - Severe, n=68: $805.32 ($660.28-$951.09) [€880.60 (722-1,040)] **Finland: -** Mild, n=70: $2,204.89 ($1,496.14-$2,914.55) [€2,411 (1,636-3,187)] - Moderate, n=71: $1,846.40 ($1,420.24-$2,272.56) [€2,019 (1,553-2,485)] - Severe, n=18: $4,187.55 ($1,497.06-$6,877.13) [€4,579 (1,637-7,520)] **France: -** Mild, n=39: $2,073.20 ($1,636.06-$2,509.42) [€2,267 (1,789-2,744)] - Moderate, n=79: $2,786.52 ($2,364.01-$3,209.02) [€3,047 (2,585-3,509)] - Severe, n=32: $3,289.50 ($2,693.24-$3,886.67) [€3,597 (2,945-4,250)] **Germany: -** Mild, n=25: $2,032.04 ($1,563.81-$2,500.27) [€2,222 (1,710-2,734)] - Moderate, n=30: $2,967.59 ($2,214.03-$3,654.39) [€3,245 (2,421-3,996)] - Severe, n=36: $3,050.81 ($2,451.81-$3,679.99) [€3,336 (2,681-4,024)] **The Netherlands: -** Mild, n=54: $1,861.03 ($1,593.99-$2,961.19) [€2,035 (1,743-3,238)] - Moderate, n=72: $2,311.89 ($1,915.90-$2,706.95) [€2,528 (2,095-2,960)] - Severe, n=34: $2,550.57 ($2,043.02-$3,057.21) [€2,789 (2,234-3,343)] **Spain: -** Mild, n=57: $1,487.91 ($1,154.11-$1,820.79) [€1,627 (1,262-1,991)] - Moderate, n=63: $1,773.24 ($1,503.46-$2,043.93) [€1,939 (1,644-2,235)] - Severe, n=16: $2,274.39 ($1,565.64-$2,984.05) [€2,487 (1,712-3,263)] **Sweden: -** Mild, n=54: $1,692.76 ($1,463.22-$1,921.39) [€1,851 (1,600-2,101)] - Moderate, n=50: $1,990.89 ($1,678.13-$2,301.83) [€2,177 (1,835-2,517)] - Severe, n=25: $2,420.71 ($1,978.09-$2,863.34) [€2,647 (2,163-3,131)] **England: -** Mild, n=18: $1,668.98 ($1,072.72-$2,266.16) [€1,825 (1,173-2,478)] - Moderate, n=27: $2,727.99 ($1,850.06-$3,605.00) [€2,983 (2,023-3,942)] - Severe, n=15: $2,250.61 ($1,631.49-$2,870.65) [€2,461 (1,784-3,139)]  **Total costs consist of informal care, medical care, and community care costs.* |  |  |  | One-way, controlling for informal caregiving wage |

*Appendix Table 2 Continued*

| **Publication** | **Cost Perspectives** Currency, Year^1^, Time Period Costs Were Measured, Discounting | **Total Cost of Outcomes Related to Impaired or Loss of Functional Independence**  Mean (SD)^2^ | **Societal Costs**  Mean (SD)^2^ | **Health Care Costs**  Mean (SD)^2^ | **Personal Costs**  Mean (SD)^2^ | **Sensitivity Analysis** |
| --- | --- | --- | --- | --- | --- | --- |
| Zhang 2003 | Assumed Health Care System  USD$, 1996, not reported, N/A | $27,712.40 [$16,514] | *Costs reported as mean (25th percentile, 75th percentile)*  Home Health care: $4,178.51 ($0, $3,008.86) [$2,490 (0, 1,793)] | *Costs reported as mean (25th percentile, 75th percentile)*  Hospital: $13,894.79 ($0, $18,494.51) [$8,280 (0, 11,021)]  Outpatient: $1,508.63 ($0, $1,337.46) [$899 (0, 797)]  Health Care Professional Services: $5,339.76 ($1,391.16, $6,856.78) [$3,182 (829, 4,086)] |  | N/A |

^1^ For studies that did not explicitly state year of currency used, the year of currency was assumed to be the same as the study base year.

^2^ Costs reported as Mean (SD) unless otherwise stated; non-adjusted costs in square brackets

**Appendix Table 3.** Mean and standard deviations of annual costs per person of functional independence limitations based on each cost perspective

| **Cost Perspective** | **n_studies_** | **Mean Cost**  **(95% CI)** | **Standard Deviation of Costs**  **(95% CI)** |
| --- | --- | --- | --- |
| ***Societal*** |  |  |  |
| *Total Costs* | 14 | $27,380.74  ($4,075.53, $50,685.96) | $33,249.11  ($7,228.24, $59,269.99) |
| *Home Health Care* | 21 | $3,370.50  ($2,266.76, $4,474.23) | $4,453.91  ($2,941.22, $5,966.60) |
| *Assisted Services* | 16 | $3,463.14  ($335.50, $6,590.79) | $4,566.43  ($956.16, $8,176.69) |
| ***Health Care System*** |  |  |  |
| *Total Costs* | 45 | $24,195.52  ($9,679.77, $38,711.27) | $21,606.31  ($13,824.75, $29,387.88) |
| *Hospital* | 45 | $16,783.25  ($5,332.21, $28,234.29) | $18,387.83  ($6,868.35, $29,907.31) |
| *Clinical* | 28 | $7,357.55  ($-2,568.05, $17,283.14) | $7,400.77  (-$2,442.89, $17,244.43) |
| *Outpatient* | 33 | $9,401.63  ($835.10, $17,968.15) | $10,528.58  ($1,589.07, $19,468.09) |
| ***Personal*** |  |  |  |
| *Total Costs* | 9 | $7,455.49  ($2,271.45, $12,639.53) | $11,208.48  ($5,262.09, $17,154.87) |
| *Home-Help (Indirect Costs)* | 28 | $14,075.00  ($7,294.50, $20,855.51) | $15,851.18  ($9,090.34, $22,612.02) |
| *Medications* | 22 | $1,170.08  ($726.21, $1,613.95) | $1,325.71  ($758.65, $1,892.77) |
| *Direct Health Care Related Costs* | 8 | $3,929.46  ($781.19, $7077.73) | $4,602.58  ($1,692.77, $7,512.39) |

**Appendix Table 4.** Mean and standard deviations of annual costs per person of functional independence limitations based on markers of frailty, mobility, or cognition and each cost perspective

| Index | n_studies_ | Mean Cost (95% CI) | Standard Deviation of Costs (95% CI) |
| --- | --- | --- | --- |
| **Total Costs to Society** |  |  |  |
| *Frailty* | 11 | $30,045.48  ($482.68, $59,608.28) | $37,514.32  ($4,716.35, $70,312.29) |
| *Mobility* | 1 | $3,135.51 | $3,904.01 |
| *Cognitive* | 10 | $32,623.61  ($346.64, $64,900.58) | $41,027.85  ($5,579.43, $76,476.26) |
| **Total Costs to the Healthcare System** |  |  |  |
| Frailty | 25 | $21,266.01  ($11,763.67, $30,768.35) | $25,099.65  ($14,910.50, $35,288.79) |
| Mobility | 3 | $12,093.88  (-$7,843.04, $32,030.80) | $13,302.23  (-$5,641.10, $32,245.56) |
| Cognitive | 20 | $31,597.36  ($560.37, $62,634.35) | $22,367.03  ($9,122.42, $35,611.65) |
| **Total Costs to the Person** |  |  |  |
| Frailty | 7 | $8,294.09  ($1,670.40, $14,917.79) | $11,811.84  ($4,466.86, $19,156.82) |
| Mobility | 0 | -- | -- |
| Cognitive | 4 | $12,628.77  ($2,816.31, $22,441.24) | $15,308.67  ($3,578.20, $27,039.12) |

**Appendix Table 5.** Modified version of CHEERS checklist to assess quality of economic and cost-of-illness studies

| ***Items**** | **1** | **2** | **3** | **4** | **5** | **6** | **7** | **8** | **9** | **10** | **11** | **12** | **13** | **14** | **15** | **16** | **17** | **18** | **19** | **20** | **21** | **22** | **23** |
| --- | --- | --- | --- | --- | --- | --- | --- | --- | --- | --- | --- | --- | --- | --- | --- | --- | --- | --- | --- | --- | --- | --- | --- |
| ***Publication*** |  |  |  |  |  |  |  |  |  |  |  |  |  |  |  |  |  |  |  |  |  |  |  |
| Åkerborg 2016 | + | + | + | + | - | N/A | + | N/A | + | N/A | N/A | + | + | N/A | N/A | + | + | N/A | N/A | + | + | + | + |
| Akincigil 2020 | + | + | + | + | - | N/A | + | N/A | N/A | N/A | N/A | - | + | N/A | N/A | + | + | N/A | N/A | + | + | + | + |
| Ankuda 2017 | + | + | + | + | - | N/A | + | N/A | N/A | N/A | N/A | + | + | N/A | N/A | + | + | N/A | N/A | + | + | + | + |
| Beech 1999 | + | + | + | + | - | + | + | N/A | + | + | N/A | + | + | N/A | N/A | + | + | + | + | + | + | + | - |
| Bleijenberg 2017 | + | + | + | + | + | + | + | N/A | + | + | N/A | + | + | N/A | N/A | + | + | + | + | + | + | + | + |
| Boult 2001 | - | + | + | + | - | + | + | - | + | + | N/A | - | - | N/A | N/A | + | + | + | - | + | + | + | - |
| Braithwaite 2003 | + | + | + | + | + | N/A | + | + | N/A | N/A | N/A | - | + | N/A | N/A | - | + | N/A | + | + | + | + | - |
| Brinda 2014 | + | + | + | + | + | N/A | + | N/A | N/A | N/A | N/A | + | + | N/A | N/A | + | + | N/A | N/A | + | + | + | + |
| Challis 2002 | - | + | + | + | - | + | + | N/A | + | + | N/A | + | - | N/A | N/A | - | + | + | - | + | + | + | - |
| Challis 2004 | - | + | + | + | - | + | + | N/A | + | + | N/A | + | + | N/A | N/A | + | + | + | - | + | + | + | - |
| Chavan 2020 | + | - | - | + | - | - | - | - | - | N/A | N/A | - | - | N/A | N/A | + | + | N/A | - | - | + | + | + |
| Chen 2000/2001 | + | - | + | + | - | N/A | + | N/A | + | N/A | N/A | + | + | N/A | N/A | + | + | N/A | + | + | + | - | - |
| Chiatti 2015 | + | + | + | + | + | N/A | + | N/A | N/A | N/A | N/A | + | - | N/A | N/A | + | + | N/A | N/A | + | + | + | + |
| Chiu 2000 | + | + | + | + | - | N/A | + | N/A | + | N/A | N/A | + | + | N/A | N/A | + | + | N/A | N/A | + | + | + | - |
| Coleman 1999 | - | + | + | + | - | + | + | N/A | + | + | N/A | + | - | N/A | N/A | + | + | + | - | + | + | + | - |
| Colon-Emeric 2020 | + | + | + | + | - | + | + | - | + | N/A | N/A | + | - | N/A | N/A | + | + | N/A | - | - | + | + | + |
| Davis 2011 | + | + | + | + | + | + | + | - | + | + | N/A | + | + | N/A | N/A | + | + | + | + | + | + | + | + |
| Deardorff 2019 | + | + | + | + | - | N/A | + | N/A | N/A | N/A | N/A | + | + | N/A | N/A | + | + | N/A | N/A | + | + | + | + |
| Deb 2018 | + | + | + | + | - | N/A | + | N/A | N/A | N/A | N/A | + | + | N/A | N/A | + | + | N/A | N/A | + | + | + | + |
| Del Sindaco 2007 | - | + | + | + | - | + | + | - | + | + | N/A | - | - | N/A | N/A | + | + | + | - | + | + | + | - |

*Appendix Table 5 Continued*

| ***Items**** | **1** | **2** | **3** | **4** | **5** | **6** | **7** | **8** | **9** | **10** | **11** | **12** | **13** | **14** | **15** | **16** | **17** | **18** | **19** | **20** | **21** | **22** | **23** |
| --- | --- | --- | --- | --- | --- | --- | --- | --- | --- | --- | --- | --- | --- | --- | --- | --- | --- | --- | --- | --- | --- | --- | --- |
| ***Publication*** |  |  |  |  |  |  |  |  |  |  |  |  |  |  |  |  |  |  |  |  |  |  |  |
| Dodel 2015 | + | + | + | + | + | N/A | + | N/A | N/A | N/A | N/A | + | + | N/A | N/A | + | + | N/A | + | + | + | + | + |
| Eamer 2018 | + | + | + | + | - | N/A | + | N/A | N/A | N/A | N/A | + | + | N/A | N/A | + | + | N/A | + | + | + | + | + |
| Eamer 2019 | + | + | + | + | + | N/A | + | N/A | + | N/A | N/A | + | + | N/A | N/A | + | + | N/A | N/A | + | + | + | + |
| Ensrud 2018 | + | + | + | + | - | N/A | + | N/A | N/A | N/A | N/A | + | + | N/A | N/A | + | + | N/A | N/A | + | + | + | + |
| Fairhall 2015 | + | + | + | + | + | + | + | N/A | + | + | N/A | + | + | N/A | N/A | + | + | + | - | + | + | + | + |
| Farre 2016 | + | + | + | + | + | N/A | + | N/A | N/A | N/A | N/A | + | + | N/A | N/A | + | + | N/A | + | + | + | + | + |
| Forster 2009 | + | + | + | + | - | + | + | N/A | + | + | N/A | + | - | N/A | N/A | + | + | + | - | + | + | + | + |
| Graff 2008 | + | + | + | + | + | + | + | N/A | + | + | N/A | + | - | N/A | N/A | + | + | + | + | + | + | + | + |
| Gustavsson 2010 | + | + | + | + | - | N/A | + | N/A | N/A | N/A | N/A | + | + | N/A | N/A | + | + | N/A | N/A | + | + | - | - |
| Han 2019 | + | + | + | + | + | N/A | + | N/A | N/A | N/A | N/A | + | + | N/A | N/A | + | + | N/A | N/A | + | + | + | + |
| Hardy 2010 | + | + | + | + | - | N/A | + | N/A | N/A | N/A | N/A | + | + | N/A | N/A | + | + | N/A | N/A | + | + | + | + |
| Harrow 2004 | + | - | + | + | - | N/A | + | N/A | N/A | N/A | N/A | + | + | N/A | N/A | + | + | N/A | N/A | + | + | + | - |
| Hay 2002 | + | + | + | + | + | + | + | N/A | N/A | + | N/A | + | + | N/A | N/A | + | + | + | - | + | + | + | - |
| Hektoen 2016 | + | + | + | + | + | N/A | + | N/A | N/A | N/A | N/A | + | + | N/A | N/A | + | + | N/A | N/A | + | + | + | + |
| Hendriks 2008 | + | + | + | + | + | + | + | N/A | + | + | N/A | + | + | N/A | N/A | + | + | + | + | + | + | + | - |
| Hughes 2000 | - | + | + | + | - | + | + | + | + | + | N/A | + | + | N/A | N/A | + | + | + | + | + | + | + | - |
| Hui 1995 | - | + | + | + | - | + | + | N/A | + | + | N/A | + | - | N/A | N/A | + | + | + | - | + | + | - | - |
| Joling 2015 | + | + | + | + | + | N/A | + | + | N/A | N/A | N/A | + | + | N/A | N/A | + | + | N/A | + | + | + | + | + |
| Kehusmaa 2013 | + | + | + | + | - | N/A | + | + | + | N/A | N/A | + | + | N/A | N/A | + | + | N/A | N/A | + | + | - | + |
| Komisar 1997 | + | - | + | + | + | N/A | + | N/A | N/A | N/A | N/A | + | - | N/A | N/A | + | + | N/A | N/A | + | + | + | - |
| Kramer 1997 | + | + | + | + | + | N/A | + | N/A | + | N/A | N/A | + | - | N/A | N/A | + | + | N/A | N/A | + | + | + | - |
| Kronborg 2006 | + | + | + | + | + | + | + | + | + | + | N/A | + | + | N/A | N/A | + | + | + | + | + | + | + | + |
| Ku 2019 | + | + | + | + | - | N/A | + | N/A | N/A | N/A | N/A | + | + | N/A | N/A | + | + | N/A | N/A | + | + | + | + |
| Lafortune 2020 | - | + | + | + | - | N/A | + | N/A | N/A | N/A | N/A | + | + | N/A | N/A | + | + | N/A | + | + | + | + | - |

*Appendix Table 5 Continued*

| ***Items**** | **1** | **2** | **3** | **4** | **5** | **6** | **7** | **8** | **9** | **10** | **11** | **12** | **13** | **14** | **15** | **16** | **17** | **18** | **19** | **20** | **21** | **22** | **23** |
| --- | --- | --- | --- | --- | --- | --- | --- | --- | --- | --- | --- | --- | --- | --- | --- | --- | --- | --- | --- | --- | --- | --- | --- |
| ***Publication*** |  |  |  |  |  |  |  |  |  |  |  |  |  |  |  |  |  |  |  |  |  |  |  |
| Langa 2004 | + | + | + | + | - | N/A | + | N/A | N/A | N/A | N/A | + | - | N/A | N/A | + | + | N/A | + | + | + | + | - |
| Leeuwan 2015 | + | + | + | + | + | + | + | + | + | + | N/A | + | + | N/A | N/A | + | + | + | + | + | + | + | + |
| Lewin 2013 | + | - | + | + | - | + | + | N/A | N/A | + | N/A | + | - | N/A | N/A | + | + | + | - | + | + | + | - |
| Liotta 2019 | + | + | + | + | - | N/A | + | N/A | + | N/A | N/A | + | - | N/A | N/A | + | + | N/A | N/A | + | + | + | + |
| Lu 2020 | + | + | + | + | - | N/A | + | + | + | N/A | N/A | + | + | N/A | N/A | + | + | N/A | - | - | + | + | + |
| Mann 1999 | + | + | + | + | - | + | + | N/A | + | + | N/A | + | - | N/A | N/A | + | + | + | - | + | + | + | - |
| Marshall 1999 | - | - | + | + | - | + | + | N/A | + | + | N/A | + | - | N/A | N/A | + | + | + | - | + | + | + | - |
| Maru 2015 | + | + | + | + | + | + | + | + | + | + | N/A | + | + | N/A | N/A | + | + | + | + | + | + | + | + |
| Max 1995 | - | - | + | + | - | N/A | + | N/A | N/A | N/A | N/A | + | + | N/A | N/A | + | + | N/A | N/A | + | + | + | - |
| McCusker 2003 | + | + | + | + | + | + | + | N/A | + | + | N/A | + | + | N/A | N/A | + | + | + | - | + | + | + | - |
| McNamee 1999 | + | + | + | + | - | + | + | N/A | N/A | N/A | N/A | + | + | N/A | N/A | + | + | N/A | N/A | + | + | - | - |
| Melin 1993 | + | - | + | + | - | + | + | N/A | + | - | N/A | - | + | N/A | N/A | + | + | + | - | + | + | + | - |
| Metzelthin 2015 | + | + | + | + | + | + | + | - | + | + | N/A | + | + | N/A | N/A | + | + | + | + | + | + | - | + |
| Michalowsky 2016 | + | + | + | + | + | N/A | + | N/A | N/A | N/A | N/A | + | + | N/A | N/A | + | + | N/A | + | + | + | - | + |
| Miller 2005 | + | + | + | + | + | + | + | N/A | + | + | N/A | + | + | N/A | N/A | + | + | + | + | + | + | + | + |
| Mintzer 1997 | - | + | + | + | - | N/A | + | N/A | + | N/A | N/A | - | - | N/A | N/A | + | - | N/A | N/A | + | + | - | - |
| Mitchell 2019 | + | + | + | + | + | N/A | + | N/A | N/A | N/A | N/A | + | + | N/A | N/A | + | + | N/A | N/A | + | + | + | + |
| Murray 2003 | + | - | + | + | - | N/A | + | N/A | N/A | N/A | N/A | + | + | N/A | N/A | + | + | N/A | N/A | + | + | + | - |
| Nikolaus 1999 | - | + | + | + | - | + | + | N/A | + | + | N/A | + | - | N/A | N/A | + | + | + | - | + | + | + | - |
| Pinedo‑Villanueva 2019 | + | + | + | + | + | N/A | + | N/A | N/A | N/A | N/A | + | + | N/A | N/A | + | + | N/A | + | + | + | + | + |
| Pitkala 2013 | - | + | + | + | - | + | + | - | + | + | N/A | + | + | N/A | N/A | + | + | + | - | + | + | + | + |
| Reuben 2004 | + | - | + | + | - | N/A | + | N/A | + | N/A | N/A | + | - | N/A | N/A | + | + | N/A | N/A | + | + | + | - |
| Rigaud 2002 | + | - | + | + | - | N/A | + | N/A | N/A | N/A | N/A | + | + | N/A | N/A | + | + | N/A | N/A | + | + | + | - |

*Appendix Table 5 Continued*

| ***Items**** | | | **1** | **2** | **3** | | **4** | | **5** | **6** | | **7** | | **8** | | **9** | | **10** | | **11** | **12** | | **13** | | **14** | | **15** | | **16** | | **17** | | **18** | | **19** | | **20** | **21** | **22** | **23** |  |
| --- | --- | --- | --- | --- | --- | --- | --- | --- | --- | --- | --- | --- | --- | --- | --- | --- | --- | --- | --- | --- | --- | --- | --- | --- | --- | --- | --- | --- | --- | --- | --- | --- | --- | --- | --- | --- | --- | --- | --- | --- | --- |
| ***Publication*** | | |  |  |  | |  | |  |  | |  | |  | |  | |  | |  |  | |  | |  | |  | |  | |  | |  | |  | |  |  |  |  |  |
| Rojas 2010 | | | + | + | + | | + | | - | N/A | | + | | N/A | | N/A | | N/A | | N/A | + | | + | | N/A | | N/A | | + | | + | | N/A | | N/A | | + | + | + | + |  |
| Ruchlin 2001 | | | + | + | + | | + | | + | + | | + | | + | | N/A | | + | | N/A | + | | + | | N/A | | N/A | | + | | + | | + | | + | | + | + | + | - |  |
| Ruikes 2018 | | | + | + | + | | + | | + | + | | + | | N/A | | + | | + | | N/A | + | | + | | N/A | | N/A | | + | | + | | + | | + | | + | + | + | + |  |
| Schnaider-Beeri 2002a | + | + | + | + | | - | | N/A | | | + | | N/A | | N/A | | N/A | | N/A | | | + | | + | | N/A | | N/A | | - | | + | | N/A | | N/A | | - | + | + | - |
| Schnaider-Beeri 2002b | + | + | + | + | | - | | N/A | | | + | | N/A | | N/A | | N/A | | N/A | | | + | | + | | N/A | | N/A | | - | | + | | N/A | | N/A | | - | + | + | - |
| Schousboe 2019 | | | + | + | + | | + | | - | N/A | | + | | N/A | | N/A | | N/A | | N/A | + | | + | | N/A | | N/A | | + | | + | | N/A | | N/A | | + | + | + | + |  |
| Schraeder 2008 | | | - | + | + | | + | | - | + | | + | | N/A | | + | | N/A | | N/A | + | | - | | N/A | | N/A | | + | | + | | + | | - | | + | + | + | + |  |
| Schwarzkopf 2011 | | | + | + | + | | + | | + | + | | + | | + | | N/A | | N/A | | N/A | + | | + | | N/A | | N/A | | + | | + | | + | | + | | + | + | + | + |  |
| Scott 2004 | | | - | + | + | | + | | - | + | | + | | - | | + | | + | | N/A | + | | - | | N/A | | N/A | | + | | + | | + | | - | | + | + | + | - |  |
| Taylor 2001 | | | + | - | + | | + | | + | N/A | | + | | N/A | | N/A | | N/A | | N/A | + | | + | | N/A | | N/A | | + | | + | | N/A | | N/A | | + | + | + | - |  |
| Van Lier 2016 | | | + | + | + | | + | | + | N/A | | + | | N/A | | + | | N/A | | N/A | + | | + | | N/A | | N/A | | + | | + | | N/A | | + | | + | + | + | + |  |
| Wang 2008 | | | + | + | + | | + | | - | N/A | | + | | N/A | | N/A | | N/A | | N/A | + | | + | | N/A | | N/A | | + | | + | | N/A | | N/A | | + | + | + | - |  |
| Wang 2010 | | | + | - | + | | + | | - | N/A | | + | | N/A | | N/A | | N/A | | N/A | + | | + | | N/A | | N/A | | + | | + | | N/A | | N/A | | + | + | + | - |  |
| Witham 2012 | | | + | + | + | | + | | - | + | | + | | N/A | | + | | + | | N/A | + | | - | | N/A | | N/A | | + | | + | | + | | + | | + | + | + | - |  |
| Wolff 2019 | | | + | + | + | | + | | - | N/A | | + | | N/A | | N/A | | N/A | | N/A | - | | - | | N/A | | N/A | | + | | + | | N/A | | + | | + | + | + | + |  |
| Wolstenholme 2002 | | | + | + | + | | + | | - | N/A | | + | | N/A | | N/A | | N/A | | N/A | + | | + | | N/A | | N/A | | + | | + | | N/A | | N/A | | + | + | + | + |  |
| Woods 2012 | | | + | + | + | | + | | + | + | | + | | N/A | | + | | + | | N/A | + | | + | | N/A | | N/A | | + | | + | | + | | - | | + | + | + | + |  |
| Wübker 2014 | | | + | + | + | | + | | + | N/A | | + | | N/A | | N/A | | N/A | | N/A | + | | + | | N/A | | N/A | | + | | + | | N/A | | + | | + | + | + | - |  |
| Zhang 2003 | | | + | + | + | | + | | - | N/A | | - | | N/A | | N/A | | N/A | | N/A | + | | + | | N/A | | N/A | | - | | + | | N/A | | N/A | | + | + | + | - |  |

**1* Title *2* Abstract *3* Introduction: Background and Objectives *4* Methods: Target Population and Subgroups, Setting, and Location *5* Study Perspective *6* Comparators *7* Time Horizon *8* Discount Rate *9* Choice of Health Outcomes *10* Measurement of Effectiveness *11* Measurement and Valuation of Preference Based Outcomes *12* Estimating Resources and Costs *13* Currency, Price Data and Conversion *14* Choice of Model *15* Assumptions *16* Analytical Methods *17* Study Parameters *18* Incremental Costs and Outcomes *19* Characterizing Uncertainty *20* Characterizing Heterogeneity *21* Discussion: Study Findings, Limitations, Generalizability and Current Knowledge *22* Source of Funding *23* Conflicts of Interest
