# Supplementary material for: International depiction of the cost of functional independence limitations among older adults living in the community: a systematic review and cost-of-impairment study
Source: BMC Geriatr. 2022 Oct 22;22:815. doi: 10.1186/s12877-022-03466-w (PMC9587635; doi:10.1186/s12877-022-03466-w)
Supplement: Supplementary file 2 — Additional file 2 Supplementary Material S1. R code and output. [file 12877_2022_3466_MOESM2_ESM.docx]

Supplementary Material S1: R code and output

Ryan Stanley Falck

17/02/2021

#DATA MANAGEMENT

if (!require("pacman")) install.packages("pacman")

## Loading required package: pacman

pacman::p_load(MASS,plyr,psych, dplyr, tableone,psych,Hmisc,openxlsx,rcompanion, Rmisc, lsmeans)

setwd("C:/Users/falckr/Desktop/Manuscripts/Ongoing Projects/SR on Economic Impact of Frailty")
data1<-read.xlsx("Cost data for meta v2.xlsx")


data1<-rename(data1, c("Study_Length"="Length.(Years)"))

data1$Annual.Costs.mean <- data1$Costs.mean/data1$Study_Length
data1$Annual.Costs.sd <- data1$Costs.SD/data1$Study_Length

data1$Cost.Perspective<-NA
data1$Cost.Perspective[data1$Cost.persective == "Social" | data1$Cost.persective == "Societal" | data1$Cost.persective == "Societal "] <- "Societal"
data1$Cost.Perspective[data1$Cost.persective == "Healthcare"] <- "Healthcare"
data1$Cost.Perspective[data1$Cost.persective == "Personal" | data1$Cost.persective == "Patient"] <- "Personal"


table(data1$Cost.Perspective)

##
## Healthcare Personal Societal
## 150 64 50

data2<- subset(data1, !is.na(Cost.Perspective))

table(data1$Study, data1$Cost.Perspective)

##
## Healthcare Personal Societal
## Akerborg, 2016 2 1 2
## Akincigil, 2020 3 0 1
## Ankuda, 2017 1 0 0
## Beech, 1999 3 0 0
## Beeri, 2002 1 4 0
## Bleijenberg, 2017 2 1 1
## Boult, 2001 4 0 1
## Braithwaite, 2003 1 0 0
## Brinda, 2014 0 1 0
## Challis, 2002 4 1 3
## Challis, 2004 1 1 1
## Chavan 2020 1 0 0
## Chen, 2001 1 0 0
## Chiatti, 2015 3 3 1
## Chiu, 2000 0 2 0
## Coleman, 1999 0 1 0
## Colon-Emeric 2020 1 0 0
## Davis, 2011 1 0 0
## Deardorff, 2019 1 0 0
## Deb, 2018 3 1 1
## Del Sindaco, 2007 1 0 0
## Dodel, 2015 0 0 1
## Eamer, 2018 1 1 0
## Eamer, 2019 1 1 0
## Ensrud, 2018 2 0 0
## Fairhall, 2015 3 0 3
## Farre, 2016 2 2 0
## Forster, 2009 2 1 0
## Graff, 2008 2 1 2
## Gustavsson, 2010 1 1 1
## Han,2019 1 0 0
## Hardy, 2010 1 1 0
## Harrow, 2004 1 1 0
## Hay, 2002 1 1 0
## Hektoen, 2016 3 1 2
## Hendriks, 2008 3 4 0
## Hughes, 2000 3 0 1
## Hui, 1995 2 0 0
## Joling, 2015 1 2 1
## Kehusmaa, 2013 0 0 0
## Komisar 1997 3 0 1
## Kramer 1997 3 0 0
## Kronborg, 2006 3 1 2
## Ku, 2019 1 1 1
## Lafortune, 2020 2 0 1
## Langa, 2004 0 1 0
## Leeuwan, 2015 3 2 2
## Lewin, 2013 1 0 0
## Liotta, 2019 2 0 0
## Lu 2020 3 0 1
## Mann, 1999 2 0 0
## Marshall, 1999 3 0 0
## Maru,2015 2 1 0
## Max, 1995 0 1 0
## McCusker, 2003 2 2 0
## McNamee, 1999 0 0 1
## Melin, 1993 3 0 0
## Metzelthin, 2015 3 3 1
## Michalowsky, 2016 0 1 1
## Miller, 2005 3 0 1
## Mintzer, 1997 1 0 0
## Mitchell, 2019 1 0 0
## Murray, 2003 1 0 0
## Nikolaus, 1999 2 0 1
## Pinedo-Villanueva, 2019 2 2 0
## Pitkala, 2013 2 1 3
## Reuben, 2004 1 0 0
## Rigaud, 2002 1 2 0
## Rojas, 2010 4 1 0
## Ruchlin, 2001 2 1 0
## Ruikes, 2018 3 2 2
## Schousboe, 2019 2 0 0
## Schraeder, 2008 1 0 0
## Schwardzkopf, 2011 3 2 1
## Scott, 2004 5 0 1
## Taylor, 2003 1 2 0
## Van Lier, 2016 2 1 3
## Wang, 2008 1 2 0
## Wang, 2010 0 1 0
## Witham, 2012 4 1 0
## Wolff, 2019 3 0 1
## Wolstenholme, 2002 0 0 0
## Woods, 2012 2 0 2
## Wubker, 2014 0 0 1
## Zhang, 2007 3 0 1

data3<- data2 %>%
 mutate(Annual.Costs.sd.liberal = ifelse(is.na(Annual.Costs.sd), Annual.Costs.mean, Annual.Costs.sd),
 Annual.Costs.sd.conservative = ifelse(is.na(Annual.Costs.sd), Annual.Costs.mean*2, Annual.Costs.sd))

data3$costs.subtype.2<-NA
data3$costs.subtype.2[data3$Cost.Subtype == "Clinical" | data3$Cost.Subtype == "Clinician"]<-"Clinician"
data3$costs.subtype.2[data3$Cost.Subtype == "Hospital costs" | data3$Cost.Subtype == "Hospital"]<-"Hospital"
data3$costs.subtype.2[data3$Cost.Subtype == "All" | data3$Cost.Subtype == "Total"]<-"All"
data3$costs.subtype.2[data3$Cost.Subtype == "Assisted Services" | data3$Cost.Subtype == "Day care"]<-"Assisted Services"
data3$costs.subtype.2[data3$Cost.Subtype == "Direct Health Care Related Costs"]<-"Direct Healthcare Related Costs"
data3$costs.subtype.2[data3$Cost.Subtype == "Home-Help Indirect"]<-"Home-Help Indirect"
data3$costs.subtype.2[data3$Cost.Subtype == "Home Healthcare"]<-"Home Healthcare"
data3$costs.subtype.2[data3$Cost.Subtype == "Medications"]<-"Medications"
data3$costs.subtype.2[data3$Cost.Subtype == "Outpatient"]<-"Outpatient"

data3$Cost.Perspective[data3$costs.subtype.2 == "Medications" & data3$Cost.Perspective == "Healthcare"]<-"Personal"
data3$Cost.Perspective[data3$costs.subtype.2 == "Outpatient" & data3$Cost.Perspective == "Societal"]<-"Healthcare"
data3$Cost.Perspective[data3$costs.subtype.2 == "Home Healthcare" & data3$Cost.Perspective == "Personal"]<-"Societal"


table(data3$Study, data3$costs.subtype.2)

##
## All Assisted Services Clinician
## Akerborg, 2016 0 1 0
## Akincigil, 2020 1 0 0
## Ankuda, 2017 1 0 0
## Beech, 1999 1 0 0
## Beeri, 2002 1 0 1
## Bleijenberg, 2017 1 1 0
## Boult, 2001 1 0 1
## Braithwaite, 2003 1 0 0
## Brinda, 2014 0 0 0
## Challis, 2002 3 1 1
## Challis, 2004 3 0 0
## Chavan 2020 0 0 0
## Chen, 2001 1 0 0
## Chiatti, 2015 3 0 0
## Chiu, 2000 0 0 0
## Coleman, 1999 0 0 0
## Colon-Emeric 2020 0 0 0
## Davis, 2011 1 0 0
## Deardorff, 2019 1 0 0
## Deb, 2018 1 0 0
## Del Sindaco, 2007 0 0 0
## Dodel, 2015 1 0 0
## Eamer, 2018 0 0 1
## Eamer, 2019 1 0 0
## Ensrud, 2018 1 0 0
## Fairhall, 2015 1 1 1
## Farre, 2016 0 0 0
## Forster, 2009 0 0 0
## Graff, 2008 0 1 1
## Gustavsson, 2010 2 0 0
## Han,2019 1 0 0
## Hardy, 2010 2 0 0
## Harrow, 2004 1 0 0
## Hay, 2002 1 0 0
## Hektoen, 2016 0 1 1
## Hendriks, 2008 2 0 1
## Hughes, 2000 1 0 0
## Hui, 1995 0 0 0
## Joling, 2015 2 0 0
## Komisar 1997 0 0 1
## Kramer 1997 1 0 0
## Kronborg, 2006 0 1 1
## Ku, 2019 3 0 0
## Lafortune, 2020 1 0 1
## Langa, 2004 0 0 0
## Leeuwan, 2015 0 1 1
## Lewin, 2013 0 0 0
## Liotta, 2019 1 0 0
## Lu 2020 1 1 1
## Mann, 1999 1 0 0
## Marshall, 1999 1 0 0
## Maru,2015 1 0 0
## Max, 1995 0 0 0
## McCusker, 2003 1 0 0
## McNamee, 1999 1 0 0
## Melin, 1993 1 0 0
## Metzelthin, 2015 2 0 1
## Michalowsky, 2016 0 0 0
## Miller, 2005 0 1 1
## Mintzer, 1997 1 0 0
## Mitchell, 2019 1 0 0
## Murray, 2003 0 0 0
## Nikolaus, 1999 0 1 1
## Pinedo-Villanueva, 2019 0 0 1
## Pitkala, 2013 0 1 1
## Reuben, 2004 0 0 0
## Rigaud, 2002 1 0 0
## Rojas, 2010 1 0 1
## Ruchlin, 2001 1 0 1
## Ruikes, 2018 0 1 1
## Schousboe, 2019 1 0 0
## Schraeder, 2008 1 0 0
## Schwardzkopf, 2011 1 0 1
## Scott, 2004 1 0 1
## Taylor, 2003 1 0 0
## Van Lier, 2016 1 1 1
## Wang, 2008 1 0 0
## Wang, 2010 0 0 0
## Witham, 2012 1 0 1
## Wolff, 2019 1 0 1
## Woods, 2012 1 1 1
## Wubker, 2014 1 0 0
## Zhang, 2007 0 0 1
##
## Direct Healthcare Related Costs Home-Help Indirect
## Akerborg, 2016 0 1
## Akincigil, 2020 0 0
## Ankuda, 2017 0 0
## Beech, 1999 0 0
## Beeri, 2002 1 1
## Bleijenberg, 2017 0 1
## Boult, 2001 0 0
## Braithwaite, 2003 0 0
## Brinda, 2014 0 1
## Challis, 2002 0 0
## Challis, 2004 0 0
## Chavan 2020 0 0
## Chen, 2001 0 0
## Chiatti, 2015 0 1
## Chiu, 2000 1 1
## Coleman, 1999 0 0
## Colon-Emeric 2020 0 0
## Davis, 2011 0 0
## Deardorff, 2019 0 0
## Deb, 2018 0 0
## Del Sindaco, 2007 0 0
## Dodel, 2015 0 0
## Eamer, 2018 1 0
## Eamer, 2019 0 0
## Ensrud, 2018 0 0
## Fairhall, 2015 0 0
## Farre, 2016 0 1
## Forster, 2009 0 0
## Graff, 2008 1 0
## Gustavsson, 2010 0 1
## Han,2019 0 0
## Hardy, 2010 0 0
## Harrow, 2004 0 1
## Hay, 2002 0 1
## Hektoen, 2016 1 0
## Hendriks, 2008 1 1
## Hughes, 2000 0 0
## Hui, 1995 0 0
## Joling, 2015 0 1
## Komisar 1997 0 0
## Kramer 1997 0 0
## Kronborg, 2006 0 0
## Ku, 2019 0 0
## Lafortune, 2020 0 0
## Langa, 2004 0 1
## Leeuwan, 2015 0 1
## Lewin, 2013 0 0
## Liotta, 2019 0 0
## Lu 2020 0 0
## Mann, 1999 0 0
## Marshall, 1999 0 0
## Maru,2015 0 0
## Max, 1995 0 1
## McCusker, 2003 1 0
## McNamee, 1999 0 0
## Melin, 1993 0 0
## Metzelthin, 2015 0 1
## Michalowsky, 2016 0 1
## Miller, 2005 0 0
## Mintzer, 1997 0 0
## Mitchell, 2019 0 0
## Murray, 2003 0 0
## Nikolaus, 1999 0 0
## Pinedo-Villanueva, 2019 0 1
## Pitkala, 2013 0 1
## Reuben, 2004 0 0
## Rigaud, 2002 1 1
## Rojas, 2010 0 0
## Ruchlin, 2001 0 1
## Ruikes, 2018 0 1
## Schousboe, 2019 0 0
## Schraeder, 2008 0 0
## Schwardzkopf, 2011 0 1
## Scott, 2004 0 0
## Taylor, 2003 0 1
## Van Lier, 2016 0 1
## Wang, 2008 0 1
## Wang, 2010 0 1
## Witham, 2012 0 0
## Wolff, 2019 0 0
## Woods, 2012 0 0
## Wubker, 2014 0 0
## Zhang, 2007 0 0
##
## Home Healthcare Hospital Medications Outpatient
## Akerborg, 2016 1 1 0 1
## Akincigil, 2020 1 1 0 1
## Ankuda, 2017 0 0 0 0
## Beech, 1999 0 1 0 1
## Beeri, 2002 0 0 1 0
## Bleijenberg, 2017 0 1 0 0
## Boult, 2001 1 1 0 1
## Braithwaite, 2003 0 0 0 0
## Brinda, 2014 0 0 0 0
## Challis, 2002 1 1 0 1
## Challis, 2004 0 0 0 0
## Chavan 2020 0 1 0 0
## Chen, 2001 0 0 0 0
## Chiatti, 2015 0 1 1 1
## Chiu, 2000 0 0 0 0
## Coleman, 1999 0 0 1 0
## Colon-Emeric 2020 0 1 0 0
## Davis, 2011 0 0 0 0
## Deardorff, 2019 0 0 0 0
## Deb, 2018 1 1 1 1
## Del Sindaco, 2007 0 1 0 0
## Dodel, 2015 0 0 0 0
## Eamer, 2018 0 0 0 0
## Eamer, 2019 0 0 0 1
## Ensrud, 2018 0 0 0 1
## Fairhall, 2015 1 1 0 1
## Farre, 2016 0 1 1 1
## Forster, 2009 0 1 1 1
## Graff, 2008 1 1 0 0
## Gustavsson, 2010 0 0 0 0
## Han,2019 0 0 0 0
## Hardy, 2010 0 0 0 0
## Harrow, 2004 0 0 0 0
## Hay, 2002 0 0 0 0
## Hektoen, 2016 1 1 0 1
## Hendriks, 2008 0 1 1 0
## Hughes, 2000 1 1 0 1
## Hui, 1995 0 1 0 1
## Joling, 2015 0 0 1 0
## Komisar 1997 1 1 0 1
## Kramer 1997 0 1 0 1
## Kronborg, 2006 1 1 1 1
## Ku, 2019 0 0 0 0
## Lafortune, 2020 0 1 0 0
## Langa, 2004 0 0 0 0
## Leeuwan, 2015 1 1 1 1
## Lewin, 2013 0 1 0 0
## Liotta, 2019 0 1 0 0
## Lu 2020 0 0 0 1
## Mann, 1999 0 1 0 0
## Marshall, 1999 0 1 0 1
## Maru,2015 0 1 1 0
## Max, 1995 0 0 0 0
## McCusker, 2003 0 1 1 0
## McNamee, 1999 0 0 0 0
## Melin, 1993 0 1 0 1
## Metzelthin, 2015 1 1 1 0
## Michalowsky, 2016 1 0 0 0
## Miller, 2005 0 1 0 1
## Mintzer, 1997 0 0 0 0
## Mitchell, 2019 0 0 0 0
## Murray, 2003 0 1 0 0
## Nikolaus, 1999 0 1 0 0
## Pinedo-Villanueva, 2019 0 0 1 1
## Pitkala, 2013 1 1 0 1
## Reuben, 2004 0 1 0 0
## Rigaud, 2002 0 0 0 0
## Rojas, 2010 0 1 1 1
## Ruchlin, 2001 0 0 0 0
## Ruikes, 2018 1 1 1 1
## Schousboe, 2019 0 0 0 1
## Schraeder, 2008 0 0 0 0
## Schwardzkopf, 2011 0 1 1 1
## Scott, 2004 1 1 1 1
## Taylor, 2003 0 0 1 0
## Van Lier, 2016 1 1 0 0
## Wang, 2008 0 0 1 0
## Wang, 2010 0 0 0 0
## Witham, 2012 0 1 1 1
## Wolff, 2019 1 0 0 1
## Woods, 2012 0 1 0 0
## Wubker, 2014 0 0 0 0
## Zhang, 2007 1 1 0 1

table(data3$Study, data3$Cost.Perspective, data3$costs.subtype.2=="All")

## , , = FALSE
##
##
## Healthcare Personal Societal
## Akerborg, 2016 2 1 2
## Akincigil, 2020 2 0 1
## Ankuda, 2017 0 0 0
## Beech, 1999 2 0 0
## Beeri, 2002 1 3 0
## Bleijenberg, 2017 1 1 1
## Boult, 2001 3 0 1
## Braithwaite, 2003 0 0 0
## Brinda, 2014 0 1 0
## Challis, 2002 3 0 2
## Challis, 2004 0 0 0
## Chavan 2020 1 0 0
## Chen, 2001 0 0 0
## Chiatti, 2015 2 2 0
## Chiu, 2000 0 2 0
## Coleman, 1999 0 1 0
## Colon-Emeric 2020 1 0 0
## Davis, 2011 0 0 0
## Deardorff, 2019 0 0 0
## Deb, 2018 2 1 1
## Del Sindaco, 2007 1 0 0
## Dodel, 2015 0 0 0
## Eamer, 2018 1 1 0
## Eamer, 2019 1 0 0
## Ensrud, 2018 1 0 0
## Fairhall, 2015 3 0 2
## Farre, 2016 2 2 0
## Forster, 2009 2 1 0
## Graff, 2008 2 1 2
## Gustavsson, 2010 0 1 0
## Han,2019 0 0 0
## Hardy, 2010 0 0 0
## Harrow, 2004 0 1 0
## Hay, 2002 0 1 0
## Hektoen, 2016 3 1 2
## Hendriks, 2008 2 3 0
## Hughes, 2000 2 0 1
## Hui, 1995 2 0 0
## Joling, 2015 0 2 0
## Komisar 1997 3 0 1
## Kramer 1997 2 0 0
## Kronborg, 2006 3 1 2
## Ku, 2019 0 0 0
## Lafortune, 2020 2 0 0
## Langa, 2004 0 1 0
## Leeuwan, 2015 3 2 2
## Lewin, 2013 1 0 0
## Liotta, 2019 1 0 0
## Lu 2020 2 0 1
## Mann, 1999 1 0 0
## Marshall, 1999 2 0 0
## Maru,2015 1 1 0
## Max, 1995 0 1 0
## McCusker, 2003 1 2 0
## McNamee, 1999 0 0 0
## Melin, 1993 2 0 0
## Metzelthin, 2015 2 2 1
## Michalowsky, 2016 0 1 1
## Miller, 2005 3 0 1
## Mintzer, 1997 0 0 0
## Mitchell, 2019 0 0 0
## Murray, 2003 1 0 0
## Nikolaus, 1999 2 0 1
## Pinedo-Villanueva, 2019 2 2 0
## Pitkala, 2013 3 1 2
## Reuben, 2004 1 0 0
## Rigaud, 2002 0 2 0
## Rojas, 2010 3 1 0
## Ruchlin, 2001 1 1 0
## Ruikes, 2018 3 2 2
## Schousboe, 2019 1 0 0
## Schraeder, 2008 0 0 0
## Schwardzkopf, 2011 3 2 0
## Scott, 2004 3 1 1
## Taylor, 2003 0 2 0
## Van Lier, 2016 2 1 2
## Wang, 2008 0 2 0
## Wang, 2010 0 1 0
## Witham, 2012 3 1 0
## Wolff, 2019 2 0 1
## Woods, 2012 2 0 1
## Wubker, 2014 0 0 0
## Zhang, 2007 3 0 1
##
## , , = TRUE
##
##
## Healthcare Personal Societal
## Akerborg, 2016 0 0 0
## Akincigil, 2020 1 0 0
## Ankuda, 2017 1 0 0
## Beech, 1999 1 0 0
## Beeri, 2002 0 1 0
## Bleijenberg, 2017 1 0 0
## Boult, 2001 1 0 0
## Braithwaite, 2003 1 0 0
## Brinda, 2014 0 0 0
## Challis, 2002 1 1 1
## Challis, 2004 1 1 1
## Chavan 2020 0 0 0
## Chen, 2001 1 0 0
## Chiatti, 2015 1 1 1
## Chiu, 2000 0 0 0
## Coleman, 1999 0 0 0
## Colon-Emeric 2020 0 0 0
## Davis, 2011 1 0 0
## Deardorff, 2019 1 0 0
## Deb, 2018 1 0 0
## Del Sindaco, 2007 0 0 0
## Dodel, 2015 0 0 1
## Eamer, 2018 0 0 0
## Eamer, 2019 0 1 0
## Ensrud, 2018 1 0 0
## Fairhall, 2015 0 0 1
## Farre, 2016 0 0 0
## Forster, 2009 0 0 0
## Graff, 2008 0 0 0
## Gustavsson, 2010 1 0 1
## Han,2019 1 0 0
## Hardy, 2010 1 1 0
## Harrow, 2004 1 0 0
## Hay, 2002 1 0 0
## Hektoen, 2016 0 0 0
## Hendriks, 2008 1 1 0
## Hughes, 2000 1 0 0
## Hui, 1995 0 0 0
## Joling, 2015 1 0 1
## Komisar 1997 0 0 0
## Kramer 1997 1 0 0
## Kronborg, 2006 0 0 0
## Ku, 2019 1 1 1
## Lafortune, 2020 0 0 1
## Langa, 2004 0 0 0
## Leeuwan, 2015 0 0 0
## Lewin, 2013 0 0 0
## Liotta, 2019 1 0 0
## Lu 2020 1 0 0
## Mann, 1999 1 0 0
## Marshall, 1999 1 0 0
## Maru,2015 1 0 0
## Max, 1995 0 0 0
## McCusker, 2003 1 0 0
## McNamee, 1999 0 0 1
## Melin, 1993 1 0 0
## Metzelthin, 2015 1 1 0
## Michalowsky, 2016 0 0 0
## Miller, 2005 0 0 0
## Mintzer, 1997 1 0 0
## Mitchell, 2019 1 0 0
## Murray, 2003 0 0 0
## Nikolaus, 1999 0 0 0
## Pinedo-Villanueva, 2019 0 0 0
## Pitkala, 2013 0 0 0
## Reuben, 2004 0 0 0
## Rigaud, 2002 1 0 0
## Rojas, 2010 1 0 0
## Ruchlin, 2001 1 0 0
## Ruikes, 2018 0 0 0
## Schousboe, 2019 1 0 0
## Schraeder, 2008 1 0 0
## Schwardzkopf, 2011 0 0 1
## Scott, 2004 1 0 0
## Taylor, 2003 1 0 0
## Van Lier, 2016 0 0 1
## Wang, 2008 1 0 0
## Wang, 2010 0 0 0
## Witham, 2012 1 0 0
## Wolff, 2019 1 0 0
## Woods, 2012 0 0 1
## Wubker, 2014 0 0 1
## Zhang, 2007 0 0 0

table(data3$Study, data3$costs.subtype.2=="Assisted Services")

##
## FALSE TRUE
## Akerborg, 2016 4 1
## Akincigil, 2020 4 0
## Ankuda, 2017 1 0
## Beech, 1999 3 0
## Beeri, 2002 5 0
## Bleijenberg, 2017 3 1
## Boult, 2001 5 0
## Braithwaite, 2003 1 0
## Brinda, 2014 1 0
## Challis, 2002 7 1
## Challis, 2004 3 0
## Chavan 2020 1 0
## Chen, 2001 1 0
## Chiatti, 2015 7 0
## Chiu, 2000 2 0
## Coleman, 1999 1 0
## Colon-Emeric 2020 1 0
## Davis, 2011 1 0
## Deardorff, 2019 1 0
## Deb, 2018 5 0
## Del Sindaco, 2007 1 0
## Dodel, 2015 1 0
## Eamer, 2018 2 0
## Eamer, 2019 2 0
## Ensrud, 2018 2 0
## Fairhall, 2015 5 1
## Farre, 2016 4 0
## Forster, 2009 3 0
## Graff, 2008 4 1
## Gustavsson, 2010 3 0
## Han,2019 1 0
## Hardy, 2010 2 0
## Harrow, 2004 2 0
## Hay, 2002 2 0
## Hektoen, 2016 5 1
## Hendriks, 2008 7 0
## Hughes, 2000 4 0
## Hui, 1995 2 0
## Joling, 2015 4 0
## Komisar 1997 4 0
## Kramer 1997 3 0
## Kronborg, 2006 5 1
## Ku, 2019 3 0
## Lafortune, 2020 3 0
## Langa, 2004 1 0
## Leeuwan, 2015 6 1
## Lewin, 2013 1 0
## Liotta, 2019 2 0
## Lu 2020 3 1
## Mann, 1999 2 0
## Marshall, 1999 3 0
## Maru,2015 3 0
## Max, 1995 1 0
## McCusker, 2003 4 0
## McNamee, 1999 1 0
## Melin, 1993 3 0
## Metzelthin, 2015 7 0
## Michalowsky, 2016 2 0
## Miller, 2005 3 1
## Mintzer, 1997 1 0
## Mitchell, 2019 1 0
## Murray, 2003 1 0
## Nikolaus, 1999 2 1
## Pinedo-Villanueva, 2019 4 0
## Pitkala, 2013 5 1
## Reuben, 2004 1 0
## Rigaud, 2002 3 0
## Rojas, 2010 5 0
## Ruchlin, 2001 3 0
## Ruikes, 2018 6 1
## Schousboe, 2019 2 0
## Schraeder, 2008 1 0
## Schwardzkopf, 2011 6 0
## Scott, 2004 6 0
## Taylor, 2003 3 0
## Van Lier, 2016 5 1
## Wang, 2008 3 0
## Wang, 2010 1 0
## Witham, 2012 5 0
## Wolff, 2019 4 0
## Woods, 2012 3 1
## Wubker, 2014 1 0
## Zhang, 2007 4 0

table(data3$Study, data3$costs.subtype.2=="Home Healthcare")

##
## FALSE TRUE
## Akerborg, 2016 4 1
## Akincigil, 2020 3 1
## Ankuda, 2017 1 0
## Beech, 1999 3 0
## Beeri, 2002 5 0
## Bleijenberg, 2017 4 0
## Boult, 2001 4 1
## Braithwaite, 2003 1 0
## Brinda, 2014 1 0
## Challis, 2002 7 1
## Challis, 2004 3 0
## Chavan 2020 1 0
## Chen, 2001 1 0
## Chiatti, 2015 7 0
## Chiu, 2000 2 0
## Coleman, 1999 1 0
## Colon-Emeric 2020 1 0
## Davis, 2011 1 0
## Deardorff, 2019 1 0
## Deb, 2018 4 1
## Del Sindaco, 2007 1 0
## Dodel, 2015 1 0
## Eamer, 2018 2 0
## Eamer, 2019 2 0
## Ensrud, 2018 2 0
## Fairhall, 2015 5 1
## Farre, 2016 4 0
## Forster, 2009 3 0
## Graff, 2008 4 1
## Gustavsson, 2010 3 0
## Han,2019 1 0
## Hardy, 2010 2 0
## Harrow, 2004 2 0
## Hay, 2002 2 0
## Hektoen, 2016 5 1
## Hendriks, 2008 7 0
## Hughes, 2000 3 1
## Hui, 1995 2 0
## Joling, 2015 4 0
## Komisar 1997 3 1
## Kramer 1997 3 0
## Kronborg, 2006 5 1
## Ku, 2019 3 0
## Lafortune, 2020 3 0
## Langa, 2004 1 0
## Leeuwan, 2015 6 1
## Lewin, 2013 1 0
## Liotta, 2019 2 0
## Lu 2020 4 0
## Mann, 1999 2 0
## Marshall, 1999 3 0
## Maru,2015 3 0
## Max, 1995 1 0
## McCusker, 2003 4 0
## McNamee, 1999 1 0
## Melin, 1993 3 0
## Metzelthin, 2015 6 1
## Michalowsky, 2016 1 1
## Miller, 2005 4 0
## Mintzer, 1997 1 0
## Mitchell, 2019 1 0
## Murray, 2003 1 0
## Nikolaus, 1999 3 0
## Pinedo-Villanueva, 2019 4 0
## Pitkala, 2013 5 1
## Reuben, 2004 1 0
## Rigaud, 2002 3 0
## Rojas, 2010 5 0
## Ruchlin, 2001 3 0
## Ruikes, 2018 6 1
## Schousboe, 2019 2 0
## Schraeder, 2008 1 0
## Schwardzkopf, 2011 6 0
## Scott, 2004 5 1
## Taylor, 2003 3 0
## Van Lier, 2016 5 1
## Wang, 2008 3 0
## Wang, 2010 1 0
## Witham, 2012 5 0
## Wolff, 2019 3 1
## Woods, 2012 4 0
## Wubker, 2014 1 0
## Zhang, 2007 3 1

table(data3$Study, data3$costs.subtype.2=="Clinician")

##
## FALSE TRUE
## Akerborg, 2016 5 0
## Akincigil, 2020 4 0
## Ankuda, 2017 1 0
## Beech, 1999 3 0
## Beeri, 2002 4 1
## Bleijenberg, 2017 4 0
## Boult, 2001 4 1
## Braithwaite, 2003 1 0
## Brinda, 2014 1 0
## Challis, 2002 7 1
## Challis, 2004 3 0
## Chavan 2020 1 0
## Chen, 2001 1 0
## Chiatti, 2015 7 0
## Chiu, 2000 2 0
## Coleman, 1999 1 0
## Colon-Emeric 2020 1 0
## Davis, 2011 1 0
## Deardorff, 2019 1 0
## Deb, 2018 5 0
## Del Sindaco, 2007 1 0
## Dodel, 2015 1 0
## Eamer, 2018 1 1
## Eamer, 2019 2 0
## Ensrud, 2018 2 0
## Fairhall, 2015 5 1
## Farre, 2016 4 0
## Forster, 2009 3 0
## Graff, 2008 4 1
## Gustavsson, 2010 3 0
## Han,2019 1 0
## Hardy, 2010 2 0
## Harrow, 2004 2 0
## Hay, 2002 2 0
## Hektoen, 2016 5 1
## Hendriks, 2008 6 1
## Hughes, 2000 4 0
## Hui, 1995 2 0
## Joling, 2015 4 0
## Komisar 1997 3 1
## Kramer 1997 3 0
## Kronborg, 2006 5 1
## Ku, 2019 3 0
## Lafortune, 2020 2 1
## Langa, 2004 1 0
## Leeuwan, 2015 6 1
## Lewin, 2013 1 0
## Liotta, 2019 2 0
## Lu 2020 3 1
## Mann, 1999 2 0
## Marshall, 1999 3 0
## Maru,2015 3 0
## Max, 1995 1 0
## McCusker, 2003 4 0
## McNamee, 1999 1 0
## Melin, 1993 3 0
## Metzelthin, 2015 6 1
## Michalowsky, 2016 2 0
## Miller, 2005 3 1
## Mintzer, 1997 1 0
## Mitchell, 2019 1 0
## Murray, 2003 1 0
## Nikolaus, 1999 2 1
## Pinedo-Villanueva, 2019 3 1
## Pitkala, 2013 5 1
## Reuben, 2004 1 0
## Rigaud, 2002 3 0
## Rojas, 2010 4 1
## Ruchlin, 2001 2 1
## Ruikes, 2018 6 1
## Schousboe, 2019 2 0
## Schraeder, 2008 1 0
## Schwardzkopf, 2011 5 1
## Scott, 2004 5 1
## Taylor, 2003 3 0
## Van Lier, 2016 5 1
## Wang, 2008 3 0
## Wang, 2010 1 0
## Witham, 2012 4 1
## Wolff, 2019 3 1
## Woods, 2012 3 1
## Wubker, 2014 1 0
## Zhang, 2007 3 1

table(data3$Study, data3$costs.subtype.2=="Hospital")

##
## FALSE TRUE
## Akerborg, 2016 4 1
## Akincigil, 2020 3 1
## Ankuda, 2017 1 0
## Beech, 1999 2 1
## Beeri, 2002 5 0
## Bleijenberg, 2017 3 1
## Boult, 2001 4 1
## Braithwaite, 2003 1 0
## Brinda, 2014 1 0
## Challis, 2002 7 1
## Challis, 2004 3 0
## Chavan 2020 0 1
## Chen, 2001 1 0
## Chiatti, 2015 6 1
## Chiu, 2000 2 0
## Coleman, 1999 1 0
## Colon-Emeric 2020 0 1
## Davis, 2011 1 0
## Deardorff, 2019 1 0
## Deb, 2018 4 1
## Del Sindaco, 2007 0 1
## Dodel, 2015 1 0
## Eamer, 2018 2 0
## Eamer, 2019 2 0
## Ensrud, 2018 2 0
## Fairhall, 2015 5 1
## Farre, 2016 3 1
## Forster, 2009 2 1
## Graff, 2008 4 1
## Gustavsson, 2010 3 0
## Han,2019 1 0
## Hardy, 2010 2 0
## Harrow, 2004 2 0
## Hay, 2002 2 0
## Hektoen, 2016 5 1
## Hendriks, 2008 6 1
## Hughes, 2000 3 1
## Hui, 1995 1 1
## Joling, 2015 4 0
## Komisar 1997 3 1
## Kramer 1997 2 1
## Kronborg, 2006 5 1
## Ku, 2019 3 0
## Lafortune, 2020 2 1
## Langa, 2004 1 0
## Leeuwan, 2015 6 1
## Lewin, 2013 0 1
## Liotta, 2019 1 1
## Lu 2020 4 0
## Mann, 1999 1 1
## Marshall, 1999 2 1
## Maru,2015 2 1
## Max, 1995 1 0
## McCusker, 2003 3 1
## McNamee, 1999 1 0
## Melin, 1993 2 1
## Metzelthin, 2015 6 1
## Michalowsky, 2016 2 0
## Miller, 2005 3 1
## Mintzer, 1997 1 0
## Mitchell, 2019 1 0
## Murray, 2003 0 1
## Nikolaus, 1999 2 1
## Pinedo-Villanueva, 2019 4 0
## Pitkala, 2013 5 1
## Reuben, 2004 0 1
## Rigaud, 2002 3 0
## Rojas, 2010 4 1
## Ruchlin, 2001 3 0
## Ruikes, 2018 6 1
## Schousboe, 2019 2 0
## Schraeder, 2008 1 0
## Schwardzkopf, 2011 5 1
## Scott, 2004 5 1
## Taylor, 2003 3 0
## Van Lier, 2016 5 1
## Wang, 2008 3 0
## Wang, 2010 1 0
## Witham, 2012 4 1
## Wolff, 2019 4 0
## Woods, 2012 3 1
## Wubker, 2014 1 0
## Zhang, 2007 3 1

table(data3$Study, data3$costs.subtype.2=="Outpatient")

##
## FALSE TRUE
## Akerborg, 2016 4 1
## Akincigil, 2020 3 1
## Ankuda, 2017 1 0
## Beech, 1999 2 1
## Beeri, 2002 5 0
## Bleijenberg, 2017 4 0
## Boult, 2001 4 1
## Braithwaite, 2003 1 0
## Brinda, 2014 1 0
## Challis, 2002 7 1
## Challis, 2004 3 0
## Chavan 2020 1 0
## Chen, 2001 1 0
## Chiatti, 2015 6 1
## Chiu, 2000 2 0
## Coleman, 1999 1 0
## Colon-Emeric 2020 1 0
## Davis, 2011 1 0
## Deardorff, 2019 1 0
## Deb, 2018 4 1
## Del Sindaco, 2007 1 0
## Dodel, 2015 1 0
## Eamer, 2018 2 0
## Eamer, 2019 1 1
## Ensrud, 2018 1 1
## Fairhall, 2015 5 1
## Farre, 2016 3 1
## Forster, 2009 2 1
## Graff, 2008 5 0
## Gustavsson, 2010 3 0
## Han,2019 1 0
## Hardy, 2010 2 0
## Harrow, 2004 2 0
## Hay, 2002 2 0
## Hektoen, 2016 5 1
## Hendriks, 2008 7 0
## Hughes, 2000 3 1
## Hui, 1995 1 1
## Joling, 2015 4 0
## Komisar 1997 3 1
## Kramer 1997 2 1
## Kronborg, 2006 5 1
## Ku, 2019 3 0
## Lafortune, 2020 3 0
## Langa, 2004 1 0
## Leeuwan, 2015 6 1
## Lewin, 2013 1 0
## Liotta, 2019 2 0
## Lu 2020 3 1
## Mann, 1999 2 0
## Marshall, 1999 2 1
## Maru,2015 3 0
## Max, 1995 1 0
## McCusker, 2003 4 0
## McNamee, 1999 1 0
## Melin, 1993 2 1
## Metzelthin, 2015 7 0
## Michalowsky, 2016 2 0
## Miller, 2005 3 1
## Mintzer, 1997 1 0
## Mitchell, 2019 1 0
## Murray, 2003 1 0
## Nikolaus, 1999 3 0
## Pinedo-Villanueva, 2019 3 1
## Pitkala, 2013 5 1
## Reuben, 2004 1 0
## Rigaud, 2002 3 0
## Rojas, 2010 4 1
## Ruchlin, 2001 3 0
## Ruikes, 2018 6 1
## Schousboe, 2019 1 1
## Schraeder, 2008 1 0
## Schwardzkopf, 2011 5 1
## Scott, 2004 5 1
## Taylor, 2003 3 0
## Van Lier, 2016 6 0
## Wang, 2008 3 0
## Wang, 2010 1 0
## Witham, 2012 4 1
## Wolff, 2019 3 1
## Woods, 2012 4 0
## Wubker, 2014 1 0
## Zhang, 2007 3 1

table(data3$Study, data3$costs.subtype.2=="Medications")

##
## FALSE TRUE
## Akerborg, 2016 5 0
## Akincigil, 2020 4 0
## Ankuda, 2017 1 0
## Beech, 1999 3 0
## Beeri, 2002 4 1
## Bleijenberg, 2017 4 0
## Boult, 2001 5 0
## Braithwaite, 2003 1 0
## Brinda, 2014 1 0
## Challis, 2002 8 0
## Challis, 2004 3 0
## Chavan 2020 1 0
## Chen, 2001 1 0
## Chiatti, 2015 6 1
## Chiu, 2000 2 0
## Coleman, 1999 0 1
## Colon-Emeric 2020 1 0
## Davis, 2011 1 0
## Deardorff, 2019 1 0
## Deb, 2018 4 1
## Del Sindaco, 2007 1 0
## Dodel, 2015 1 0
## Eamer, 2018 2 0
## Eamer, 2019 2 0
## Ensrud, 2018 2 0
## Fairhall, 2015 6 0
## Farre, 2016 3 1
## Forster, 2009 2 1
## Graff, 2008 5 0
## Gustavsson, 2010 3 0
## Han,2019 1 0
## Hardy, 2010 2 0
## Harrow, 2004 2 0
## Hay, 2002 2 0
## Hektoen, 2016 6 0
## Hendriks, 2008 6 1
## Hughes, 2000 4 0
## Hui, 1995 2 0
## Joling, 2015 3 1
## Komisar 1997 4 0
## Kramer 1997 3 0
## Kronborg, 2006 5 1
## Ku, 2019 3 0
## Lafortune, 2020 3 0
## Langa, 2004 1 0
## Leeuwan, 2015 6 1
## Lewin, 2013 1 0
## Liotta, 2019 2 0
## Lu 2020 4 0
## Mann, 1999 2 0
## Marshall, 1999 3 0
## Maru,2015 2 1
## Max, 1995 1 0
## McCusker, 2003 3 1
## McNamee, 1999 1 0
## Melin, 1993 3 0
## Metzelthin, 2015 6 1
## Michalowsky, 2016 2 0
## Miller, 2005 4 0
## Mintzer, 1997 1 0
## Mitchell, 2019 1 0
## Murray, 2003 1 0
## Nikolaus, 1999 3 0
## Pinedo-Villanueva, 2019 3 1
## Pitkala, 2013 6 0
## Reuben, 2004 1 0
## Rigaud, 2002 3 0
## Rojas, 2010 4 1
## Ruchlin, 2001 3 0
## Ruikes, 2018 6 1
## Schousboe, 2019 2 0
## Schraeder, 2008 1 0
## Schwardzkopf, 2011 5 1
## Scott, 2004 5 1
## Taylor, 2003 2 1
## Van Lier, 2016 6 0
## Wang, 2008 2 1
## Wang, 2010 1 0
## Witham, 2012 4 1
## Wolff, 2019 4 0
## Woods, 2012 4 0
## Wubker, 2014 1 0
## Zhang, 2007 4 0

table(data3$Study, data3$costs.subtype.2=="Home-Help Indirect")

##
## FALSE TRUE
## Akerborg, 2016 4 1
## Akincigil, 2020 4 0
## Ankuda, 2017 1 0
## Beech, 1999 3 0
## Beeri, 2002 4 1
## Bleijenberg, 2017 3 1
## Boult, 2001 5 0
## Braithwaite, 2003 1 0
## Brinda, 2014 0 1
## Challis, 2002 8 0
## Challis, 2004 3 0
## Chavan 2020 1 0
## Chen, 2001 1 0
## Chiatti, 2015 6 1
## Chiu, 2000 1 1
## Coleman, 1999 1 0
## Colon-Emeric 2020 1 0
## Davis, 2011 1 0
## Deardorff, 2019 1 0
## Deb, 2018 5 0
## Del Sindaco, 2007 1 0
## Dodel, 2015 1 0
## Eamer, 2018 2 0
## Eamer, 2019 2 0
## Ensrud, 2018 2 0
## Fairhall, 2015 6 0
## Farre, 2016 3 1
## Forster, 2009 3 0
## Graff, 2008 5 0
## Gustavsson, 2010 2 1
## Han,2019 1 0
## Hardy, 2010 2 0
## Harrow, 2004 1 1
## Hay, 2002 1 1
## Hektoen, 2016 6 0
## Hendriks, 2008 6 1
## Hughes, 2000 4 0
## Hui, 1995 2 0
## Joling, 2015 3 1
## Komisar 1997 4 0
## Kramer 1997 3 0
## Kronborg, 2006 6 0
## Ku, 2019 3 0
## Lafortune, 2020 3 0
## Langa, 2004 0 1
## Leeuwan, 2015 6 1
## Lewin, 2013 1 0
## Liotta, 2019 2 0
## Lu 2020 4 0
## Mann, 1999 2 0
## Marshall, 1999 3 0
## Maru,2015 3 0
## Max, 1995 0 1
## McCusker, 2003 4 0
## McNamee, 1999 1 0
## Melin, 1993 3 0
## Metzelthin, 2015 6 1
## Michalowsky, 2016 1 1
## Miller, 2005 4 0
## Mintzer, 1997 1 0
## Mitchell, 2019 1 0
## Murray, 2003 1 0
## Nikolaus, 1999 3 0
## Pinedo-Villanueva, 2019 3 1
## Pitkala, 2013 5 1
## Reuben, 2004 1 0
## Rigaud, 2002 2 1
## Rojas, 2010 5 0
## Ruchlin, 2001 2 1
## Ruikes, 2018 6 1
## Schousboe, 2019 2 0
## Schraeder, 2008 1 0
## Schwardzkopf, 2011 5 1
## Scott, 2004 6 0
## Taylor, 2003 2 1
## Van Lier, 2016 5 1
## Wang, 2008 2 1
## Wang, 2010 0 1
## Witham, 2012 5 0
## Wolff, 2019 4 0
## Woods, 2012 4 0
## Wubker, 2014 1 0
## Zhang, 2007 4 0

table(data3$Study, data3$costs.subtype.2=="Direct Healthcare Related Costs")

##
## FALSE TRUE
## Akerborg, 2016 5 0
## Akincigil, 2020 4 0
## Ankuda, 2017 1 0
## Beech, 1999 3 0
## Beeri, 2002 4 1
## Bleijenberg, 2017 4 0
## Boult, 2001 5 0
## Braithwaite, 2003 1 0
## Brinda, 2014 1 0
## Challis, 2002 8 0
## Challis, 2004 3 0
## Chavan 2020 1 0
## Chen, 2001 1 0
## Chiatti, 2015 7 0
## Chiu, 2000 1 1
## Coleman, 1999 1 0
## Colon-Emeric 2020 1 0
## Davis, 2011 1 0
## Deardorff, 2019 1 0
## Deb, 2018 5 0
## Del Sindaco, 2007 1 0
## Dodel, 2015 1 0
## Eamer, 2018 1 1
## Eamer, 2019 2 0
## Ensrud, 2018 2 0
## Fairhall, 2015 6 0
## Farre, 2016 4 0
## Forster, 2009 3 0
## Graff, 2008 4 1
## Gustavsson, 2010 3 0
## Han,2019 1 0
## Hardy, 2010 2 0
## Harrow, 2004 2 0
## Hay, 2002 2 0
## Hektoen, 2016 5 1
## Hendriks, 2008 6 1
## Hughes, 2000 4 0
## Hui, 1995 2 0
## Joling, 2015 4 0
## Komisar 1997 4 0
## Kramer 1997 3 0
## Kronborg, 2006 6 0
## Ku, 2019 3 0
## Lafortune, 2020 3 0
## Langa, 2004 1 0
## Leeuwan, 2015 7 0
## Lewin, 2013 1 0
## Liotta, 2019 2 0
## Lu 2020 4 0
## Mann, 1999 2 0
## Marshall, 1999 3 0
## Maru,2015 3 0
## Max, 1995 1 0
## McCusker, 2003 3 1
## McNamee, 1999 1 0
## Melin, 1993 3 0
## Metzelthin, 2015 7 0
## Michalowsky, 2016 2 0
## Miller, 2005 4 0
## Mintzer, 1997 1 0
## Mitchell, 2019 1 0
## Murray, 2003 1 0
## Nikolaus, 1999 3 0
## Pinedo-Villanueva, 2019 4 0
## Pitkala, 2013 6 0
## Reuben, 2004 1 0
## Rigaud, 2002 2 1
## Rojas, 2010 5 0
## Ruchlin, 2001 3 0
## Ruikes, 2018 7 0
## Schousboe, 2019 2 0
## Schraeder, 2008 1 0
## Schwardzkopf, 2011 6 0
## Scott, 2004 6 0
## Taylor, 2003 3 0
## Van Lier, 2016 6 0
## Wang, 2008 3 0
## Wang, 2010 1 0
## Witham, 2012 5 0
## Wolff, 2019 4 0
## Woods, 2012 4 0
## Wubker, 2014 1 0
## Zhang, 2007 4 0

data4<-data3 %>%
 mutate(Healthcare_All = ifelse(Cost.Perspective == "Healthcare" & costs.subtype.2 == "All", Annual.Costs.mean, NA),
 Societal_All = ifelse(Cost.Perspective == "Societal" & costs.subtype.2 == "All", Annual.Costs.mean, NA),
 Personal_All = ifelse(Cost.Perspective == "Personal" & costs.subtype.2 == "All", Annual.Costs.mean, NA),
 Healthcare_Clinician = ifelse(Cost.Perspective == "Healthcare" & costs.subtype.2 == "Clinician", Annual.Costs.mean, NA),
 Healthcare_Hospital = ifelse(Cost.Perspective == "Healthcare" & costs.subtype.2 == "Hospital", Annual.Costs.mean, NA),
 Healthcare_Outpatient = ifelse(Cost.Perspective == "Healthcare" & costs.subtype.2 == "Outpatient", Annual.Costs.mean, NA),
 Societal_Assisted = ifelse(Cost.Perspective == "Societal" & costs.subtype.2 == "Assisted Services", Annual.Costs.mean, NA),
 Societal_HomeHealth = ifelse(Cost.Perspective == "Societal" & costs.subtype.2 == "Home Healthcare", Annual.Costs.mean, NA),
 Personal_HealthCosts= ifelse(Cost.Perspective == "Personal" & costs.subtype.2 == "Direct Healthcare Related Costs", Annual.Costs.mean, NA),
 Personal_HomeHelp = ifelse(Cost.Perspective == "Personal" & costs.subtype.2 == "Home-Help Indirect", Annual.Costs.mean, NA),
 Personal_Meds = ifelse(Cost.Perspective == "Personal" & costs.subtype.2 == "Medications", Annual.Costs.mean, NA),
 Healthcare_All_lib_SD = ifelse(Cost.Perspective == "Healthcare" & costs.subtype.2 == "All", Annual.Costs.sd.liberal, NA),
 Societal_All_lib_SD = ifelse(Cost.Perspective == "Societal" & costs.subtype.2 == "All", Annual.Costs.sd.liberal, NA),
 Personal_All_lib_SD = ifelse(Cost.Perspective == "Personal" & costs.subtype.2 == "All", Annual.Costs.sd.liberal, NA),
 Healthcare_Clinician_lib_SD = ifelse(Cost.Perspective == "Healthcare" & costs.subtype.2 == "Clinician", Annual.Costs.sd.liberal, NA),
 Healthcare_Hospital_lib_SD = ifelse(Cost.Perspective == "Healthcare" & costs.subtype.2 == "Hospital", Annual.Costs.sd.liberal, NA),
 Healthcare_Outpatient_lib_SD = ifelse(Cost.Perspective == "Healthcare" & costs.subtype.2 == "Outpatient", Annual.Costs.sd.liberal, NA),
 Societal_Assisted_lib_SD = ifelse(Cost.Perspective == "Societal" & costs.subtype.2 == "Assisted Services", Annual.Costs.sd.liberal, NA),
 Societal_HomeHealth_lib_SD = ifelse(Cost.Perspective == "Societal" & costs.subtype.2 == "Home Healthcare", Annual.Costs.sd.liberal, NA),
 Personal_HealthCosts_lib_SD = ifelse(Cost.Perspective == "Personal" & costs.subtype.2 == "Direct Healthcare Related Costs", Annual.Costs.sd.liberal, NA),
 Personal_HomeHelp_lib_SD = ifelse(Cost.Perspective == "Personal" & costs.subtype.2 == "Home-Help Indirect", Annual.Costs.sd.liberal, NA),
 Personal_Meds_lib_SD = ifelse(Cost.Perspective == "Personal" & costs.subtype.2 == "Medications", Annual.Costs.sd.liberal, NA),
 Healthcare_All_con_SD = ifelse(Cost.Perspective == "Healthcare" & costs.subtype.2 == "All", Annual.Costs.sd.conservative, NA),
 Societal_All_con_SD = ifelse(Cost.Perspective == "Societal" & costs.subtype.2 == "All", Annual.Costs.sd.conservative, NA),
 Personal_All_con_SD = ifelse(Cost.Perspective == "Personal" & costs.subtype.2 == "All", Annual.Costs.sd.conservative, NA),
 Healthcare_Clinician_con_SD = ifelse(Cost.Perspective == "Healthcare" & costs.subtype.2 == "Clinician", Annual.Costs.sd.conservative, NA),
 Healthcare_Hospital_con_SD = ifelse(Cost.Perspective == "Healthcare" & costs.subtype.2 == "Hospital", Annual.Costs.sd.conservative, NA),
 Healthcare_Outpatient_con_SD = ifelse(Cost.Perspective == "Healthcare" & costs.subtype.2 == "Outpatient", Annual.Costs.sd.conservative, NA),
 Societal_Assisted_con_SD = ifelse(Cost.Perspective == "Societal" & costs.subtype.2 == "Assisted Services", Annual.Costs.sd.conservative, NA),
 Societal_HomeHealth_con_SD = ifelse(Cost.Perspective == "Societal" & costs.subtype.2 == "Home Healthcare", Annual.Costs.sd.conservative, NA),
 Personal_HealthCosts_con_SD = ifelse(Cost.Perspective == "Personal" & costs.subtype.2 == "Direct Healthcare Related Costs", Annual.Costs.sd.conservative, NA),
 Personal_HomeHelp_con_SD = ifelse(Cost.Perspective == "Personal" & costs.subtype.2 == "Home-Help Indirect", Annual.Costs.sd.conservative, NA),
 Personal_Meds_con_SD = ifelse(Cost.Perspective == "Personal" & costs.subtype.2 == "Medications", Annual.Costs.sd.conservative, NA),)

data5<-transform(data4, Healthcare_All = as.numeric(Healthcare_All),
 Societal_All = as.numeric(Societal_All),
 Personal_All = as.numeric(Personal_All),
 Healthcare_Clinician = as.numeric(Healthcare_Clinician),
 Healthcare_Hospital = as.numeric(Healthcare_Hospital),
 Healthcare_Outpatient = as.numeric(Healthcare_Outpatient),
 Societal_Assisted = as.numeric(Societal_Assisted),
 Societal_HomeHealth = as.numeric(Societal_HomeHealth),
 Personal_HealthCosts = as.numeric(Personal_HealthCosts),
 Personal_HomeHelp = as.numeric(Personal_HomeHelp),
 Personal_Meds = as.numeric(Personal_Meds),
 Healthcare_All_lib_SD = as.numeric(Healthcare_All_lib_SD),
 Societal_All_lib_SD = as.numeric(Societal_All_lib_SD),
 Personal_All_lib_SD = as.numeric(Personal_All_lib_SD),
 Healthcare_Clinician_lib_SD = as.numeric(Healthcare_Clinician_lib_SD),
 Healthcare_Hospital_lib_SD = as.numeric(Healthcare_Hospital_lib_SD),
 Healthcare_Outpatient_lib_SD = as.numeric(Healthcare_Outpatient_lib_SD),
 Societal_Assisted_lib_SD = as.numeric(Societal_Assisted_lib_SD),
 Societal_HomeHealth_lib_SD = as.numeric(Societal_HomeHealth_lib_SD),
 Personal_HealthCosts_lib_SD = as.numeric(Personal_HealthCosts_lib_SD),
 Personal_HomeHelp_lib_SD = as.numeric(Personal_HomeHelp_lib_SD),
 Personal_Meds_lib_SD = as.numeric(Personal_Meds_lib_SD),
 Healthcare_All_con_SD = as.numeric(Healthcare_All_con_SD),
 Societal_All_con_SD = as.numeric(Societal_All_con_SD),
 Personal_All_con_SD = as.numeric(Personal_All_con_SD),
 Healthcare_Clinician_con_SD = as.numeric(Healthcare_Clinician_con_SD),
 Healthcare_Hospital_con_SD = as.numeric(Healthcare_Hospital_con_SD),
 Healthcare_Outpatient_con_SD = as.numeric(Healthcare_Outpatient_con_SD),
 Societal_Assisted_con_SD = as.numeric(Societal_Assisted_con_SD),
 Societal_HomeHealth_con_SD = as.numeric(Societal_HomeHealth_con_SD),
 Personal_HealthCosts_con_SD = as.numeric(Personal_HealthCosts_con_SD),
 Personal_HomeHelp_con_SD = as.numeric(Personal_HomeHelp_con_SD),
 Personal_Meds_con_SD = as.numeric(Personal_Meds_con_SD))

data5$Study_Design<-NA
data5$Study_Design[data5$Study.Design == "Observational"]<-"Observational"
data5$Study_Design[data5$Study.Design == "RCT" | data5$Study.Design == "RCT "] <- "RCT"
data5$Study_Design[data5$Study.Design == "Computer Simulation" |data5$Study.Design == "Cost-effectiveness study" | data5$Study.Design == "Quasi-Experimental"] <- "Other"

#COST ANALYSIS RESULTS

CI.intvl <- function(x){
 avg <- as.numeric(mean(x, na.rm=TRUE))
 std.dev<- as.numeric(sd(x, na.rm = TRUE))
 number<- as.numeric(length(x[!is.na(x)]))
 std.err<- as.numeric(std.dev/sqrt(number))
 UL.CI<- avg + 1.96*std.err
 LL.CI<- avg - 1.96*std.err
 return(list(study_number = number, Mean=avg, Upper_Limit=UL.CI, Lower_Limit=LL.CI))
}

#Healthcare Means
CI.intvl(data5$Healthcare_All)

## $study_number
## [1] 44
##
## $Mean
## [1] 24258.69
##
## $Upper_Limit
## [1] 39103.2
##
## $Lower_Limit
## [1] 9414.183

CI.intvl(data5$Healthcare_Clinician)

## $study_number
## [1] 28
##
## $Mean
## [1] 7615.353
##
## $Upper_Limit
## [1] 17888.65
##
## $Lower_Limit
## [1] -2657.949

CI.intvl(data5$Healthcare_Hospital)

## $study_number
## [1] 45
##
## $Mean
## [1] 16776.03
##
## $Upper_Limit
## [1] 28227.76
##
## $Lower_Limit
## [1] 5324.29

CI.intvl(data5$Healthcare_Outpatient)

## $study_number
## [1] 33
##
## $Mean
## [1] 9410.797
##
## $Upper_Limit
## [1] 17976.51
##
## $Lower_Limit
## [1] 845.085

#Healthcare SDs
CI.intvl(data5$Healthcare_All_lib_SD)

## $study_number
## [1] 44
##
## $Mean
## [1] 23150.11
##
## $Upper_Limit
## [1] 31022.09
##
## $Lower_Limit
## [1] 15278.12

CI.intvl(data5$Healthcare_Clinician_lib_SD)

## $study_number
## [1] 28
##
## $Mean
## [1] 7660.119
##
## $Upper_Limit
## [1] 17848.24
##
## $Lower_Limit
## [1] -2528.002

CI.intvl(data5$Healthcare_Hospital_lib_SD)

## $study_number
## [1] 45
##
## $Mean
## [1] 18564.66
##
## $Upper_Limit
## [1] 30065.34
##
## $Lower_Limit
## [1] 7063.981

CI.intvl(data5$Healthcare_Outpatient_lib_SD)

## $study_number
## [1] 33
##
## $Mean
## [1] 10678.69
##
## $Upper_Limit
## [1] 19602.46
##
## $Lower_Limit
## [1] 1754.908

#Societal Means
CI.intvl(data5$Societal_All)

## $study_number
## [1] 14
##
## $Mean
## [1] 27380.74
##
## $Upper_Limit
## [1] 50685.96
##
## $Lower_Limit
## [1] 4075.526

CI.intvl(data5$Societal_HomeHealth)

## $study_number
## [1] 20
##
## $Mean
## [1] 3062.639
##
## $Upper_Limit
## [1] 4123.548
##
## $Lower_Limit
## [1] 2001.731

CI.intvl(data5$Societal_Assisted)

## $study_number
## [1] 15
##
## $Mean
## [1] 3374.485
##
## $Upper_Limit
## [1] 6712.912
##
## $Lower_Limit
## [1] 36.05761

#Societal SDs
CI.intvl(data5$Societal_All_lib_SD)

## $study_number
## [1] 14
##
## $Mean
## [1] 33249.11
##
## $Upper_Limit
## [1] 59269.99
##
## $Lower_Limit
## [1] 7228.237

CI.intvl(data5$Societal_HomeHealth_lib_SD)

## $study_number
## [1] 20
##
## $Mean
## [1] 5133.308
##
## $Upper_Limit
## [1] 7097.898
##
## $Lower_Limit
## [1] 3168.718

CI.intvl(data5$Societal_Assisted_lib_SD)

## $study_number
## [1] 15
##
## $Mean
## [1] 4551.32
##
## $Upper_Limit
## [1] 8410.729
##
## $Lower_Limit
## [1] 691.9106

#Personal Means
CI.intvl(data5$Personal_All)

## $study_number
## [1] 9
##
## $Mean
## [1] 7455.493
##
## $Upper_Limit
## [1] 12639.53
##
## $Lower_Limit
## [1] 2271.452

CI.intvl(data5$Personal_HealthCosts)

## $study_number
## [1] 8
##
## $Mean
## [1] 3929.457
##
## $Upper_Limit
## [1] 7077.727
##
## $Lower_Limit
## [1] 781.1883

CI.intvl(data5$Personal_HomeHelp)

## $study_number
## [1] 27
##
## $Mean
## [1] 14355.82
##
## $Upper_Limit
## [1] 21369.06
##
## $Lower_Limit
## [1] 7342.575

CI.intvl(data5$Personal_Meds)

## $study_number
## [1] 21
##
## $Mean
## [1] 1136.737
##
## $Upper_Limit
## [1] 1558.327
##
## $Lower_Limit
## [1] 715.1468

#Personal SDs
CI.intvl(data5$Personal_All_lib_SD)

## $study_number
## [1] 9
##
## $Mean
## [1] 11208.48
##
## $Upper_Limit
## [1] 17154.87
##
## $Lower_Limit
## [1] 5262.092

CI.intvl(data5$Personal_HealthCosts_lib_SD)

## $study_number
## [1] 8
##
## $Mean
## [1] 4602.576
##
## $Upper_Limit
## [1] 7512.385
##
## $Lower_Limit
## [1] 1692.767

CI.intvl(data5$Personal_HomeHelp_lib_SD)

## $study_number
## [1] 27
##
## $Mean
## [1] 16197.78
##
## $Upper_Limit
## [1] 23178.32
##
## $Lower_Limit
## [1] 9217.229

CI.intvl(data5$Personal_Meds_lib_SD)

## $study_number
## [1] 21
##
## $Mean
## [1] 1511.597
##
## $Upper_Limit
## [1] 2142.219
##
## $Lower_Limit
## [1] 880.9745
