# Supplementary material for: International depiction of the cost of functional independence limitations among older adults living in the community: a systematic review and cost-of-impairment study
Source: BMC Geriatr. 2022 Oct 22;22:815. doi: 10.1186/s12877-022-03466-w (PMC9587635; doi:10.1186/s12877-022-03466-w)
Supplement: Supplementary file 3 — Additional file 3: Supplementary Material S2. Cost estimates for each study. [file 12877_2022_3466_MOESM3_ESM.pdf]

Study	Cost persective	Cost Subtype	Costs mean	Costs SD	Sensitivity Analysis	Comments
Akerborg, 2016	Healthcare	Hospital	2908.73	6830.25	0	
Akerborg, 2016	Healthcare	Outpatient	2608.01	5958.32	0	
Akerborg, 2016	Personal	Home-Help Indirect	3459.2	5615.25	0	
Akerborg, 2016	Societal	Assisted Services	25539.13	29883.21	0	
Akerborg, 2016	Societal	Home Healthcare	2868.12	5539.21	0	
Akerborg, 2016	Total	All	37382.32	30271.22	0	
Akincigil, 2020	Healthcare	All	15515.91		0	
Akincigil, 2020	Healthcare	Hospital	4771.79		0	
Akincigil, 2020	Healthcare	Outpatient	10034.14		0	
Akincigil, 2020	Societal	Home Healthcare	711.1		0	
Ankuda, 2017	Healthcare	All	10003.28	258.34	0	
Beech, 1999	Healthcare	All	8215.36		1	
Beech, 1999	Healthcare	Hospital	6468.04		1	
Beech, 1999	Healthcare	Outpatient	1747.32		1	
Beeri, 2002	Healthcare	Clinician	41.63		0	
Beeri, 2002	Personal	All	22740.33		0	
Beeri, 2002	Personal	Direct Health Care Related Costs	6150.3		0	
Beeri, 2002	Personal	Home-Help Indirect	16590.03			
Beeri, 2002	Personal	Medications	791.02		0	
Bleijenberg, 2017	Healthcare	All	4423.01	8995.24	1	
Bleijenberg, 2017	Healthcare	Hospital	153070.05		1	
Bleijenberg, 2017	Personal	Home-Help Indirect	1602.43	6597.22	1	
Bleijenberg, 2017	Societal	Assisted Services	0		1	
Boult, 2001	Healthcare	All	18842.23	30918.68	0	
Boult, 2001	Healthcare	Clinician	3693.53	4515.95	0	
Boult, 2001	Healthcare	Hospital	10697.9	22185.63	0	
Boult, 2001	Healthcare	Outpatient	1226.29	2032.42	0	
Boult, 2001	Societal	Home Healthcare	1278.41	4840.03	0	
Braithwaite, 2003	Healthcare	All	39804.78		1	
Brinda, 2014	Personal	Home-Help Indirect	5337.04	2127.32	0	
Challis, 2002	Healthcare	All	6034.99		0	
Challis, 2002	Healthcare	Clinician	2040.85		0	
Challis, 2002	Healthcare	Hospital	4365.57		0	
Challis, 2002	Healthcare	Outpatient	544.27		0	

Challis, 2002	Personal	All	3554.61		0	
Challis, 2002	Societal	All	24297.34		0	
Challis, 2002	Societal	Assisted Services	9377.45		0	
Challis, 2002	Societal	Home Healthcare	4029.05		0	
Challis, 2004	Healthcare	All	84.33		0	
Challis, 2004	Personal	All	110.82		0	
Challis, 2004	Societal	All	197.31		0	
Chavan 2020	Healthcare	Hospital	23012.66	2100.4	0	Data from 2008-2011 used, but assumed questions about Medicare usage were for 1 year
Chen, 2001	Healthcare	All	83460.03		1	Average sum of Home, Home care and Rehab across each frailty condition
Chiatti, 2015	Healthcare	All	1874.3	1993.33	0	
Chiatti, 2015	Healthcare	Hospital	292.68	1445.98	0	Aggregate sum of hospitalizations and emergency costs
Chiatti, 2015	Healthcare	Outpatient	16.54	20.93	0	
Chiatti, 2015	Personal	All	1229.42	2253.76	0	
Chiatti, 2015	Personal	Home-Help Indirect	5617.94	4886.37	0	
Chiatti, 2015	Personal	Medications	579.57	1128.21	0	
Chiatti, 2015	Societal	All	8320.67	5531.62	0	
Chiu, 2000	Personal	Direct Health Care Related Costs	2943.41		0	
Chiu, 2000	Personal	Home-Help Indirect	12726.66		0	
Coleman, 1999	Personal	Medications	1555.72		0	
Coleman, 1999	Total	All	16086.98		0	
Colon-Emeric 2020	Healthcare	Hospital	9414.54	904.54	0	
Davis, 2011	Healthcare	All	1191.67	1911.44	1	
Deardorff, 2019	Healthcare	All	17034.29	39604.73	0	
Deb, 2018	Healthcare	All	15248.12	20930.58	0	
Deb, 2018	Healthcare	Hospital	3962.8	8438.97	0	
Deb, 2018	Healthcare	Outpatient	3193.2	5283.27	0	
Deb, 2018	Personal	Medications	3512.74	3922.7	0	
Deb, 2018	Societal	Home Healthcare	3535.24	16906.51	0	
Del Sindaco, 2007	Healthcare	Hospital	4091.77		0	
Dodel, 2015	Societal	All	2166.17		1	
Eamer, 2018	Healthcare	Clinician	125.06	179.23	0	Assumed median was mean; SD = IQR/1.35
Eamer, 2018	Personal	Direct Health Care Related Costs	0	249.04	0	Assumed median was mean; SD = IQR/1.35

Eamer, 2018	Total	All	449.48	1213.64	0	Assumed median was mean; SD = IQR/1.35
Eamer, 2019	Healthcare	Outpatient	30989.54	39091.03	0	
Eamer, 2019	Personal	All	2743.09	7319.43	0	
Eamer, 2019	Total	All	36610.74	41383.73	0	
Ensrud, 2018	Healthcare	All	7399.21	13808.18	0	
Ensrud, 2018	Healthcare	Outpatient	2588.62	3774.93	0	
Fairhall, 2015	Healthcare	Clinician	787.97	1535.44	0	
Fairhall, 2015	Healthcare	Hospital	15118.52	22802.74	0	
Fairhall, 2015	Healthcare	Outpatient	837.02	587.08	0	
Fairhall, 2015	Societal	All	3135.51	3904.01	0	
Fairhall, 2015	Societal	Assisted Services	163.9	470.68	0	
Fairhall, 2015	Societal	Home Healthcare	996.25	1756.19	0	
Farre, 2016	Healthcare	Hospital	105.39	474.32	1	Because estimates were stratified by MMSE and ADL, average costs were calculated for each index and then averaged together
Farre, 2016	Healthcare	Outpatient	75.45	177.37	1	Because estimates were stratified by MMSE and ADL, average costs were calculated for each index and then averaged together
Farre, 2016	Personal	Home-Help Indirect	1070.58	784.52	1	Because estimates were stratified by MMSE and ADL, average costs were calculated for each index and then averaged together
Farre, 2016	Personal	Medications	42.75	53.5	1	Because estimates were stratified by MMSE and ADL, average costs were calculated for each index and then averaged together
Farre, 2016	Total	All	1739.43	1279.28	1	Because estimates were stratified by MMSE and ADL, average costs were calculated for each index and then averaged together
Forster, 2009	Healthcare	Hospital	1353.5	3629.37	0	
Forster, 2009	Healthcare	Outpatient	837.93	659.12	0	
Forster, 2009	Personal	Medications	12.91	6.46	0	

Forster, 2009	Total	All	3040.78	4027.23	0	
Graff, 2008	Healthcare	Clinician	205.03	411.79	1	Aggregated physiotherapy, GP, and hospital specialist costs
Graff, 2008	Healthcare	Hospital	998.56	4251.2	1	
Graff, 2008	Personal	Direct Health Care Related Costs	529.6	1181.63	1	
Graff, 2008	Societal	Assisted Services	779.09	1835.39	1	
Graff, 2008	Societal	Home Healthcare	1987.28	2539.98	1	
Graff, 2008	Total	All	15520.57	8351.68	1	
Gustavsson, 2010	Healthcare	All	1353.98		0	
Gustavsson, 2010	Personal	Home-Help Indirect	241.1		0	
Gustavsson, 2010	Societal	All	2538.71		0	
Gustavsson, 2010	Total	All	8462.37	12843.96	0	
Han,2019	Healthcare	All	1735.24		0	
Hardy, 2010	Healthcare	All	16736.28	65355.95	0	
Hardy, 2010	Personal	All	2676.09	11730.59	0	
Harrow, 2004	Healthcare	All	13132.56		0	
Harrow, 2004	Personal	Home-Help Indirect	38166.51		0	
Hay, 2002	Healthcare	All	3803.55	8735.18	0	
Hay, 2002	Personal	Home-Help Indirect	3848.36	12711.98	0	
Hay, 2002	Total	All	8353.6	17939.25	0	
Hektoen, 2016	Healthcare	Clinician	770.56	670.56	0	
Hektoen, 2016	Healthcare	Hospital	17721.95	15425.99	0	
Hektoen, 2016	Healthcare	Outpatient	10640.61	14689.33	0	Aggregated outpatient and rehab costs; SD is sum of both
Hektoen, 2016	Personal	Direct Health Care Related Costs	320.79	320.79	0	
Hektoen, 2016	Social	Assisted Services	4181.01	7607.99	0	Aggregated meals on wheels and day care centre into "Assisted Services"; SD is sum of SD's for MOW and day care
Hektoen, 2016	Social	Home Healthcare	2114.07	4610.1	0	
Hektoen, 2016	Total	All	56531.64	40580.65	0	
Hendriks, 2008	Healthcare	All	4719.28	5768.53	1	
Hendriks, 2008	Healthcare	Clinician	2137.59	4883.29	1	

Hendriks, 2008	Healthcare	Hospital	1773.43	2719.66	1	
Hendriks, 2008	Patient	All	1112.02	2177.46	1	
Hendriks, 2008	Patient	Direct Health Care Related Costs	654.31	1810.73	1	Paid domestic help and home modifications aggregated
Hendriks, 2008	Patient	Home-Help Indirect	457.72	1507.56	1	
Hendriks, 2008	Patient	Medications	508.64	862.73	1	Medical devices, and medications combined
Hendriks, 2008	Total	All	5831.3	6694.03	1	
Hughes, 2000	Healthcare	All	49847.58	53059.49	1	
Hughes, 2000	Healthcare	Hospital	24984.62	48807.15	1	
Hughes, 2000	Healthcare	Outpatient	7057.32	11920.5	1	
Hughes, 2000	Societal	Home Healthcare	2936.7	8482.04	1	
Hui, 1995	Healthcare	Hospital	605.52		0	
Hui, 1995	Healthcare	Outpatient	351.81		0	
Hui, 1995	Total	All	16732.41	7449.73	0	
Joling, 2015	Healthcare	All	25265.19	36240.6	1	1) Ambulatory and 2) day treatment and admissions aggregated to ALL
Joling, 2015	Personal	Home-Help Indirect	48169.52	54261.41	1	
Joling, 2015	Personal	Medications	1091.59	3308.08	1	
Joling, 2015	Societal	All	2804.29	1420.97	1	
Joling, 2015	Total	All	77330.6	54260.72	1	
Kehusmaa, 2013	Total	All	37301.88		0	Aggregated formal care, informal care, and combination of formal and informal care
Komisar 1997	Healthcare	Clinician	3902.46		0	
Komisar 1997	Healthcare	Hospital	11613.58		0	
Komisar 1997	Healthcare	Outpatient	975.62		0	
Komisar 1997	Societal	Home Healthcare	4521.6		0	
Komisar 1997	Total	All	22101.45		0	
Kramer 1997	Healthcare	All	38821.84		0	
Kramer 1997	Healthcare	Hospital	6021.31		0	
Kramer 1997	Healthcare	Outpatient	23528.91		0	

Kronborg, 2006	Healthcare	Clinical	138.68	355.62	1	Aggregated medical specialists, GP, and physio into Clinical
Kronborg, 2006	Healthcare	Hospital	1039.99	3223.87	1	
Kronborg, 2006	Healthcare	Outpatient	18.96	198.85	1	
Kronborg, 2006	Personal	Medications	17.08	114.46	1	
Kronborg, 2006	Societal	Assisted Services	137.83	1115.44	1	Aggregated day care and MOW into Assisted Services
Kronborg, 2006	Societal	Home Healthcare	580.81	3013.47	1	
Kronborg, 2006	Total	All	2546.34	6973.73	1	
Ku, 2019	Healthcare	All	12029.87	15432.34	0	
Ku, 2019	Personal	All	19553.28	28224.19	0	
Ku, 2019	Societal	All	17124.45	24816.19	0	
Ku, 2019	Total	All	48707.59	40922.38	0	
Lafortune, 2020	Healthcare	Clinical	64.29	28.78	1	GP visits and Formal care aggregated to Clinician
Lafortune, 2020	Healthcare	Hospital	42.9	16.24	1	
Lafortune, 2020	Societal	All	25.6	8	1	
Lafortune, 2020	Total	All	87.52	28.87	1	
Langa, 2004	Personal	Home-Help Indirect	2890.84	10847.18	1	Aggregated across all depressive symptom levels
Leeuwan, 2015	Healthcare	Clinical	505.33	1055.99	1	
Leeuwan, 2015	Healthcare	Hospital	887.7	3227.56	1	
Leeuwan, 2015	Healthcare	Outpatient	708.87	2012.4	1	
Leeuwan, 2015	Personal	Home-Help Indirect	2771.93	7571.05	1	
Leeuwan, 2015	Personal	Medications	897.11	2789.32	1	
Leeuwan, 2015	Societal	Assisted Services	456.5	1574.16	1	
Leeuwan, 2015	Societal	Home Healthcare	2727.22	5797.76	1	
Lewin, 2013	Healthcare	Hospital	12125.04		0	
Lewin, 2013	Total	All	19119.66		0	
Liotta, 2019	Healthcare	All	5536.57		0	
Liotta, 2019	Healthcare	Hospital	4506.66		0	
Lu 2020	Healthcare	All	4714	27796	0	
Lu 2020	Healthcare	Outpatient	215	1846	0	
Lu 2020	Healthcare	Clinician	148	969	0	
Lu 2020	Societal	Assisted Services	91	1817	0	
Mann, 1999	Healthcare	All	39461.62	48268.98	0	
Mann, 1999	Healthcare	Hospital	22258.04	45165.98	0	
Marshall, 1999	Healthcare	All	8256.16		0	
Marshall, 1999	Healthcare	Hospital	7160.73		0	
Marshall, 1999	Healthcare	Outpatient	3842.01		0	

Maru,2015	Healthcare	Hospital	6916.37	17123.91	1	
Maru,2015	Healthcare	Total	7740.92	14710.15	1	
Maru,2015	Personal	Medications	433.86	430.35	1	
Max, 1995	Personal	Home-Help Indirect	70199.29		0	
McCusker, 2003	Healthcare	All	5204.54	6303.71	0	Aggregated public and private healthcare costs
McCusker, 2003	Healthcare	Hospital	1716.97	3957.7	0	
McCusker, 2003	Personal	Direct Health Care Related Costs	397.3	771.41	0	Aggregated out-of-pocket costs for patient and caregiver
McCusker, 2003	Personal	Medications	1127.65	974.03	0	
McNamee, 1999	Societal	All	3378.56		0	
Melin, 1993	Healthcare	All	37114.28		0	
Melin, 1993	Healthcare	Hospital	28089.92		0	
Melin, 1993	Healthcare	Outpatient	7372.02		0	
Metzelthin, 2015	Healthcare	All	14004.37	13130.56	1	
Metzelthin, 2015	Healthcare	Clinician	1971.69	2346.64	1	Aggregated clinicians and allied professionals into clinican
Metzelthin, 2015	Healthcare	Hospital	1508.73	4168.8	1	
Metzelthin, 2015	Personal	All	7510.89	15080.3	1	
Metzelthin, 2015	Personal	Home-Help Indirect	7131.37	15017.2	1	
Metzelthin, 2015	Personal	Medications	2844.13	4676.36	1	Aggregated medications with aids/in-home modifications
Metzelthin, 2015	Societal	Home Healthcare	6882.16	8200.89	1	
Metzelthin, 2015	Total	All	21515.26	21108.76	1	
Michalowsky, 2016	Personal	Home-Help Indirect	13454.62	13914.46	1	
Michalowsky, 2016	Societal	Home Healthcare	927.85	2382.51	1	
Miller, 2005	Healthcare	Clinician	147.56	151.01	1	
Miller, 2005	Healthcare	Hospital	6835	3280.01	1	Aggregated inpatent admission, inpatient readmission, and day hospital
Miller, 2005	Healthcare	Outpatient	510.84	817.15	1	Aggregated Early Discharge Rehab and Outpatient Visists
Miller, 2005	Societal	Assisted Services	1212.51	168.66	1	Aggregated social services and community health services
Miller, 2005	Total	All	9973.02	11170.98	1	
Mintzer, 1997	Healthcare	All	26414.8	8758.97	0	
Mitchell, 2019	Healthcare	All	32384.97		0	
Murray, 2003	Healthcare	Hospital	8368.48		0	
Nikolaus, 1999	Healthcare	Clinician	147112.8		0	Aggregated Medical staff and physician visits

Nikolaus, 1999	Healthcare	Hospital	28811.99		0	Aggregated initial and readmissions
Nikolaus, 1999	Societal	Assisted Services	2594.46		0	
Nikolaus, 1999	Total	All	37737.72		0	
Pinedo-Villanueva, 2019	Healthcare	Clinician	1307.99	328.44	0	Aggregated Primary care, formal care, and inpatient secondary
Pinedo-Villanueva, 2019	Healthcare	Outpatient	82.05	18.35	0	
Pinedo-Villanueva, 2019	Personal	Home-Help Indirect	854.34	185.38	0	
Pinedo-Villanueva, 2019	Personal	Medications	232.59	82.9	0	
Pinedo-Villanueva, 2019	Total	All	2485.86	378.04	0	
Pitkala, 2013	Healthcare	Clinician	1128.12		0	Aggregated primary care physician, home visits, nurse visits, physiotherapy, and other professionals
Pitkala, 2013	Healthcare	Hospital	8349.41		0	Aggregated primary care hospital, specialized care hospital
Pitkala, 2013	Personal	Home-Help Indirect	793.44		0	
Pitkala, 2013	Societal	Assisted Services	3036.79		0	Aggregated day care center and respite care in institutions
Pitkala, 2013	Societal	Home Healthcare	24.9		0	
Pitkala, 2013	Societal	Outpatient	28.01		0	
Pitkala, 2013	Total	All	17883.75		0	
Reuben, 2004	Healthcare	Hospital	8860.06		1	
Rigaud, 2002	Healthcare	All	768.86		1	
Rigaud, 2002	Personal	Direct Health Care Related Costs	768.86		1	
Rigaud, 2002	Personal	Home-Help Indirect	2176.17		1	
Rigaud, 2002	Total	All	3594.96		1	
Rojas, 2010	Healthcare	All	1170.43	1081.85	0	
Rojas, 2010	Healthcare	Clinician	38.36	50.3	0	
Rojas, 2010	Healthcare	Hospital	371.23	1055.65	0	
Rojas, 2010	Healthcare	Outpatient	206.77	443.77	0	
Rojas, 2010	Personal	Medications	168.26	289.66	0	
Ruchlin, 2001	Healthcare	All	19801.49	15959.28	0	

Ruchlin, 2001	Healthcare	Clinical	5987.08	13872.16	0	Formal help assumed to be clinical
Ruchlin, 2001	Personal	Home-Help Indirect	743.86	2857.35	0	
Ruchlin, 2001	Total	All	26703.96	28890.71	0	
Ruikes, 2018	Healthcare	Clinician	801.23	189.55	1	Aggregated GP and Physiotherapy
Ruikes, 2018	Healthcare	Hospital	1234.91	336.47	1	
Ruikes, 2018	Healthcare	Outpatient	241.04	28.41	1	
Ruikes, 2018	Personal	Home-Help Indirect	1282.41	90.56	1	
Ruikes, 2018	Personal	Medications	1151.9	100.43	1	
Ruikes, 2018	Societal	Day care	339.13	90.11	1	
Ruikes, 2018	Societal	Home Healthcare	2884.85	370.65	1	
Ruikes, 2018	Total	All	8296.34	4038.09	1	
Schousboe, 2019	Healthcare	All	7659.04	14153.21	1	
Schousboe, 2019	Healthcare	Outpatient	2518.04	3256.74	1	
Schraeder, 2008	Healthcare	All	1464.41	2397.31	0	Assume costs are 2008
Schwardzkopf, 2011	Healthcare	Clinician	10536.59		0	Aggregated formal care, non-physician, and physician visits
Schwardzkopf, 2011	Healthcare	Hospital costs	3797.09		0	
Schwardzkopf, 2011	Healthcare	Outpatient	206.64		0	
Schwardzkopf, 2011	Personal	Home-Help Indirect	37025.01		0	
Schwardzkopf, 2011	Personal	Medications	1873.32		0	Aggregated medications and medical aids and assistive devices
Schwardzkopf, 2011	Societal	All	46364.45		0	
Scott, 2004	Healthcare	All	7784.24	22776.07	0	
Scott, 2004	Healthcare	Clinician	1746.3	3115.94	0	
Scott, 2004	Healthcare	Hospital	5665.57	14322.1	0	
Scott, 2004	Healthcare	Medications	1263.61	1704.93	0	
Scott, 2004	Healthcare	Outpatient	4589	6909.36	0	Aggregated CHCC clinic and hospital outpatient costs
Scott, 2004	Societal	Home Healthcare	519.76	1386.01	0	
Taylor, 2003	Healthcare	All	8582.06		0	
Taylor, 2003	Personal	Home-Help Indirect	14577.53		0	Aggregated caregiving for IADLs and ADLs
Taylor, 2003	Personal	Medications	1252.97		0	
Taylor, 2003	Total	All	25148.04		0	
Van Lier, 2016	Healthcare	Clinical	73.34	140.01	1	
Van Lier, 2016	Healthcare	Hospital	782.12	2627.18	1	

Van Lier, 2016	Personal	Home-Help Indirect	2393.01	5232.27	1	
Van Lier, 2016	Societal	Home Healthcare	2071.74	2235.5	1	
Van Lier, 2016	Societal	All	5581.04	6520.65	1	
Van Lier, 2016	Societal	Assisted Services	166.26	231.68	1	
Wang, 2008	Healthcare	All	2569.56	2212.94	0	
Wang, 2008	Personal	Home-Help Indirect	4814.73	3739.98	0	
Wang, 2008	Personal	Medications	2370	2235.15	0	
Wang, 2008	Total	All	8656.76	5028.4	0	
Wang, 2010	Personal	Home-Help Indirect	3958.69	6853.14	1	
Witham, 2012	Healthcare	All	1607.86	3214.74	1	
Witham, 2012	Healthcare	Clinician	128.65	130.07	1	
Witham, 2012	Healthcare	Hospital	876.16	3025.52	1	
Witham, 2012	Healthcare	Outpatient	105.95	104.48	1	
Witham, 2012	Personal	Medications	273.44	290.35	1	
Wolff, 2019	Healthcare	All	5149.22		1	
Wolff, 2019	Healthcare	Clinician	2743.93	4859.84	1	
Wolff, 2019	Healthcare	Outpatient	1229.87	2905.61	1	
Wolff, 2019	Societal	Home Healthcare	5099.64	9444.17	1	
Wolstenholme, 2002	Total	All	19527.51	12350.23	0	
Woods, 2012	Healthcare	Clinician	197.58	504.83	0	
Woods, 2012	Healthcare	Hospital	2201.9	5819.09	0	
Woods, 2012	Societal	All	940.68	1591	0	
Woods, 2012	Societal	Assisted Services	716.62	2461.19	0	Aggregated district nurse, health visitor, day care, and local authority home-care worker
Wubker, 2014	Societal	All	2140.67	9019.3	1	Aggregated across all countries (Estonia, Finland, France, Germany, Holland, England, Spain)
Zhang, 2007	Healthcare	Clinician	5339.76		0	Assumed 1 year time length
Zhang, 2007	Healthcare	Hospital	13894.79		0	Assumed 1 year time length
Zhang, 2007	Healthcare	Outpatient	1508.63		0	Assumed 1 year time length
Zhang, 2007	Societal	Home Healthcare	4178.51		0	Assumed 1 year time length
Zhang, 2007	Total	All	27712.4		0	Assumed 1 year time length
